# EndoMAP.v1 charts the structural landscape of human early endosome complexes

**DOI:** 10.1038/s41586-025-09059-y

**Published:** 2025-05-28

**Authors:** Miguel A. Gonzalez-Lozano, Ernst W. Schmid, Enya Miguel Whelan, Yizhi Jiang, Joao A. Paulo, Johannes C. Walter, J. Wade Harper

**Affiliations:** 1 Department of Cell Biology, Harvard Medical School, Boston, MA USA; 2 Aligning Science Across Parkinson’s (ASAP) Collaborative Research Network, Chevy Chase, MD USA; 3 Department of Biological Chemistry and Molecular Pharmacology, Harvard Medical School, Boston, MA USA; 4 Initiative for Genome Editing and Neurodegeneration, Department of Cell Biology, Harvard Medical School, Boston, MA USA; 5 Howard Hughes Medical Institute, Boston, MA USA

**Keywords:** Endosomes, Proteomics

## Abstract

Early or sorting endosomes are dynamic organelles that play key roles in proteome control by triaging plasma membrane proteins for either recycling or degradation in the lysosome^1,2^. These events are coordinated by numerous transiently associated regulatory complexes and integral membrane components that contribute to organelle identity during endosome maturation^3^. Although a subset of the several hundred protein components and cargoes known to associate with endosomes have been studied at the biochemical and/or structural level, interaction partners and higher-order molecular assemblies for many endosomal components remain unknown. Here, we combine crosslinking and native gel mass spectrometry^4–7^ of purified early endosomes with AlphaFold^8,9^ and computational analysis to create a systematic human endosomal structural interactome. We present 229 structural models for endosomal protein pairs and additional higher-order assemblies supported by experimental crosslinks from their native subcellular context, suggesting structural mechanisms for previously reported regulatory processes. Using induced neurons, we validate two candidate complexes whose interactions are supported by crosslinks and structural predictions: TMEM230 as a subunit of ATP8 and ATP11 lipid flippases^10^ and TMEM9 and TMEM9B as subunits of the chloride–proton antiporters CLCN3, CLCN4 and CLCN5 (ref. ^11^). This resource and its accompanying structural network viewer provide an experimental framework for understanding organellar structural interactomes and large-scale validation of structural predictions.

## Abstract (web summary)

A study presents EndoMAP.v1, a resource that combines information on protein interactions and crosslink-supported structural predictions to map the interaction landscape of early endosomes.

## Main

Plasma membrane protein flux is controlled, in part, through a series of membrane-bound organelles referred to as the endolysosomal system^1^. Endocytic vesicles bud from the plasma membrane and rapidly undergo conversion to RAB5^+^ vesicles referred to as early or sorting endosomes or, for simplicity, early endosomes^2^. Early endosomes serve as platforms for plasma membrane protein sorting and recycling while also receiving regulatory proteins through fusion with Golgi-derived transport vesicles^12^. Dynamic maturation of RAB5^+^ early endosomes to RAB7^+^ late endosomes accompanies ESCRT-mediated trafficking of plasma membrane proteins into intraluminal vesicles, facilitating their degradation following maturation into lysosomes^1,3^. Lysosomes also function in the elimination of intracellular proteins and organelles through autophagy^13^.

Our understanding of the endolysosomal system has been facilitated through the identification of functional modules involved in vesicle fusion, cargo trafficking and organelle maturation^1–3,14^, some of which are associated with neurodegenerative and lysosomal storage disorders^15–17^. However, the dynamic nature of these organelles has made some protein assignments controversial^18,19^, and particular protein assemblies that are dependent on interaction with the endosomal membrane may be lost in the context of conventional co-immunoprecipitation (co-IP) approaches in which membrane integrity is disrupted. Consequently, gaps exist in our understanding of the proteins, complexes and structures across various endolysosomal subpopulations.

**Fig. 1:**
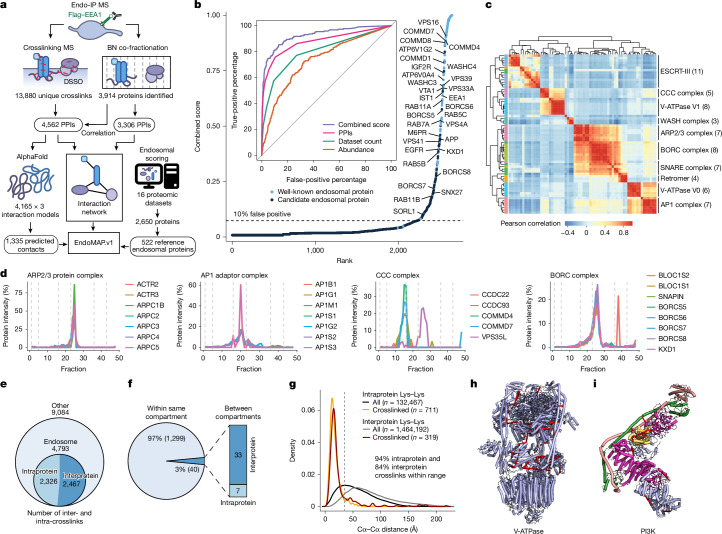
EEA1^+^ endosomal proteome analysis through dual complexomics strategies. **a**, EndoMAP.v1 workflow schematic depicting integration of XL–MS, BN–MS, scoring method and structural predictions to create an endosomal protein complex structural interaction landscape. **b**, Endosomal scoring method; known (blue) and candidate (black) endosomal proteins ranked on the basis of combined scoring method, with higher values indicating higher probability of a protein being endosomal. The inset shows receiver operating characteristic curves for each individual metric and its combination for annotating endosomal proteins. Partial area under the curve values at 10% false-positive percentage: combined score, 6.9%; PPIs, 6.1%; dataset count, 4.0%; abundance, 2.1%. **c**, Correlation heat map of BN–MS co-fractionation data showing unsupervised clustering of well-known endosomal complexes. Number of proteins included in each complex is indicated in brackets. **d**, Co-fractionation profiles of selected protein complexes from BN–MS. **e**, Summary of DSSO crosslinks identified in Endo-IP samples, including intraprotein and interprotein crosslinks involving high-confidence endosomal proteins. **f**, Pie chart showing the number of DSSO crosslinks within and between topological compartments based on Uniprot. **g**, Density plots showing the distribution of Cα–Cα distances (Å) for intraprotein and interprotein DSSO crosslinks for all structures available in the PDB for the entire XL–MS dataset. The vertical dashed line indicates the maximum distance allowed by the crosslinker. **h**,**i**, Identified DSSO crosslinks (red lines) mapped into the endolysosomal V-ATPase (**h**, PDB 6WM2)^59^ and the class II PI3P lipid kinase complex (**i**, PDB 7BL1)^27^. Panel **a** adapted from ref. ^44^, CC BY 4.0. Source data

Here we present EndoMAP.v1, a structural protein interactome of human early endosomes. We focused on an endosomal subpopulation characterized by association with early endosome antigen 1 (EEA1), used here as an organelle isolation handle through early endosomes (Endo-IP)^19^. EndoMAP.v1 combines crosslinking–mass spectrometry (XL–MS)^4–6,20^ and blue-native polyacrylamide gel co-fractionation–MS (BN–MS) to generate a comprehensive network of protein interactions and candidate complexes in EEA1-associated endosomes (Fig. 1a). Large-scale AlphaFold Multimer (AF-M)^8,9^ and AlphaLink2 (ref. ^21^) analysis across the network generated 229 structural predictions supported by crosslink distance constraints, which are available via the EndoMAP.v1 structural interactome viewer (https://endomap.hms.harvard.edu/). We demonstrated the value of this resource: through validation of transmembrane subunits of endosomal lipid flippases and chloride–proton (Cl^−^–H^+^) antiporters; and through crosslink-informed structural predictions of dozens of protein interactions and multiprotein assemblies across diverse core endosomal functional categories. EndoMAP.v1 provides a resource for mechanistic analysis of early endosome complexes and an experimental framework for understanding structural interactomes for specific organelles.

## Dual complexomics approaches

**Extended Data Fig. 1:**
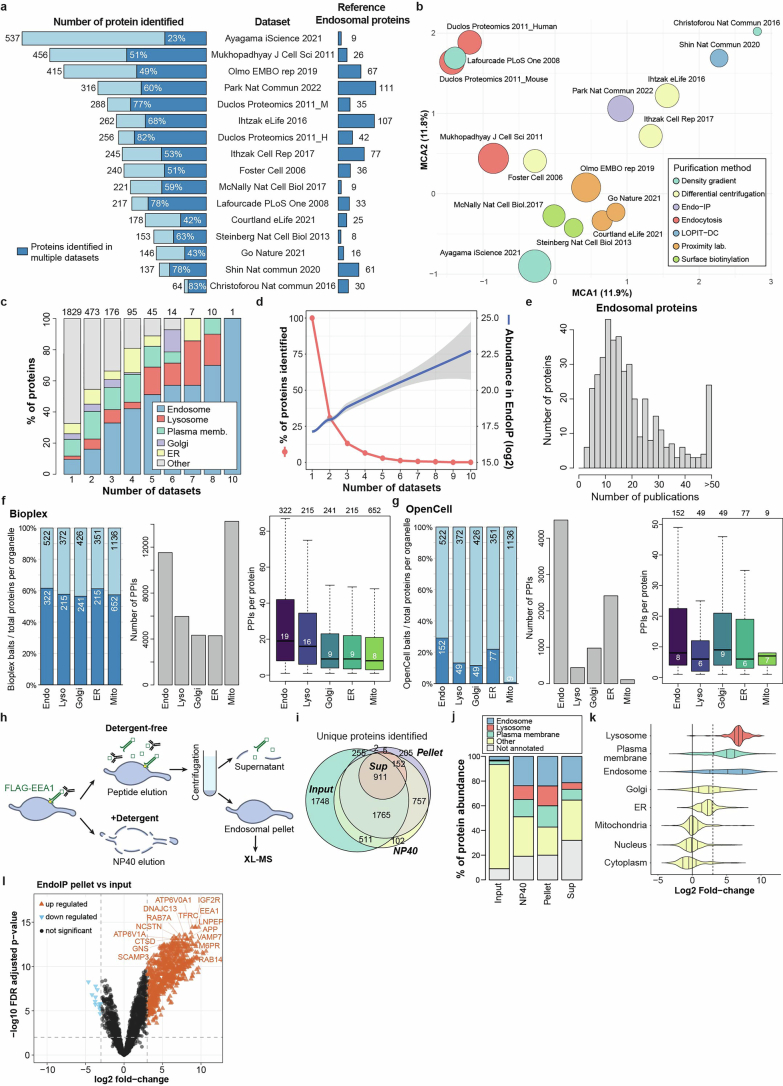
Endosomal proteome scoring method and optimization of large-scale Endo-IP. **a**, Overview of datasets used for endosomal scoring, including the number of proteins identified in each and across datasets, and the number of well-known endosomal proteins identified in the indicated studies. **b**, Multiple correspondence analysis (MCA) showing an overview of the relationship among datasets from **a**. Each node represents a dataset color-coded by isolation method and proportional size to the total number of proteins identified. **c**, Bar plot depicting the number of proteins identified across multiple datasets for several subcellular compartments. **d**, Line graph showing the percentage of proteins identified across all 16 datasets in **a** and their protein abundance in our Endo-IP experiments from HEK293 cells (Supplementary Table 1) represented as loess (Locally Estimated Scatterplot Smoothing) regression line and 95% confidence level interval band. **f,g**, Number of bait proteins, protein-protein interactions (PPIs), and PPIs per protein in Bioplex^28^ (panel **f**) and Open Cell^119^ (panel **g**) according to organelle assignment (n number of proteins in each category is indicated on top). For box plots, the middle line corresponds to the median, the lower and upper end of the box correspond respectively to the first and third quartiles, and the whiskers extend from the box to 1.5 times the inter-quartile range. **h**, Schematic of the purification steps from Endo-IP to endosomal pellet used for complexomics. **i,j**, Number of proteins identified (panel **i**) and abundance per compartment (panel **j**) in endosomal pellet compared to the input (PNS), supernatant, or NP40 eluate from the Endo-IP as depicted in panel **h**. **k**, Violin plot showing the fold-change enrichment of proteins from individual organelle compartments in endosomal pellets compared to input (PNS). **l**, Volcano plot showing fold-changes and FDR adjusted p-value for proteins in endosomal pellets compared to input (PNS) (n = 3 biologically independent replicates). DEqMS algorithm was used for statistical analysis with multiple testing correction as implemented in^146^. **h**, Images modified from ref. ^44^ (Copyright (2024) National Academy of Sciences). Source data

To understand protein interactions associated with EEA1^+^ endosomes, we developed an experimental and informatic complexomics pipeline (Fig. 1a). We first defined and characterized the endosomal proteome by analysing 16 previous experimental studies reporting endosomal proteins using diverse purification strategies and cell types (Extended Data Fig. 1a–g and Supplementary Text). The combination of three predictors (frequency of identification, protein abundance and interaction with endosomal proteins) best captured many well-characterized endosomal proteins with high confidence (Fig. 1b). This analysis identified 522 known and predicted endosomal proteins on the basis of experimental data (Supplementary Table 1), with these proteins serving as a reference endosomal proteome for further characterization with our complexomics pipeline.

**Extended Data Fig. 2:**
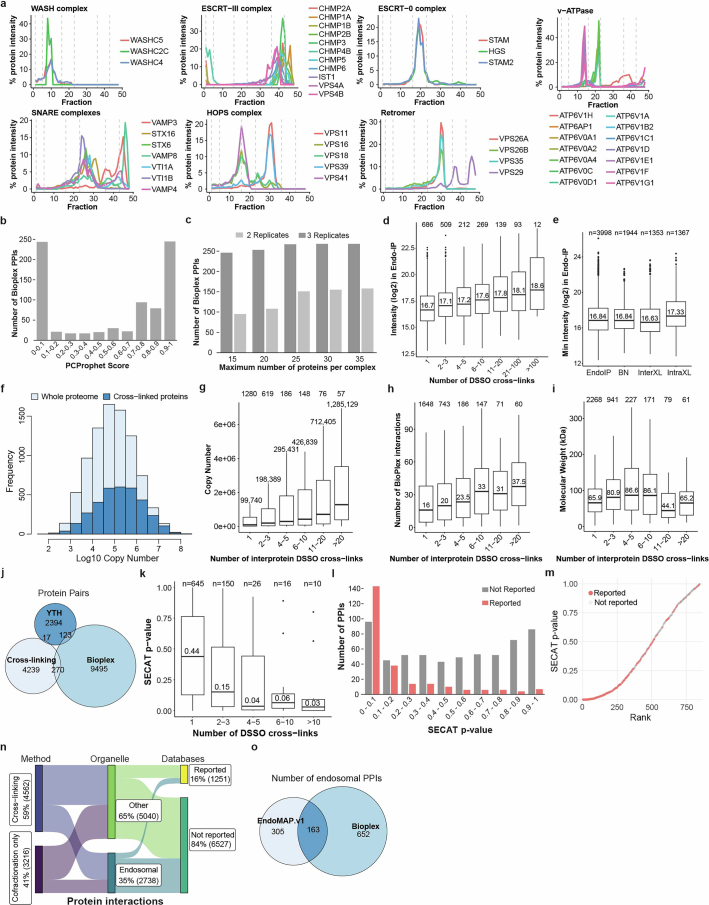
Application of correlation profiling and cross-linking proteomics to endosomes purified by Endo-IP. **a**, Co-fractionation profiles of selected protein complexes from BN-MS. **b**, Number of Bioplex interactions identified by BN-MS compared to co-fractionation PCProphet scores. **c**, Number of Bioplex interactions identified using PCProphet in either 2 or 3 replicates of the Endo-IP BN-MS compared to the maximum number of proteins per complex allowed in the analysis. **d**, Box plot depicting the protein MS signal intensity in Endo-IP compared to the number of DSSO cross-links identified for each protein (n number of proteins in each category is indicated on top). **e**, Box plot depicting the minimum protein MS signal intensity for PPIs identified by BN and DSSO cross-linking compared to all proteins identified in Endo-IP (n number of interactions in each category is indicated on top). **f**, Distribution of protein copy number (log_10_)^18^ for cross-linked proteins compared to the whole proteome. **g-i**, Box plots depicting the protein copy number (**g**), number of interactors in BioPlex (**h**), and molecular weight (**i**) compared to the number of interprotein DSSO cross-links identified for each protein (n number of proteins in each category is indicated on top). **j**, Venn diagram showing the number of protein pairs identified by yeast two hybrid (YTH), Bioplex, and cross-linking proteomics for the same set of proteins. **k**, Boxplot showing co-fractionation SECAT p-values for cross-linked proteins identified by different number of DSSO cross-links (n number of interactions in each category is indicated on top). **l,m**, Number (panel **l**) and rank (panel **m**) of cross-linked protein interactions that have been previously reported (or not) compared to their co-fractionation SECAT p-value (Supplementary Table 2). SECAT was used for statistical analysis^169,170^. **n**, Overview of protein interactions within EndoMAP.v1 including the method, organelle and previous reports. **o**, Venn diagram showing the overlap of endosomal interactions between EndoMAP.v1 and Bioplex for interactions in which both proteins are present in both datasets. For all box plot panels, the middle line corresponds to the median, the lower and upper end of the box correspond respectively to the first and third quartiles, and the whiskers extend from the box to 1.5 times the inter-quartile range. Source data

We then used BN–MS and crosslinking by XL–MS to identify candidate protein–protein interactions (PPIs; Fig. 1a). We further optimized and extensively evaluated the Endo-IP approach in HEK293 cells^19^, with early endosomes eluted from the affinity matrix under detergent-free conditions for XL–MS or using detergent for BN–MS (Methods and Extended Data Fig. 1h–l). Triplicate Endo-IP samples were fractionated by BN gel electrophoresis, and 48 individual fractions across all mass ranges were subjected to MS analysis, identifying 3,914 unique proteins (Supplementary Table 2). Numerous well-characterized endosomal protein complexes were found to co-fractionate on the basis of Pearson coefficients of normalized elution profiles (Fig. 1c). These include the BLOC-one-related complex (BORC) involved in endolysosomal positioning^22^, components of the homotypic fusion and protein sorting (homotypic fusion and protein sorting (HOPS)) complex^23^, and the AP1 adaptor complex that traffics cargo to endolysosomes^24^, among others (Fig. 1c,d and Extended Data Fig. 2a). Unbiased correlation profiling using PCProphet^25^ revealed the presence of 3,306 candidate interacting proteins pairs. To recover high-confidence candidate interactions, we considered only interactions with a score of at least 0.7 in two replicates, which maximized the recovery of interactions reported in Bioplex (Methods, Extended Data Fig. 2b,c and Supplementary Table 2).

In parallel, duplicate matrix- and detergent-free Endo-IP samples were crosslinked using the MS-cleavable disuccinimidyl sulfoxide (DSSO) Lys–Lys crosslinker and analysed by XL–MS to identify proximal protein pairs in intact organelles^4,6^ (Fig. 1a). We identified 13,877 unique DSSO crosslinks, of which 4,793 involved intraprotein or interprotein crosslinks among our reference endosomal proteins (inclusive of the EEA1 endosomal purification handle; Fig. 1e and Supplementary Table 2). A total of 97% of the crosslinks matched the expected topological connectivity (within cytosolic, luminal or extracellular regions), consistent with the purification of intact organelles with Endo-IP (Fig. 1f). This is within the range of the 5% false-discovery rate (FDR) used for crosslink identification. To evaluate the quality and specificity of crosslinking across the full dataset (including all non-endosomal proteins), we compared 1,030 crosslinked Lys(Cα)–Lys(Cα) distances for all available Protein Data Bank (PDB) structures (219 in total). Most intraprotein (94%) and interprotein (84%) crosslinks were within the 35-Å maximum distance for DSSO crosslinker (considering in-solution flexibility^26^; Fig. 1g). Representative endosomal multiprotein complexes (V-ATPase and the class II PI3 kinase PIK3C3–BECN1–UVRAG–PIK3R4)^27^ are shown in Fig. 1h,i, with multiple crosslinks among proteins within each complex. Although there is mild bias towards more abundant proteins (Extended Data Fig. 2d,e), crosslinks are detected across the complete span of protein copy number (Extended Data Fig. 2f). In terms of PPIs, proteins with a higher number of crosslink-supported interactions were correlated with copy number and number of interactions in BioPlex^28^, but not with molecular weight (Extended Data Fig. 2g–i), as previously observed^6^. Limited overlap between crosslinked pairs and interaction pairs reported in BioPlex^28^ or yeast two-hybrid datasets^29^ is consistent with the maintenance of weaker interactions in the context of organelle crosslinking (Extended Data Fig. 2j). Interactions with higher numbers of crosslinks have better co-elution Size-Exclusion Chromatography Algorithmic Toolkit (SECAT) *P* values in BN (Extended Data Fig. 2k and Supplementary Table 2). Additionally, previously reported crosslinked interactions have a better co-elution SECAT *P* value than new candidate interactions, which most likely include interactions that are transient and difficult to identify by other methods (Extended Data Fig. 2l,m and Supplementary Table 2). In sum, we identified a total of 4,562 and 3,306 protein interactions by crosslinking and BN–MS, respectively, which provide a useful dataset for exploration of early endosome protein interactions (Extended Data Fig. 2n,o and Supplementary Table 2).

## Early endosome interaction landscape

**Fig. 2:**
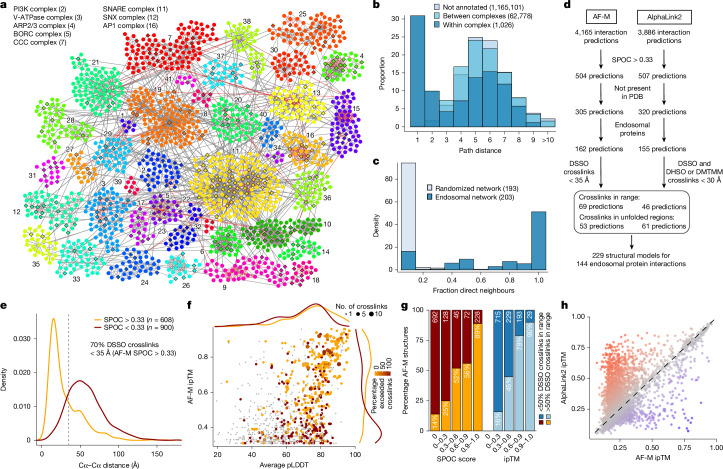
Assembly of an integrated endosome protein complex structural landscape. **a**, Core component of the network containing 1,722 nodes organized into 41 communities (indicated by numbers) and 3,489 edges. Significantly enriched protein complexes of selected communities are provided in the top left (see Supplementary Table 2 for full list of communities). Diamonds and circular nodes represent high-confidence endosomal and other proteins, respectively. Solid and dashed edges represent interactions identified by at least one crosslink or only co-fractionating, respectively. Red edges indicate interaction previously reported. **b**, Distribution of path distances between proteins within and between the same complex compared with proteins without complex annotation. **c**, Distribution of fraction of direct neighbours in the same complex for each protein compared with a randomized network control. **d**, Systematic AF-M and AlphaLink2 predictions of protein interactions identified by XL–MS and match with the crosslink distance constraints. **e**, Distribution of Cα–Cα distances (Å) for interprotein DSSO crosslinks reflecting AF-M predictions with SPOC > 0.33 (orange) and SPOC < 0.33 (red). Only residues with pLDDT > 70 were considered. **f**, Distribution of AF-M ipTM scores and average pLDDT for predictions with ipTM > 0.3. Numbers of interprotein crosslinks evaluated and exceeding the DSSO crosslinker distance constraints are indicated by point size and the colour, respectively. **g**, Percentage of pairwise AF-M predictions with more or fewer than 50% of crosslinks within the distance constraint (orange and red, respectively) relative to the SPOC and ipTM score. **h**, ipTM scores for AF-M compared with AlphaLink2 predictions. Colour gradient represents the score difference; higher in AlphaLink2 (red) or AF-M (blue). Source data

**Extended Data Fig. 3:**
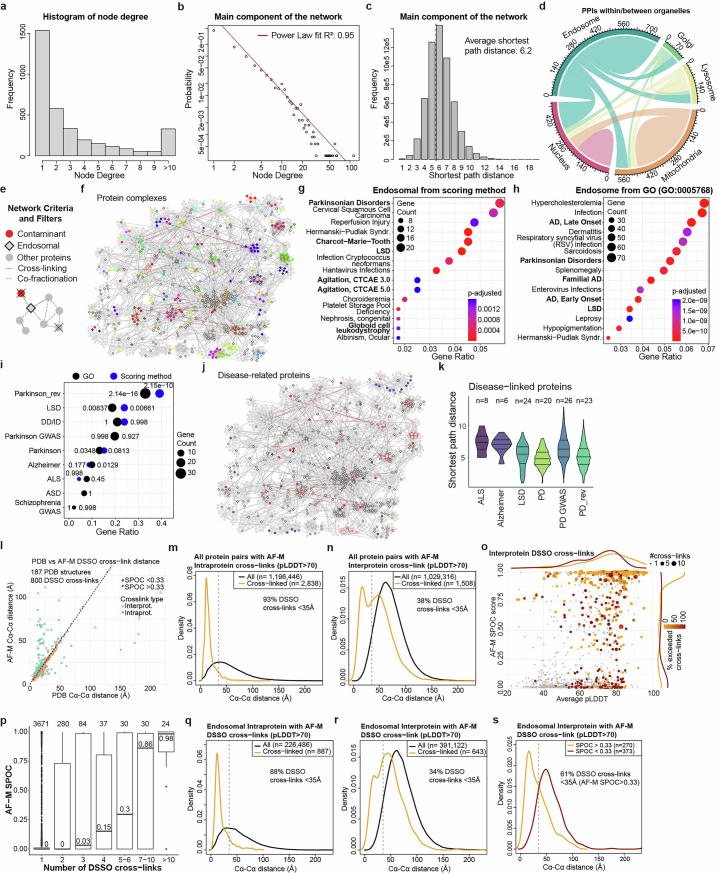
EndoMAP.v1 network characterization and application of AlphaFold-M across DSSO cross-linked protein pairs. **a**, Degree distribution (number of edges per node) of the complete network. **b**, Power law log-log plot of the complete network showing the degree of a node (number of edges) and the probability. **c**, Distribution of the shortest path distances between all proteins in the complete interaction network. **d**, Distribution and number of PPIs within and between selected organelles (Supplementary Table 2). **e**, Criteria for network filtering to create an integrated endosomal network (EndoMAP.v1, see **METHODS**). **f**, Mapping of known protein complexes from CORUM^126^ onto the core components of the EndoMAP.v1 network (Supplementary Table 2). **g,h**, DisGeNET enrichment analysis of endosomal proteins as defined by our scoring method (panel **g**) and Gene Ontology (GO:0005768, panel **h**). Top 15 categories by highest gene ratio are depicted. Disorders related to the nervous system are indicated in bold. p-values by hypergeometric test were adjusted with Benjamini-Hochberg correction. **i**, Enrichment analysis of the endosomal proteome within several neurodegenerative diseases (LSD, Lysosomal Storage Disorders; ALS, Amyotrophic Lateral Sclerosis, PD, Parkinson’s disease; ASD, Autism Spectrum Disorders; DD/ID, epilepsy and severe neurodevelopmental disorder). **j**, Mapping of neurodegenerative disease related proteins onto the core component of EndoMAP.v1 network (see **METHODS**, Supplementary Table 2). **k**, Distribution of shortest path distances within various classes of neurodegenerative disease related proteins. Three different sources of disease genes were used to retrieve proteins related to PD (see **METHODS**). **l**, Distances between DSSO cross-linked lysines for AF-M predictions compared to structures in the PDB. Green and orange dots represent interprotein and intraprotein cross-links, respectively. Filled and empty dots represent predictions with SPOC > 0.33 or SPOC < 0.33, respectively. **m**, Distribution of Cα-Cα distances (Å) for intraprotein DSSO cross-linked lysines in all AF-M predictions compared to all lysines. **n**, Distribution of Cα-Cα distances (Å) for interprotein DSSO cross-linked lysines in all AF-M predictions compared to all lysines. **o**, Distribution of SPOC scores and average pLDDT for predictions with SPOC > 0. Number of interprotein DSSO cross-links evaluated and exceeding the cross-linker distance restrain are indicated by point size and the color, respectively. **p**, Box plot showing the distribution of SPOC scores relative to the number of DSSO cross-links identified for each interaction (n number of interactions in each category is indicated on top). The middle line corresponds to the median, the lower and upper end of the box correspond respectively to the first and third quartiles, and the whiskers extend from the box to 1.5 times the inter-quartile range. **q,r**, Distribution of Cα-Cα distances (Å) for intraprotein (**q**) and interprotein (**r**) DSSO cross-linked lysines in AF-M predictions involving endosomal proteins compared to all lysines. **s**, Distribution of Cα-Cα distances (Å) for interprotein DSSO cross-links reflecting predictions involving endosomal proteins with SPOC > 0.33 (orange) and SPOC < 0.33 (red). Source data

To create an early endosome interaction map, we integrated XL–MS and BN–MS data with our reference endosomal proteome, applying stringent filters (Methods). The resulting network exhibited an average shortest path distance of 6.2 and followed a power-law distribution with *R*^2^ > 0.95 (Extended Data Fig. 3a–c). Exploring the connectivity between localization descriptors, we found that endosomal proteins were highly connected with other endosomal proteins or proteins annotated as lysosomal or Golgi (88% of the endosomal interactions), with notably fewer connections with other organelles (that is, mitochondria or nucleus; Methods and Extended Data Fig. 3d). Additional filtering, including centring the network around our reference endosomal proteome and filtering of doubtful connectivity (that is, nuclear proteins, which correspond to up to 8.5% of the interactions with endosomal proteins; Methods and Extended Data Fig. 3e), yielded a network containing 1,933 nodes and 4,282 edges. The core component of the network (without disconnected modules) included 1,722 protein and 3,489 interactions organized in 41 communities, which were significantly enriched for several well-known endosomal complexes, including V-ATPase, soluble *N*-ethylmaleimide-sensitive factor attachment protein receptor (SNAREs) and the CCDC22, CCDC93 and COMMD (CCC) complex (Fig. 2a and Supplementary Table 2). Indeed, proteins belonging to the same known complex were closer and in direct contact within the network (Fig. 2b,c and Extended Data Fig. 3f). Through an unbiased enrichment analysis of all disease pathways in DisGenNET, we found that Parkinson’s disease-related genes were the most highly enriched in our reference endosomal proteome (Extended Data Fig. 3g–i and Supplementary Table 1). Proteins associated with other neurodegenerative disorders were also enriched, including lysosomal storage disorder proteins, many of which are actively trafficked to early endosomes^15,16^. Proteins linked with these disorders exhibit the shortest path distance (about 5.0, reflective of their density within the network (Extended Data Fig. 3j,k). As elaborated below, this network provides a discovery platform for understanding the interaction landscape of early endosomes.

## Large-scale AlphaFold predictions

To transform the endolysosomal network into a structurally informed interactome, we performed large-scale AF-M predictions^8,9^. We analysed 4,165 protein pairs identified by XL–MS (total residue length <3,600 amino acids owing to computational constraints), including both endosomal and non-endosomal protein pairs. We ranked each pair using a Structure Prediction and Omics-informed Classifier (SPOC)^30^ to evaluate complex plausibility (Supplementary Table 3). SPOC considers interface predicted template modelling (ipTM) and predicted aligned error (PAE) scores of the predicted interface (among other metrics) together with biological correlations among the interacting proteins (such as co-localization and genetic co-dependency) and scores above 0.33 (scale 0–1) can indicate direct interactions^30^. We then independently assessed the reliability of the predictions by evaluating the extent to which structural predictions were consistent with DSSO crosslink distance constraints (Fig. 2d and Supplementary Table 3). As expected, there was a strong correlation between distances in AF-M predictions and the corresponding structures in the PDB, both for intraprotein and interprotein crosslinks (Extended Data Fig. 3l). Moreover, within all pairwise predictions, 93% and 38% of intraprotein and interprotein DSSO crosslink distances, respectively, were within range (<35 Å)^26^ (Extended Data Fig. 3m,n). In the latter case, the bi-modal distribution was largely explained by protein pairs for which AF-M was unable to predict an interaction (SPOC < 0.33), as 70% of interprotein crosslink distances were within range for pairs with SPOC > 0.33 (Fig. 2e). The fraction of predictions with interprotein crosslinks satisfying the length requirements correlated with the SPOC and ipTM scores (Fig. 2f,g). We also observed a correlation between the number of crosslinks identified for an interaction and its prediction SPOC score (Extended Data Fig. 3o,p). Predictions involving at least one endosomal protein had a similar distribution of crosslink matches as predictions from the full dataset (Extended Data Fig. 3q–s). Therefore, SPOC scores and crosslinking data are complementary approaches that provide structural and experiment support to the interactions identified in EndoMAP.v1. With AF-M, we obtained 162 unique, endosomal pairwise structural predictions not present in the PDB with SPOC > 0.33, including 69 structures matching interprotein crosslink constraints, 53 with crosslinks in unstructured regions and 40 structures not matching crosslink constraints (Fig. 2d).

**Extended Data Fig. 4:**
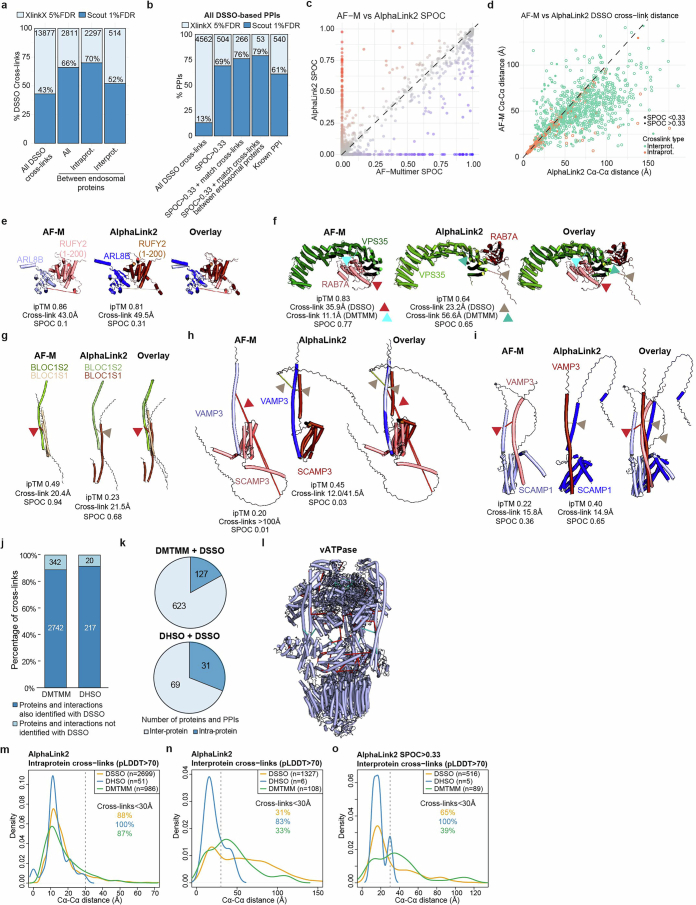
EndoMAP.v1 extension by AlphaLink2 and XL-MS using DHSO/DMTMM cross-linkers. **a**, Overlap of DSSO cross-linking data analyzed using XlinkX at 5% FDR compared to Scout at 1%FDR. **b**, Number of protein interactions based on DSSO cross-links identified with XlinkX and Scout for known interactions and across the selection criteria used in EndoMAP.v1 (i.e. filtering for AF-M score, endosomal protein and cross-link distance). **c**, ipTM scores for AF-M compared to AlphaLink2 predictions. Color gradient represents the score difference; higher in AlphaLink2 (red) or AF-M (blue). **d**, Distances between DSSO cross-linked lysines for AF-M compared to AlphaLink2 predictions. Green and orange dots represent interprotein and intraprotein cross-links, respectively. **e-i**, Individual and overlay AF-M and AlphaLink2 predictions for several protein pairs (see Supplementary Text). DSSO and DHSO/DMTMM interprotein cross-links are indicated with red and cyan lines and arrowheads, respectively. **j**, Mapping DHSO/DMTMM cross-linking data to the proteins and interactions identified with DSSO. **k**, Pie chart showing the number of protein pairs identified with both DMTMM and DSSO (top) or DHSO and DSSO (bottom). **l**, Identified DSSO (red) and DHSO/DMTMM (cyan) cross-links mapped into the endolysosomal V-ATPase (PDB:6WM2)^59^. **m,n**, Distribution of Cα-Cα distances (Å) for intraprotein (**m**) and interprotein (**n**) cross-linked residues in AlphaLink2 predictions. **o**, Distribution of Cα-Cα distances (Å) for interprotein cross-links reflecting predictions with SPOC > 0.33. Source data

Three approaches were used to further strengthen and extend structural modelling in EndoMAP.v1. First, DSSO crosslinking data were evaluated using the recently reported Scout search engine^31^ (Supplementary Table 2). Scout with 1% FDR recovered 43% of those crosslinks identified by XlinkX at 5% FDR, 66% between endosomal proteins (Extended Data Fig. 4a), including most examples described below. Regarding protein interactions, our pipeline filtering criteria substantially increased the overlap with Scout, with up to 79% overlap for the interactions between endosomal proteins with good AF-M predictions (SPOC > 0.33) matching the DSSO crosslink distance constraints (Extended Data Fig. 4b). Nevertheless, Scout recovered only 61% of previously reported interactions identified using XlinkX, suggesting that there is still true connectivity that was missed by the more stringent Scout search (Extended Data Fig. 4b). All interactions found at 1% FDR are indicated in the web portal and Supplementary Table 2. Second, we used AlphaLink2 (ref. ^21^) to generate structural predictions assisted by DSSO crosslink data and compared them to AF-M. We generated predictions for 3,886 protein pairs identified by XL–MS (total residue length <3,000 amino acids owing to computational constraints; Supplementary Table 3). Typically, predictions with strong scores showed comparable ipTM and SPOC for AF-M and AlphaLink2, whereas predictions with AF-M ipTM < 0.3 showed frequently higher AlphaLink2 ipTM score (Fig. 2h and Extended Data Fig. 4c). DSSO crosslink distances were comparable between AF-M and AlphaLink2 predictions, both for intraprotein and interprotein crosslinks (Extended Data Fig. 4d). Several examples illustrate cases of endosomal interactions with score or crosslink distance differences between AF-M and AlphaLink2 (Extended Data Fig. 4e–i and Supplementary Text). Third, we performed an additional Endo-IP XL–MS experiment using alternative crosslinkers (3,3′-sulfinyldi(propanehydrazide) (DHSO) and 4-(4,6-dimethoxy-1,3,5-triazin-2-yl)-4-methylmorpholinium chloride (DMTMM)) to evaluate AlphaLink2 and provide further evidence for AF-M structural predictions. DHSO and DMTMM can crosslink pairs of acidic residues or acidic residues with Lys, respectively^5,32^. We identified 237 and 3,084 crosslinks with DHSO and DMTMM (1% FDR), respectively, which was in the expected range compared to DSSO^32^ (Extended Data Fig. 4j and Supplementary Table 2). Around 90% of the DHSO or DMTMM crosslinks could be mapped to the same proteins and interactions identified with DSSO (69 and 623 interprotein interactions with DHSO and DMTMM, respectively), such as V-ATPase (Extended Data Fig. 4k,l). Within AlphaLink2, 88% and 87% of intraprotein DSSO and DMTMM crosslink distances, respectively, were within range (<30 Å; Methods and Extended Data Fig. 4m). Only 51 DHSO crosslinks could be mapped to structured regions (pLDDT > 70) of AlphaLink2 predictions, all within the distance constraint. For interprotein crosslinks, 65% and 39% of DSSO and DMTMM distances, respectively, were within range for pairs with SPOC > 0.33 (Extended Data Fig. 4n,o). In summary, we obtained 155 endosomal predictions with AlphaLink2 (SPOC > 0.33) that, together with AF-M, make 229 structural predictions for 144 endosomal interactions not present in the PDB that match interprotein crosslink constraints (or with crosslinks in unstructured regions; Fig. 2d). In sum, we generated an experimentally supported structural interactome of the endosomal system.

## TMEM230 as new lipid flippase subunit

**Fig. 3:**
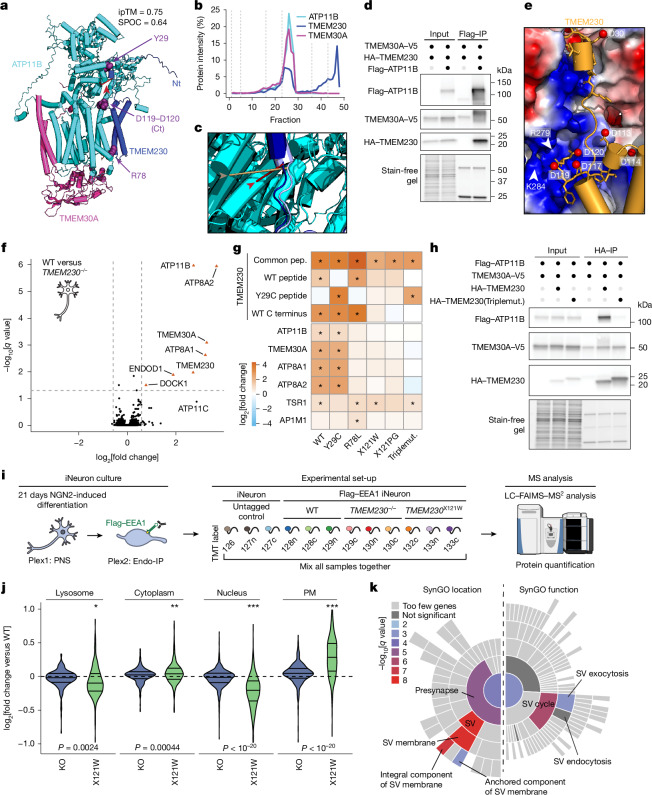
TMEM230 binds endosomal P4 lipid flippases ATP11B, ATP8A1 and ATP8A2 and interface variants disrupt interaction. **a**, AF-M prediction for TMEM230–ATP11B–TMEM30A, in blue, cyan and magenta, respectively. TMEM230 Y29, R78 and C terminus (Ct; D120–D121), as purple space fill, and N terminus (Nt) are indicated. ipTM and SPOC scores are provided for the ATP11B–TMEM230 interaction. **b**, TMEM230–ATP11B–TMEM30A BN–MS profiling. **c**, Overlay of AF-M and AlphaLink2 predictions for TMEM230–ATP11B. AF-M: TMEM230 (dark blue), ATP11B (cyan), crosslink (red line and arrowhead). AlphaLink2: TMEM230 (light blue), ATP11B (teal), crosslink (wheat line and arrowhead). **d**, HA–TMEM230 and Flag–ATP11B co-precipitation after transfection (HEK293 cells). Anti-Flag immunoprecipitates or input samples were immunoblotted for the indicated proteins. **e**, Basic pocket in ATP11B predicted to interact with the acidic TMEM230 C terminus (yellow). Red spheres represent aspartic residues of TMEM230. **f**, Identification of TMEM230-interacting proteins in iNeurons. Volcano plot showing the proteomic analysis of anti-TMEM230 immunocomplexes from WT H9 compared with H9 *TMEM230*^*−/−*^ iNeurons (*n* = 3 biologically independent replicates). **g**, Heat map showing the log_2_[fold changes] in the abundance of all significantly enriched proteins in TMEM230 IPs in H9 *TMEM230*^*−/−*^ iNeurons with or without lentiviral expression of WT and variant HA–TMEM230. Asterisks indicate significantly enriched proteins (*q* value < 0.05, fold change > 1.5). pep., peptide; Triplemut., TMEM230(Y29C/R78L/X121W). **h**, Co-precipitation of HA–TMEM230 and HA–TMEM230(Y29C/R78L/X121W) with Flag–ATP11B and TMEM30A–V5 in transfected HEK293 cells, as examined using immunoblotting of anti-HA immunocomplexes. **i**, Schematic of experimental design for proteomic analysis of early endosomes (TMT multiplex set 2, plex 2) and PNS (TMT multiplex set 1, plex 1) in 21-day iNeurons derived from WT, *TMEM230*^*−/−*^ and *TMEM230*^X121W^ cells in biological triplicate (Supplementary Table 4). FAIMS, high-field asymmetric waveform ion mobility spectrometry. **j**, Violin plots (log_2_[fold change]) for the indicated cohorts of proteins of PNS from *TMEM230*^X121W^ and *TMEM230*^*−/−*^ (KO) iNeurons, relative to WT cells. Two-sided paired *t*-test; *P < 0.01; **P < 0.001; ***P < 0.0001 (*n* = 3 biologically independent replicates). For violin plots, the middle line corresponds to the median; the lower and upper lines correspond to the first and third quartiles, respectively. PM, plasma membrane. **k**, SynGO location and function enrichment analysis of proteins significantly regulated in Endo-IP from *TMEM230*^X121W^ iNeurons (Supplementary Table 4). The indicated categories were significantly enriched (−log_10_[*q* value]). SV, synaptic vesicle. Panel **i** adapted from ref. ^44^, CC BY 4.0; illustration of MS machine from NIAID NIH BioArt Source. Source data

**Extended Data Fig. 5:**
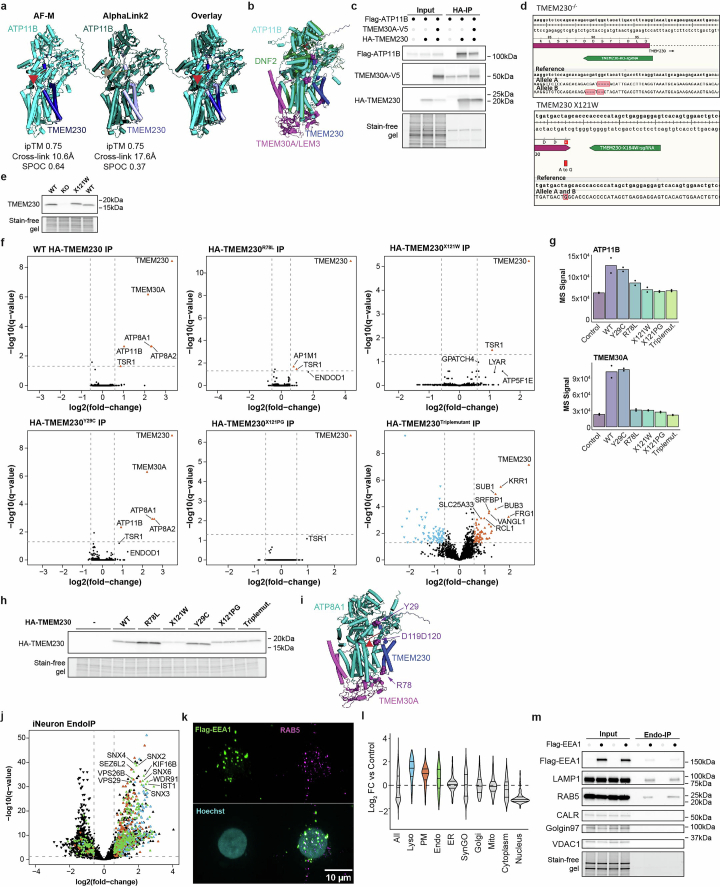
Interface variants disrupt interaction of TMEM230 with endosomal P4 lipid flippases ATP11B and ATP8A1/2. **a**, Individual and overlay AF-M and AlphaLink2 predictions for TMEM230 and ATP11B. AF-M: TMEM230 (light blue), ATP11B (cyan), cross-link (red line and arrowhead). AlphaLink2: TMEM230 (dark blue), ATP11B (teal), cross-link (wheat line and arrowhead). **b**, Overlay of yeast DNF1-LEM3 structure (PDB:7DRX) in the EP2 conformation with AF-M prediction for ATP11B-TMEM30A-TMEM230. **c**, Co-precipitation of Flag-ATP11B and TMEM30A-V5 with HA-TMEM230. The indicated plasmids were transfected into HEK293 cells and α-HA immunoprecipitates or input samples were immunoblotted for the indicated proteins. Black dots indicate proteins expressed in each sample. **d**, Sequence validation of TMEM230^-/-^ and TMEM230^X121W^ clones in H9^AAVS1-NGN2;Flag-EEA1^ cells (H9-Flag-EEA1), showing the location of the sgRNA used (green) and base pairs deleted to create an out of frame mutation and point mutation, respectively. **e**, Immunoblot of total cell lysates from the indicated H9-Flag-EEA1 cell lines probed with α-TMEM230. The X121W mutation adds a six-residue extension (WHPPHS), which can be detected as a band with slightly higher molecular weight. Stain-free gel was used to indicate equal loading of extracts. **f**, Volcano plots (log_2_FC relative to TMEM230^-/-^ cells) of TMEM230 immunoprecipitations in H9-TMEM230^-/-^ iNeurons with or without lentiviral expression of WT and interface variant HA-TMEM230 proteins. **g**, Mass spectrometry (MS) TMT reporter signal for ATP11B and TMEM30A in the indicated TMEM230 variant immunoprecipitation from iNeurons. Dots indicate individual biological replicates (n = 2, except n = 3 for Control given the limitation of the maximum number of TMT channels). **h**, Immunoblots of total cell extracts from TMEM230^-/-^ iNeurons transduced with lentiviruses expressing the indicated variants of HA-TMEM230 protein. Stain-free gel was used as loading control. **i**, AF-M prediction for a TMEM230-ATP8A1-TMEM30A complex (Y29, R78, and C-terminal D120-D121, purple space fill). The location of a cross-link between ATP8A1 and TMEM30A is indicated by the red line and arrowhead. ipTM = 0.74 for ATP8A1-TMEM230 prediction. **j**, Volcano plot for Endo-IP proteomic analysis from H9-Flag-EEA1 iNeurons (21 days) (n = 3 biologically independent replicates). Proteins annotated as endosomal (green), lysosomal (blue), or plasma membrane (PM, orange) are indicated. **k**, Immunofluorescence microscopy showing the colocalization of Flag-EEA1 (green) with RAB5 (magenta) in iNeurons from H9-Flag-EEA1 cells. **l**, Violin plot showing the fold-change enrichment (log_2_) of proteins from individual organelle compartments (color-coded as panel **j**) in Endo-IP samples from H9-Flag-EEA1 iNeurons (day 21). **m**, Immunoblots of Endo-IP or input samples (PNS) from H9-Flag-EEA1 iNeurons and untagged H9 control (21 days). Blots were probed with the indicated antibodies.

To validate interactions and structural predictions within EndoMAP.v1, we initially selected the TMEM230–ATP11B–TMEM30A complex given: strong structural prediction scores for TMEM230–ATP11B (AF-M SPOC = 0.64, ipTM = 0.75; Fig. 3a); clear co-migration in BN–MS (Fig. 3b); and a TMEM230–ATP11B crosslink satisfying distance constraints (Fig. 3a and Supplementary Table 2). The ATP11 proteins (A, B and C) are P4-type ATP-dependent enzymes that flip lipids from exofacial to cytosolic leaflets of a bilayer^33^, mainly the endolysosomal membrane for ATP11B (ref. ^10^). ATP11, as well as ATP8A1 and ATP8A2, interacts with TMEM30A and TMEM30B (also known as CDC50A and CDC50B)^34^, required for flippase trafficking from the endoplasmic reticulum to the Golgi apparatus^35,36^. The AF-M TMEM230–ATP11B–TMEM30A heterotrimer prediction closely matched previously reported ATP11–TMEM30 structures^33,34^ and predicted packing of the transmembrane and amino-terminal cytosolic segments of TMEM230 with TM1 and the cytosolic catalytic domain of ATP11B, respectively (Fig. 3a). The AlphaLink2 prediction for TMEM230–ATP11B exhibited a similar TMEM230–ATP11B interface with a slightly longer crosslink distance compared with that of AF-M (Fig. 3c and Extended Data Fig. 5a). The ATPase domain of ATP11B in the predicted heterotrimer approximates the EP2 conformation of the corresponding orthologous yeast DNF2 protein (Extended Data Fig. 5b). ATP11B interaction with TMEM230 and TMEM30A was confirmed reciprocally through co-IP in HEK293 cells^28^ (Fig. 3d and Extended Data Fig. 5c). These data identified TMEM230 as a subunit of the ATP11 family of lipid flippases and provided structural predictions of the complexes.

Several variants of unclear significance have been reported in TMEM230 (R78L, Y29C and two variants, X121W and X121PG, that cause six-residue carboxy-terminal extensions)^37–42^, which we found to map to the predicted TMEM230–ATP11B interface (Fig. 3a,e). TMEM230 R78 is located in proximity to D82 in TM1 of ATP11B, and the TMEM230 C terminus (D119–D120) is predicted to bind into a basic pocket of ATP11 (Fig. 3e), wherein TMEM230 variants causing C-terminal extension would be expected to sterically disrupt these interactions. To test the impact of these variants on ATP11B interactions and given the apparent role of ATP11B and TMEM230 in neuronal function^37,43^, we deleted *TMEM230* in human embryonic stem cells (H9 AAVS1-NGN2;Flag–EEA1, H9 Flag–EEA1; Extended Data Fig. 5d,e), and converted the cells to cortical-like induced neurons (iNeurons) using the NGN2 driver. Wild-type (WT) TMEM230 co-immunoprecipitated with ATP11B, ATP8A1, ATP8A2 and TMEM30A, compared to *TMEM230*^*−/−*^ iNeurons as control (Fig. 3f and Supplementary Table 4). By contrast, TMEM230 interactions with TMEM30A, ATP11B, ATP8A1 and ATP8A2 were lost in R78L and both stop codon variants as determined by tandem mass tagging (TMT)-MS (Fig. 3f,g, Extended Data Fig. 5f–h and Supplementary Table 4). The Y29C variant^37,40,41^ was without effect. Loss of interaction of TMEM230(Y29C/R78L/X121W) was also validated in HEK293 cells (Fig. 3h). AF-M predicts TMEM230 interaction with ATP8A1 and A2 (ipTM > 0.73) in a manner very similar to that seen with ATP11 isoforms (Extended Data Fig. 5i), consistent with loss of interaction in the context of interface variants (Fig. 3g).

**Extended Data Fig. 6:**
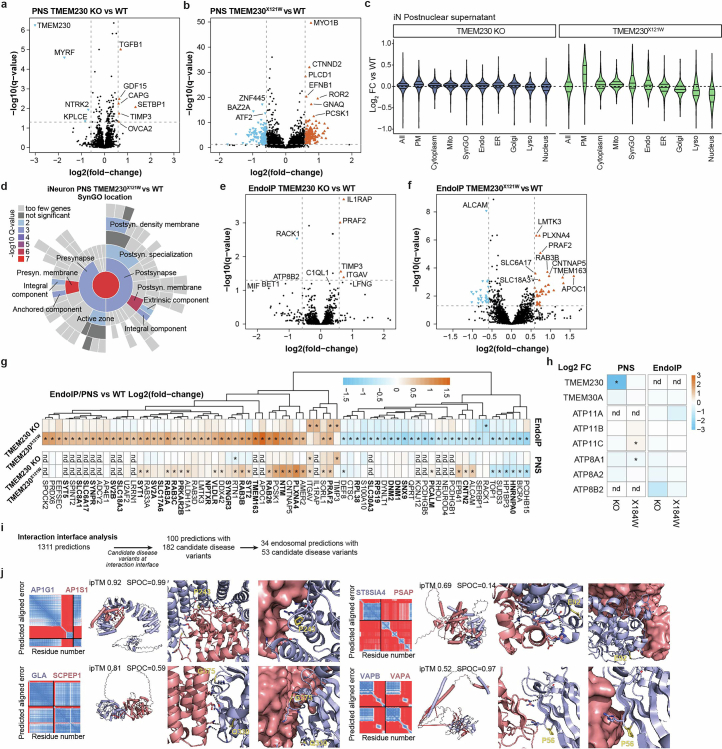
Proteomic profiling of postnuclear supernatant (PNS) and Endo-IP from TMEM230 mutant iNeurons. **a,b**, Volcano plots of PNS proteomic analysis from TMEM230^-/-^ (panel **a**) and TMEM230^X121W^ (panel **b**) iNeurons compared to WT (day 21) (n = 3 biologically independent replicates). **c**, Violin plot showing the fold-change enrichment (log_2_) of proteins from individual organelle compartments in PNS from TMEM230^-/-^ and TMEM230^X121W^ iNeurons compared to WT (day 21). **d**, SynGO location enrichment analysis of proteins significantly regulated in PNS from TMEM230^X121W^ iNeurons (Supplementary Table 4). The indicated categories were significantly enriched (−log_10_q-value). **e,f**, Volcano plots of Endo-IP proteomic analysis from TMEM230^-/-^ (panel **e**) and TMEM230^X121W^ (panel **f**) iNeurons compared to WT (day 21) (n = 3 biologically independent replicates). **g**, Heatmap showing the abundance fold-changes (log_2_) for all significantly regulated proteins in Endo-IPs from TMEM230^-/-^ or TMEM230^X121W^ iNeurons (21 day) compared to WT. Synaptic proteins annotated in SynGO (see **METHODS**) are indicated in bold. Asterisks indicate significantly regulated proteins (q-value < 0.05 and fold-change > 1.5). Abundance fold-changes in PNS are also indicated, except for proteins not detected (nd). **h**, Heatmap for the abundance fold-changes (log_2_FC) of selected proteins in PNS and Endo-IPs from TMEM230^-/-^ or TMEM230^X121W^ iNeurons (21 day) compared to WT. Asterisks indicate significantly regulated proteins (q-value < 0.05 and fold-change > 1.5) and nd for proteins not detected. **i**, Summary of pairwise AF-M predictions harboring candidate disease variants within 2 amino acids of the interface for endosomal and non-endosomal proteins. **j**, Candidate disease variants at the interaction interface of pairwise protein AF-M predictions. Predicted aligned error plots (left), predicted structures with ipTMs (center left, interprotein DSSO cross-links indicated by red lines) and close-up view of disease variant residues (yellow) at the interaction interface (center right, and right; dotted lines indicate predicted hydrogen bonds). Source data

To examine the effect of TMEM230 variants on early endosomes, we analysed Endo-IP and postnuclear supernatant (PNS) proteomes in *TMEM230*^*−/−*^ and *TMEM230*^X121W^ iNeurons^44^ (Fig. 3i, Extended Data Fig. 5d,e,j–m and Supplementary Table 4). For PNS proteomes, the abundances of plasma membrane and synaptic proteins based on the SynGO database were selectively elevated in *TMEM230*^X121W^ iNeurons relative to WT cells, whereas minimal abundance changes were found in *TMEM230*^*−/−*^ iNeurons (Fig. 3j, Extended Data Fig. 6a–d and Supplementary Table 4). For endosomal proteomes, we found a lower number of proteins whose abundance was altered compared to PNS (Extended Data Fig. 6e–g), and involved the synaptic vesicle cycle and its membrane components (Fig. 3k and Supplementary Table 4) in *TMEM230*^X121W^ iNeuron endosomes. Proteins whose abundance was increased on early endosomes of *TMEM230*^X121W^ iNeurons included several RAB proteins (for example, RAB3A and RAB3B) and endocytic cargo (for example, SORL1), whereas levels of DNM1 and DNM2 (involved in endosomal vesicle budding) were decreased (Extended Data Fig. 6g). The abundance of ATP8, ATP11 and TMEM30A was unaffected in total or endosomal proteomes (Extended Data Fig. 6h). Thus, reported variants in TMEM230 (refs. ^40–42^) disrupt interactions with multiple lipid flippases and alter the abundance of endosomal and plasma membrane proteins in iNeurons. Following an analogous approach, we examined candidate disease variants at interaction interfaces for all pairwise predictions in our dataset and identified 53 candidate disease variants nearby 34 predicted interfaces (Extended Data Fig. 6i,j, Supplementary Text and Supplementary Table 3).

## New subunits of CLCN3 and CLCN5 complexes

**Fig. 4:**
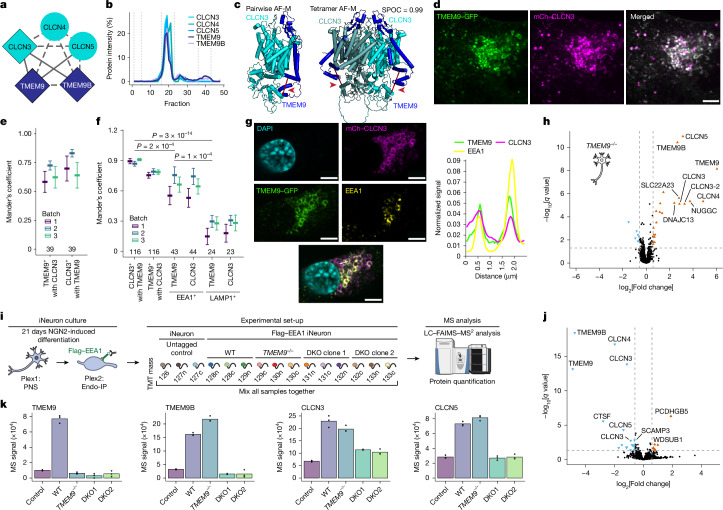
TMEM9 and TMEM9B are core subunits of endosomal CLCN3, CLCN4 and CLCN5 Cl^−^–H^+^ antiporters. **a**, EndoMAP.v1 interactions for CLCN3, CLCN4, CLCN5, TMEM9 and TMEM9B. Diamonds and circular nodes represent endosomal and other proteins, respectively. Solid and dashed edges represent interactions identified by at least one crosslink or only co-fractionation, respectively. **b**, BN–MS profiling for CLCN3, CLCN4, CLCN5, TMEM9 and TMEM9B. **c**, AF-M predictions for CLCN3–TMEM9 pair and heterotetramer. The locations of DSSO crosslinks are indicated with the red line and arrowhead. **d**,**e**, Co-localization analysis of TMEM9–GFP and mCh–CLCN3 in SUM159 cells by live-cell imaging. Mander’s coefficients of GFP and mCh puncta are shown in **e** (*n* = 39 in 3 independent replicates, mean ± s.e.m.), with an example of a cell shown in **d**. **f**, Mander’s coefficient analysis of co-localization between TMEM9–GFP, mCh–CLCN3, anti-EEA1 and anti-LAMP1 in fixed SUM59 cells as determined by immunofluorescence. The number of fields of view across three biological replicates is indicated (mean ± s.e.m.) and *P* values from linear mixed-effects model analysis of variance. **g**, Example of TMEM9–GFP, mCh–CLCN3 and anti-EEA1 staining in a cell expressing high levels of CLCN3 (left panels), which promotes the formation of swollen endolysosomes. Traces of the white line in the bottom panel show the overlap of the three proteins in the limiting membrane of endosomes (right panel). **h**, Volcano plot showing the proteomic analysis of anti-HA IPs from *TMEM9*^*−/−*^ iNeurons with or without lentiviral expression of TMEM9–HA (*n* = 4 biologically independent replicates). **i**, Schematic of experimental design for proteomic analysis of early endosomes and PNS in 21-day iNeurons derived from WT cells, *TMEM9*^*−/−*^ cells and two different clones of *TMEM9*^*−/−*^*TMEM9B*^*−/−*^ (DKO) cells in biological triplicate (Supplementary Table 5). **j**, Volcano plot showing the proteomic analysis of Endo-IPs from *TMEM9*^*−/−*^*TMEM9B*^*−/−*^ (DKO clone 2) versus WT iNeurons (day 21; *n* = 3 biologically independent replicates). CTSF, cathepsin F. **k**, TMT reporter signal intensity for CLCN3, CLCN5, TMEM9 and TMEM9B in Endo-IPs from iNeurons with the indicated genotypes (*n* = 3 biologically independent replicates). DKO1, *TMEM9*^*−/−*^*TMEM9B*^*−/−*^ (clone 1); DKO2, *TMEM9*^*−/−*^*TMEM9B*^*−/−*^ (clone 2). Scale bars (**d** and **g**), 5µm. Panel **i** adapted from ref. ^44^, CC BY 4.0; illustration of MS machine from NIAID NIH BioArt Source. Source data

**Extended Data Fig. 7:**
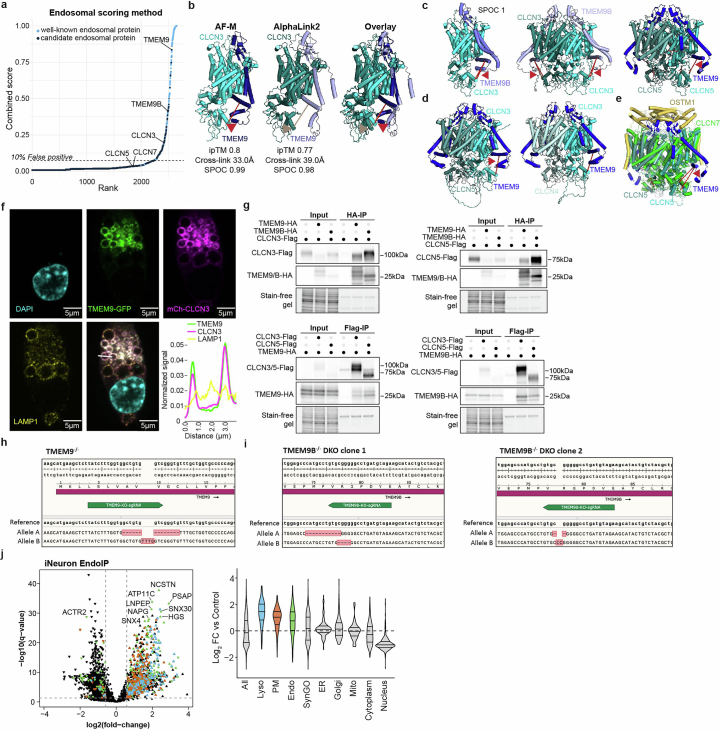
TMEM9/9B are core subunits of endosomal CLCN3/4/5 Cl^−^-H^+^ antiporters. **a**, Endosomal score and rank of TMEM9/9B and CLCN3/5/7, with higher values corresponding to endosomal proteins. **b**, Individual and overlay AF-M and AlphaLink2 predictions for CLCN3-TMEM9. AF-M: TMEM9 (dark blue), CLCN3 (cyan), cross-link (red bar and arrowhead). AlphaLink2: TMEM9 (light blue), CLCN3 (teal), cross-link (wheat bar and arrowhead). **c**,**d** AF-M predictions for CLCN3-TMEM9B and selected CLCN-TMEM9/9B heterotetramers. The locations of DSSO cross-links are indicated with the red line and arrowhead. The location of variants found in CLCN5 in Dent’s Disease retrieved from UniProt are shown in red (right, panel **c**). **e**, Overlay of the CLCN5-TMEM9 heterotetramer prediction with the CLCN7-OSTM1 heterotetramer structure (PDB: 7JM7). **f**, Example of TMEM9-GFP, mCh-CLCN3, and α-LAMP1 staining in a cell expressing high levels of CLCN3, which promotes the formation of swollen endolysosomes. Line traces show the overlap of the 3 proteins in the limiting membrane of endolysosomes (bottom right panel). **g**, Co-precipitation of CLCN3/5-Flag and TMEM9/9B-HA. The indicated plasmids were transfected into HEK293 cells and α-HA or α-Flag immunoprecipitations or input samples were immunoblotted with the indicated antibodies. Loading controls as stain-free gels are shown. Black dots indicate proteins expressed in each sample. **h,i**, Sequence validation of H9 TMEM9^-/-^ cells, showing the location of the sgRNA used (green) to create frameshift mutations in TMEM9 (panel **h**) and subsequently, used the indicated sgRNA (green) to create frameshift mutations in TMEM9B (panel **i**). **j**, (Left) Volcano plot for Endo-IP proteomic analysis from H9-Flag-EEA1 iNeurons (n = 3 biologically independent replicates). (Right) Violin plot showing the fold-change enrichment (log_2_) of proteins from individual organelle compartments in Endo-IP from H9-Flag-EEA1 iNeurons. Proteins annotated as endosomal (green), lysosomal (blue), or plasma membrane (PM, orange) are indicated. Source data

High luminal chloride (Cl^−^) ion concentrations activate several endolysosomal enzymes and have been proposed to provide counterions to support the V-ATPase-generated H^+^ gradient^45–47^. The Cl^−^–H^+^ antiporters CLCN3, CLCN4 and CLCN5 are proposed to function primarily in endosomes, whereas a heterotetrameric complex composed of CLCN7 α-subunits and OSTM1 β-subunits functions primarily in lysosomes^45,48^. CLCN3 variants are implicated in intellectual disability^49^, and CLCN3 deficiency leads to neurodegeneration in mice^50^. EndoMAP.v1 identified crosslinks between CLCN3 or CLCN5 and TMEM9 or TMEM9B (Fig. 4a), a strong enrichment of TMEM9 and TMEM9B in early endosomes^18,19^ (Extended Data Fig. 7a) and co-migration of CLCN3, CLCN4, CLCN5, TMEM9 and TMEM9B in BN–MS (Fig. 4b). Pairwise AF-M and AlphaLink2 predicted interaction of CLCN3 or CLCN5 with two transmembrane segments from TMEM9 or TMEM9B (SPOC > 0.97), including compatible crosslink distances for AF-M (Fig. 4c and Extended Data Fig. 7b). As CLCN proteins form homodimers and heterodimers^51^, we examined tetrameric predictions of CLCN3 or CLCN5 with TMEM9 or TMEM9B that had the expected antiporter dimer interface, with two molecules of TMEM9 (or TMEM9B) compatible with the crosslink distance constraint (Fig. 4a,c and Extended Data Fig. 7c,d). The relative orientation of the two transmembrane segments in TMEM9 was distinct from that of the single transmembrane segment in OSTM1 (Extended Data Fig. 7e). Additionally, the two β-β-α-α-α-β folds of the two TMEM9 molecules occupy a similar location to the helical luminal ‘cap’ domain of OSTM1, but with a distinct conformation (Extended Data Fig. 7d,e).

Several experiments further validated interaction of TMEM9 with CLCN3 and CLCN5 in early endosomes. First, TMEM9–GFP and mCherry (mCh)–CLCN3 co-localized in vesicles in live (Mander’s coefficient ≈0.64–0.72; Fig. 4d,e and Supplementary Video 1) and fixed cells, in which extensive co-localization with EEA1^+^ vesicles compared to LAMP1^+^ vesicles was observed (Mander’s coefficient ≈0.65 and ≈0.25, respectively; Fig. 4f). Second, TMEM9–GFP tracked with expected swollen endosomes in mCh–CLCN3-overexpressing cells^52^ (Fig. 4g and Extended Data Fig. 7f). Third, Flag–CLCN3 or Flag–CLCN5 reciprocally associated with HA-tagged TMEM9 and TMEM9B in HEK293 cells by co-IP (Extended Data Fig. 7g).

**Extended Data Fig. 8:**
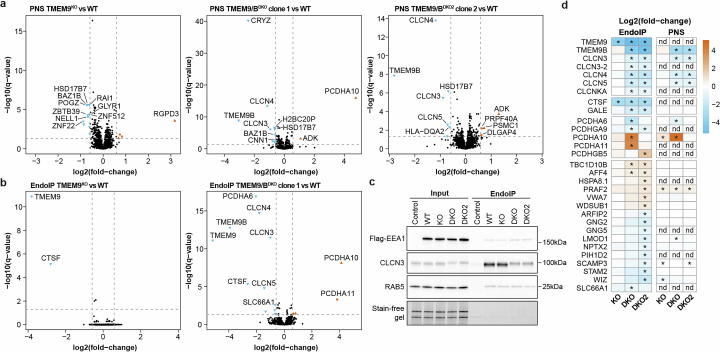
Proteomic profiling of postnuclear supernatant (PNS) and Endo-IP from TMEM9^-/-^ and TMEM9/9B^DKO^ iNeurons. **a**, Volcano plots for PNS (post-nuclear supernatants) proteomic analysis from TMEM9^-/-^ and two clones of TMEM9/9B^DKO^ iNeurons (day 21) compared to WT (n = 3 biologically independent replicates). **b**, Volcano plots for Endo-IP proteomic analysis from TMEM9^-/-^ and one clone of TMEM9/9B^DKO^ iNeurons compared to WT (n = 3 biologically independent replicates). **c**, Immunoblots of input and Endo-IP samples from the experiment outlined in Fig. 4i. Blots were probed with the indicated antibodies. **d**, Heatmap showing the abundance fold-changes (log_2_) for all significantly regulated proteins in Endo-IPs from TMEM9^-/-^ and TMEM9/9B^DKO^ iNeurons (day 21) compared to WT. Asterisks indicate significantly regulated proteins (q-value < 0.05 and fold-change > 1.5). Abundance fold-changes in PNS are also indicated, except for proteins not detected (nd).

To systematically examine TMEM9 interaction partners, we created *TMEM9*^*−/−*^ embryonic stem cells (Extended Data Fig. 7h) and expressed TMEM9–HA in biological quadruplicate day-21 iNeurons before TMT-based IP–MS (Fig. 4h and Supplementary Table 5). CLCN3, CLCN4 and CLCN5, as well as TMEM9B, were all highly enriched in anti-HA immunoprecipitates, demonstrating specific interaction of TMEM9 with multiple CLCNs and TMEM9B-containing heterotetramers^28^. Finally, we performed PNS and Endo-IP proteomics for WT iNeurons, *TMEM9*^*−/−*^ iNeurons and two different clones of iNeurons in which both *TMEM9* and *TMEM9B* were knocked out (*TMEM9*^*−/−*^*TMEM9B*^*−/−*^) (Fig. 4i, Extended Data Fig. 7i,j and Supplementary Table 5). Early endosome and PNS proteomics revealed a selective reduction in the abundance of CLCN3, CLCN4 and CLCN5 together with TMEM9 and TMEM9B, as well as CLCNKA and cathepsin F (Fig. 4j,k and Extended Data Fig. 8a–d), with reduced CLCN3 levels in *TMEM9*^*−/−*^*TMEM9B*^*−/−*^ confirmed by immunoblotting (Extended Data Fig. 8c). The interaction, co-localization and selective dependency between the protein levels of CLCN and those of TMEM9 and TMEM9B in iNeurons reveal TMEM9 and TMEM9B as core components of CLCN antiporter complexes in endosomes and suggests a role in complex stability and/or endosomal trafficking, consistent with findings reported while this manuscript was under revision^53^.

## From EndoMAP.v1 to high-order complexes

**Fig. 5:**
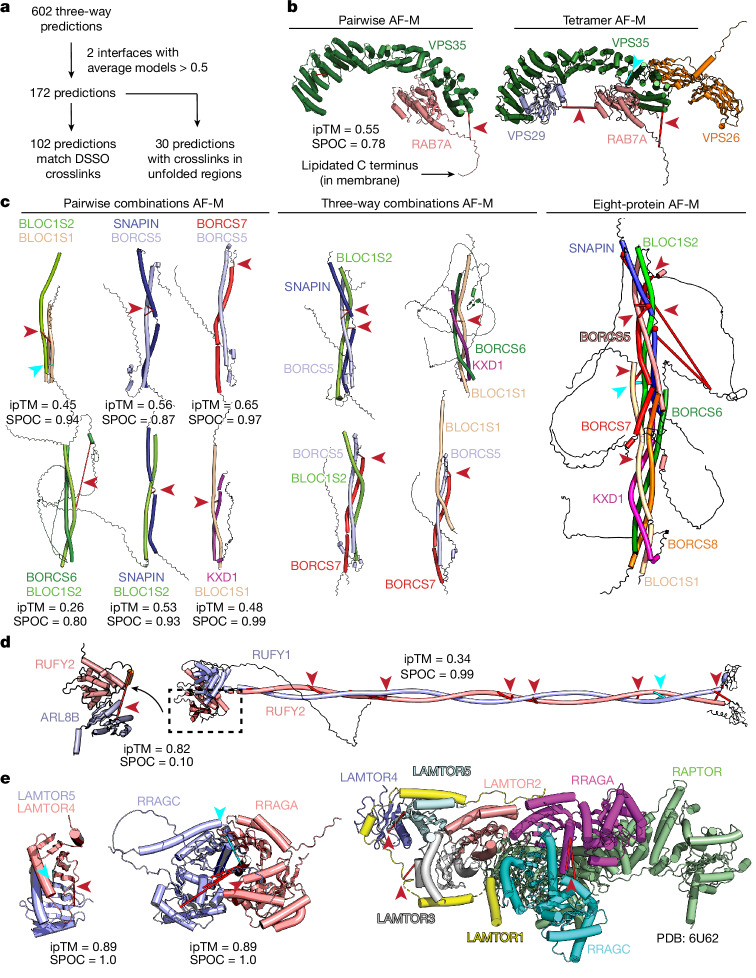
Towards a structural proteomic landscape for early endosomes. **a**, Systematic AF-M structural predictions for three-way clique assemblies within EndoMAP.v1 and match with the crosslinker distance constraints. **b**, Pairwise AF-M prediction for VPS35–RAB7A (left) and tetramer prediction for retromer–RAB7A complex (right) and associated crosslinks from EndoMAP.v1. ipTM and SPOC scores for pairwise combination are shown. **c**, AF-M structural predictions and interprotein crosslinks within the BORC endolysosomal positioning complex. Pairwise AF-M predictions (left), three-way clique predictions (middle) and eight-protein predictions (right) are shown along with associated interprotein crosslinks. ipTM and SPOC scores for pairwise combinations are indicated. **d**, Pairwise AF-M predictions and associated crosslinks for a RUFY1–RUFY2 heterodimer (right) and for interaction of the RUFY2 N-terminal helical domain with ARL8B (left). **e**, Pairwise AF-M predictions and associated crosslinks for LAMTOR4 and LAMTOR5 (left), RRAGA and RRAGC (middle), and crosslinks mapped onto the ragulator structure (PDB 6U62)^60^ (right). DSSO crosslinks (red) and DHSO or DMTMM crosslinks (cyan) are indicated with lines and arrowheads.

**Extended Data Fig. 9:**
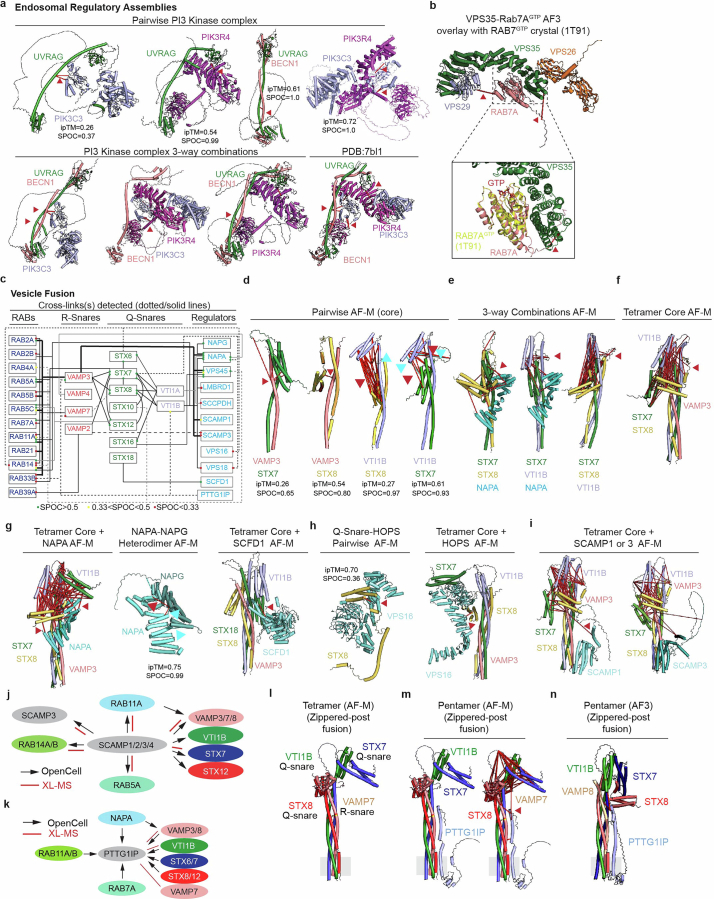
3-way Clique and higher order AF-M predictions reveal extensive SNARE interactions and assemblies. **a**, Pairwise (top) and 3-way clique (bottom) AF-M predictions and associated DSSO cross-links for components of the Class II PI3K complex. Identified cross-links are also mapped onto the cryo-EM structure of the PI3K complex (PDB:7bl1) (lower right). **b**, Overlay of AF3 predictions of a VPS29-VPS35-VPS26A-RAB7A^GTP^ complex and associated DSSO cross-links with a RAB7A^GTP^ crystal structure (PDB:1T91). **c**, Summary of cross-link and AF-M predictions for SNARE components and their interactors. R-SNARE, Q-SNARE, known and candidate regulators and RAB proteins found with cross-links within EndoMAP.v1 are shown. Lines indicate one or more cross-links and are shown in distinct forms to facilitate visualization of connections. Colored dots indicate SPOC score for each AF-M pairwise prediction. **d**, Cross-links and pairwise AF-M predictions for “core” SNARE components VAMP3, STX7, STX8, and VTI1B. ipTM and SPOC scores are indicated for pairwise combinations. **e**, Examples of a subset of 3-way clique predictions and associated cross-links involving core SNARE components as well as NAPA. **f**, Core SNARE AF-M predictions and associated cross-links. The prediction resembles a post-vesical fusion-like conformation. **g**, AF-M predictions and associated cross-links for SNARE association with soluble fusions factors. **h**, Predicted interactions and cross-links for association of VPS16 with either STX8 or STX8 in the core SNARE complex. **i**, Core SNARE assembly predictions and associated cross-links with candidate interactors SCAMP1 and SCFD1. **j**, Summary of physical interactions involving SCAMP proteins in OpenCell^119^ and cross-links identified in our study. Intraprotein cross-links are not shown (see Supplementary Table 2). **k**, Summary of physical interactions involving PTTG1IP proteins in OpenCell^119^ and cross-links identified in our study. **l**, AF-M prediction for tetrameric SNARE complex composed of VTI1B, STX7, STX8, and VAMP7. **m**, Pentameric prediction for VTI1B, STX7, STX8, and VAMP7 together with PTTG1IP. Grey rectangles represent the transmembrane section of the complex. Left, cross-links not shown; Right, cross-links shown. **n**, Pentameric AF3 prediction for VTI1B, STX7, STX8, and VAMP8 together with PTTG1IP. DSSO and DHSO/DMTMM interprotein cross-links are indicated with red and cyan lines and arrowheads, respectively.

To expand the endosomal structural interactome beyond protein pairs, we identified and performed AF-M predictions on all 625 three-way cliques (combinations of three proteins interacting with each other) within EndoMAP.v1, with each clique requiring at least one crosslink-supported interaction. This approach yielded 172 predictions containing ≥2 well-predicted interaction interfaces (interface average models >0.5) within each clique (Fig. 5a and Supplementary Table 6). A total of 59% of these predictions matched interprotein crosslink constraints and an additional 17% involved crosslinks within unstructured regions. Predictions for three-way cliques represent a methodological approach for interrogation of iterative predictions and assessment of crosslink data, as well as serving as an intermediate step in the generation of hypotheses for higher-order complexes, and do not necessarily represent models for endogenous complexes. Illustrating the potential use of the three-way clique approach for analysis of complexes with >3 subunits, pairwise and three-way clique predictions for combinations of endosomal class II PI3 kinase (UVRAG, BECN1, PIK3C3 and PIK3R4) subunits recapitulate key intersubunit interactions across the resolved complex structure^27^, with valid crosslink distances for each pair and three-way assembly (Fig. 1i and Extended Data Fig. 9a).

Several multiprotein complex predictions were generated for core endolysosomal regulators with previously defined components and stoichiometry but lacking structural information through the three-way clique AF-M approach. First, we identified a clique containing the retromer subunits VPS35 and VPS29, as well as the endosomal GTPase RAB7A. Here, RAB7A directly binds to the concave surface of the VPS35 α-solenoid fold (SPOC = 0.78), supported by both DSSO and DHSO or DMTMM crosslinks, in a manner compatible with simultaneous binding of VPS26A and VPS29 to VPS35, thus the assembled retromer complex^14^ (Fig. 5b). The AlphaFold3 (ref. ^54^) prediction for the complex between VPS35 and GTP-bound RAB7 closely matches the crystal structure of GTP-bound RAB7A and provides a plausible structural mechanism for the previously reported ability of GTP-bound RAB7A to recruit retromer to endosomes^55^ (Extended Data Fig. 9b). Similarly, pairwise and three-way clique combinations facilitate AF-M-driven assembly of the eight subunits forming the kinesin-associated endolysosomal positioning BORC complex, for which structural data are lacking (Fig. 5c). The predicted four-helix bundle with eight interdigitated subunits is supported by multiple DSSO and DHSO or DMTMM crosslinks and is consistent with known stoichiometry^22^ (Fig. 5c). Acting in opposition to BORC for retrograde endosome trafficking are RUFY (RUN and FYVE domain) proteins, which link ARL8-tethered endolysosomes with dynein motors. Multiple DSSO and DHSO or DMTMM crosslinks between RUFY1, RUFY2 and/or ARL8B validate an extended RUFY1–RUFY2 coil–coil structure thought to be dimeric^56^ (SPOC = 0.99), with ARL8B binding the RUN domain (ipTM = 0.82; Fig. 5d).

**Extended Data Fig. 10:**
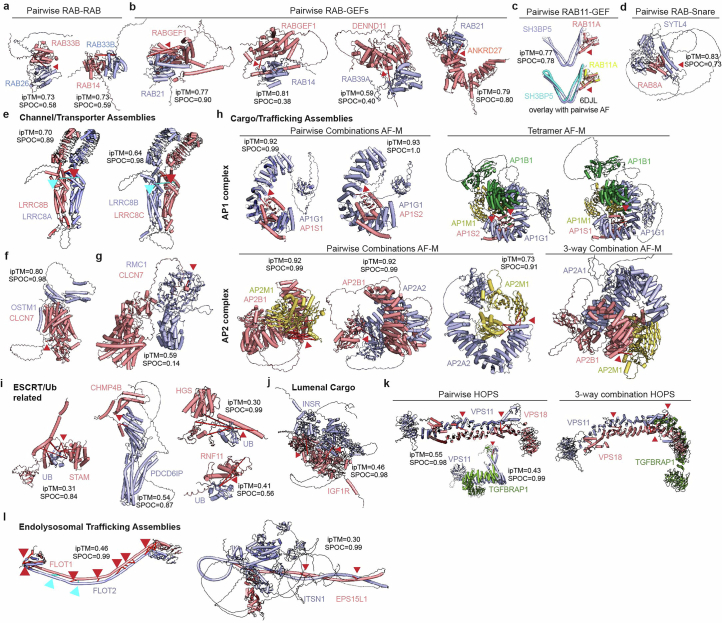
Endosomal Regulatory Proteins, Channels, Cargo, and Trafficking Complex AF-M Predictions. **a**, Pairwise AF-M predictions and associated cross-links for two pairs of RABs with high scoring predictions. **b,c**, Pairwise AF-M predictions and associated cross-links for selected RABGEF complexes present in EndoMAP.v1 (panel **b**), and for a RAB11A-SH3BP5 complex (panel **c**) overlayed with a previously determined structure of the complex (PDB:6DJL). **d**, Pairwise AF-M prediction and associated cross-links for a RAB8A-SYTL4 (synaptotagmin-like) Snare complex. **e-g**, AF-M predictions and associated cross-links for selected channel/transporter assemblies in EndoMAP.v1. LRRC8 proteins (panel **e**) form hexamers and are components of volume regulated anion channels important for cell volume homeostasis. OSTM1-CLCN7 (panel **f**) is an endolysosomal voltage-gated channel mediating exchange of chloride against protons and is known to form a heterotetramer. CLCN7 was found cross-linked to RMC1 (panel **g**), a subunit of the CCZ1-MON1 GEF for RAB7 on endolysosomes. **h**, Pairwise AF-M predictions for cross-link containing AP1 components AP1G1 and either AP1S1 or AP1S2 (left) and tetramer AF-M prediction for AP1G1-AP1B1-AP1M1 and either AP1S1 or AP1S2 (upper panel). Pairwise and 3-way clique predictions for AP2 components AP2M1, AP2B1, or AP2A2 (lower panel). **i**, Pairwise predictions and associated cross-links for ESCRT and ubiquitin (Ub)-related modules within EndoMAP.v1. **j**, Pairwise predictions and associated cross-links for INSR and IGF1R. **k**, Pairwise AF-M predictions and associated cross-links for selected HOPS complex components (left) and a 3-way clique prediction (right) that maintains compatible cross-link distances. **l**, Pairwise predictions and associated cross-links for the FLOT1/2 complex that participates as a scaffolding protein within caveolar membranes, and the ITSN1-EPS15L complex that links endosomal membrane trafficking with actin assembly machinery. For all panels, DSSO and DHSO/DMTMM interprotein cross-links are indicated with red and cyan lines and arrowheads, respectively. Intraprotein cross-links are not shown (see Supplementary Table 2).

SNARE components facilitate endolysosomal vesicle fusion and maturation. Our data allowed the construction of an extensive structurally informed network of R- and Q-SNARE components in combination with regulatory RAB GTPases, tethering components, disassembly machinery and including new candidate SNARE interaction partners (SCAMP1, SCAMP3 and PTTG1IP) supported by crosslinking and PPI data (Extended Data Fig. 9c–n and Supplementary Text). Additional predictions allowed us to compile models for complexes linked with several endosomal functions, including RAB–GEF (Extended Data Fig. 10a–d), channel–transporter (Extended Data Fig. 10e–g), adaptor protein (AP; Extended Data Fig. 10h), ESCRT–ubiquitin (Extended Data Fig. 10i), luminal cargo (Extended Data Fig. 10j), HOPS (Extended Data Fig. 10k) and cargo trafficking assemblies (Extended Data Fig. 10l), with experimental validation in purified endosomes through DSSO and DHSO or DMTMM crosslinks.

## V-ATPase as an interaction hub

**Extended Data Fig. 11:**
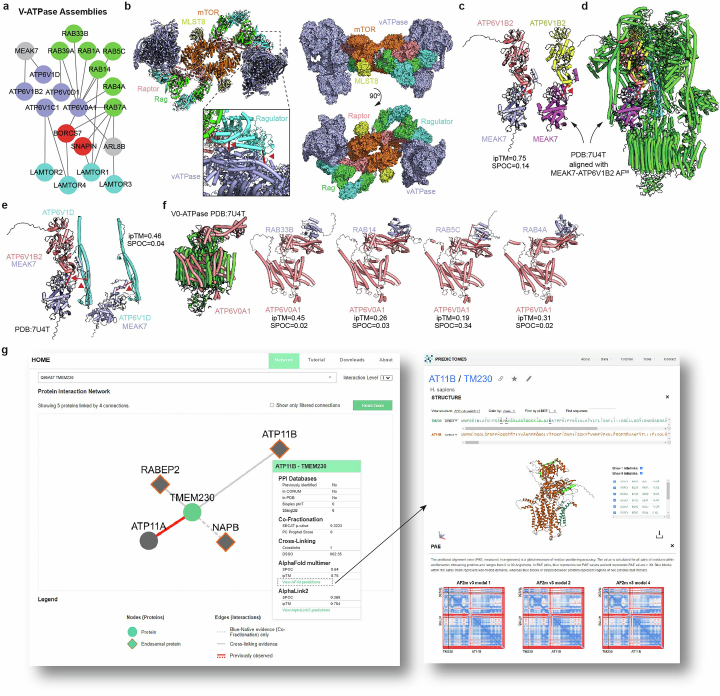
V-ATPase as an interaction hub. **a**, DSSO cross-links identified between components of the V-ATPase (purple), the BORC complex (red), the LAMTOR complex (blue) and RAB proteins (green). **b**, Hypothetical model for association of MTOR-Ragulator complex (PDB:7UXH)^58^ with V-ATPase^ADP^ (PDB:6WM2)^59^ based on two DSSO cross-links between LAMTOR2 and LAMTOR4 with ATP6V1C1 (red lines). Docking model was generated using HADDOCK (see **METHODS**). **c**, Pairwise AF-M prediction and associated cross-link for MEAK7 and ATP6V1B2 (left) is compared with the MEAK7-ATP6V1B2 sub-complex from PDB:7U4T (right)^171^. **d**, MEAK7-V1-ATPase (PDB:7U4T)^171^ together with overlay of AF-M prediction for MEAK7-ATP6V1B2. Cross-links between MEAK7 and either ATP6V1B2 or ATP6V1D are shown by red lines^172^. **e**, MEAK7-ATP6V1B2 AF-M prediction modeled on PDB:7U4T and identified cross-links with ATP6V1D (red arrowhead) is shown on the left. MEAK7-ATP6V1D AF-M prediction and associated cross-link is shown on the right. **f**, Endosomal RABs cross-link with the ATPV0A1 subunit of the V0-ATPase. The structure of the V0-ATPase complex, with ATP6V0A1 shown in salmon, is presented on the far left. The AF-M predictions for 4 endosomal RABs and the detected cross-links are also shown. Intraprotein cross-links are not shown (see Supplementary Table 2). **g**, Screenshot from our EndoMAP.v1 website and AF-M prediction viewer at https://endomap.hms.harvard.edu/. Left panel shows the output of a search for TMEM230. Right panel shows the output of AF-M prediction for TMEM230-ATP11B interaction, together with predicted alignment error plots.

Among the most extensively crosslinked complex was the V-ATPase (Fig. 1h and Extended Data Fig. 11a), which pumps protons into the endolysosomal lumen to maintain an acidic pH. V-ATPase can co-IP ragulator complexes (a five-subunit LAMTOR complex together with RRAGA and RRAGC or RRAGB and RRAGD GTPase), which bind and regulate MTOR kinase on the endolysosomal membrane^13,57^. We detected multiple DSSO and DHSO or DMTMM crosslinks between ragulator subunits, consistent with its known structure and pairwise AF-M predictions (Fig. 5e). We detected crosslinks between LAMTOR2 or LAMTOR4 and the ATP6V1C1 subunit of V-ATPase, suggesting that LAMTOR comes into close contact with V-ATPase. Using the crosslinked Lys residues as a guide for hypothesis generation, we developed a hypothetical docking model of a previously reported symmetrically dimeric ragulator–MTORC1 complex coupled onto two fully assembled V-ATPase complexes, forming a V-ATPase-MTORC1 ‘super assembly’ (Extended Data Fig. 11b). This hypothetical model illustrates an orientation of V-ATPase interacting with MTORC1 complexes compatible with crosslinking data and the proposed organelle membrane topology for MTORC1–ragulator^58^, highlighting how our approach may capture contacts between large dynamic protein complexes and support the design of further experiments required to validate these hypotheses. Additional crosslinks and predictions suggest an extensive network of interactions linking V-ATPase and LAMTOR complexes with lysosomal positioning BORC, endosomal RAB and V-ATPase regulatory TLDc domain-containing proteins, as detailed in Supplementary Text (Extended Data Fig. 11a,c–f and Supplementary Table 3).

## Discussion

By combining protein interactions with crosslink-supported structural predictions, EndoMAP.v1 provides a framework for understanding the EEA1^+^ endosomal structural interactome. EndoMAP.v1 contains 4,282 interactions based on XL–MS and BN–MS with 229 structural predictions for endosomal interactions without previous structural information. This landscape can be explored through an interactive viewer containing all structural predictions, interactions and experimental data (https://endomap.hms.harvard.edu/; Extended Data Fig. 11g). We demonstrated how EndoMAP.v1 can be used to identify new core subunits of membrane protein complexes, as in the case of TMEM230, TMEM9 and TMEM9B. Moreover, we showed how XL–MS can provide experimental support for large-scale hypothesis-generating structural predictions in the context of an organelle, in which weak protein interactions may be facilitated through membrane tethering. Future studies will further expand on and address the limitations of this work, such as inclusion of additional endosome populations, improving the coverage of integral membrane proteins, assessing complex stoichiometries when such information is lacking, and addressing the challenge of biochemical and structural validation of proposed hypothetical models at scale (Supplementary Text). Finally, the pipeline described here serves as a roadmap for analogous efforts with other organelles and for understanding the diversity of organellar proteomes and interactions in diverse cell types.

## Methods

### Reagents

The following chemicals and reagents were used: Dounce homogenizer (DWK Life Sciences, 885302-0002); Pierce anti-HA magnetic beads (Thermo Scientific, 88837); Pierce anti-Flag magnetic agarose (Thermo Scientific, A36797); anti-Flag M2 magnetic beads (Sigma Millipore, M8823); Pierce protein A/G magnetic beads (Thermo Scientific, 88802); IGEPAL CA-630 (Sigma-Aldrich, I8896); S-Trap micro columns (Protifi, C02-micro-80); triethylammonium bicarbonate (TEAB) buffer (Sigma-Aldrich, T7408); sodium dodecyl sulfate (SDS; Bio-Rad, 1610302); DSSO (Thermo Scientific, A33545); DHSO (CF Plus Chemicals, PCL042); DMTMM (Sigma-Aldrich, 74104); *n*-dodecyl β-d-maltoside (DDM, Gold Biotechnologies, DDM5); NativeMark protein standard (Invitrogen, LC0725); NativePAGE 4–6% gels (Invitrogen, BN1002BOX); MultiScreen filter plates (Sigma Millipore, MSHVN4510); TMTpro 16plex set (Thermo Fisher Scientific, A44520); protease inhibitor cocktail (Roche, 4906845001); tris(2-carboxyethyl)phosphine (TCEP; Gold Biotechnology, 51805-45-9); 2-chloroacetamide (Sigma-Aldrich, C0267); *S*-methyl thiomethanesulfonate (MMTS; Sigma-Aldrich, 208795); trypsin (Promega, V511C); Lys-C (Wako Chemicals, 129-02541); hydroxylamine solution (Sigma-Aldrich, 438227); Sep-Pak C18 and C8 50 mg cartridge (Waters, WAT054955 and WAT054965); high-pH reversed-phase peptide fractionation kit (Thermo Scientific, 84868); Bio-Rad protein assay dye (Bio-Rad, 5000006); 3-[4-(2-hydroxyethyl)-1-piperazine]propanesulfonic acid (Thermo Scientific, J61296AE); Empore SPE discs C18 (Sigma Millipore, 66883-U); Gateway LR Clonase II enzyme mix (Thermo Scientific, 11791020); NEBNext Ultra II Q5 Master Mix (New England BioLabs, M0544L); Cas9-NLS (QB3 MacroLab at University of California, Berkeley); CloneR (StemCell Technologies, 05889); MiSeq reagent nano kit v2 (300 cycles; Illumina, MS-103-1001); GeneArt Precision gRNA synthesis kit (Thermo Fisher Scientific, A29377); RNAeasy Qiagen kit (Qiagen, 74104); 24-well glass-bottom plates (Cellvis, P24-1.5H-N); Corning square culture dish (Corning, 431110); Nunc Nunclon Delta cell culture dishes (Thermo Scientific, 140675, 150318 and 168381); Corning Matrigel matrix (Corning, 354230); DMEM with F-12 (Gibco, 11330057); neurobasal medium (Thermo Scientific, 21103049); non-essential amino acids (Gibco, 11140050); GlutaMAX (Gibco, 35050061); N-2 supplement (Gibco, 17502048); neurotrophin-3 (NT3; Peprotech, 450-03); brain-derived neurotrophic factor (BDNF; Peprotech, 450-02); B27 (Gibco, 17504001); Y27632 dihydrochloride (ROCK inhibitor; PeproTech, 1293823); Cultrex 3D culture matrix laminin I (R&D Systems, 3446-005-01); accutase (StemCell Technologies, 07922); FGF2-G3 (in-house); human insulin (Santa Cruz Biotechnologies, sc-360248); transforming growth factor-β (PeproTech, 100-21); holo-transferrin human (Sigma-Aldrich, T0665); sodium bicarbonate (Sigma-Aldrich, S5761-500G); sodium selenite (Sigma-Aldrich, S5261-10G); doxycycline (Clontech Labs, 631311); UltraPure 0.5 M EDTA (Invitrogen, 15575020); 16% paraformaldehyde (Electron Microscopy Science, 15710); DMEM (Gibco, 11995073); fetal bovine serum (Cytiva, SH30910.03); hydrocortisone (Sigma-Aldrich, H0135); polyethylenimine (Polysciences, 23966); FuGENE (Promega, E2311).

The following primary antibodies were used (1:1,000 for immunoblotting, 1:400 for immunofluorescence): Flag (Sigma-Aldrich, F1804), HA (Cell Signaling Technology, 3724), V5 (Invitrogen, 14-6796-82), TMEM230 (Origene, TA504888), LAMP1 (Cell Signaling Technology, D2D11), RAB5 (Cell Signaling Technology, C8B1), CLR (ProteinTech, 10292-1-AP), golgin 97 (ProteinTech, 12640-1-AP), VDAC1 (ProteinTech, 55259-1-AP), CLCN3 (Cell Signaling Technology, 13359S), GFP (Thermo Scientific, a10262), mCh (Thermo Scientific, M11217), EEA1 (Cell Signaling Technology, C45B10). The following secondary antibodies were used (1:10,000 for immunoblotting, 1:400 for immunofluorescence): anti-rabbit immunoglobulin-G (IgG) horse radish peroxidase (HRP) conjugate (Bio-Rad, 1706515); anti-mouse IgG HRP conjugate (Bio-Rad, 1706516); goat anti-chicken IgY (H + L), Alexa Fluor 488 (Thermo Scientific, A-11039); goat anti-rat IgG (H + L) cross-adsorbed, Alexa Fluor 555 (Thermo Scientific, A-21434); goat anti-rabbit IgG (H + L) cross-adsorbed, Alexa Fluor 647 (Thermo Scientific, A-21244).

### Molecular cloning

Plasmids were made as previously described^61^. Entry clones from the human ORFeome collection, version 8, were cloned into their corresponding plasmids using Gateway technology (Thermo Fisher Scientific) or Gibson assembly (New England Biolabs). The complete TMEM230(Y29C/R78L/X121W) mutant was obtained by gene synthesis (Twist Bioscience). For lentivirus transduction, pHAGE and pLenti backbones were used. For transfection, pCGS and pcDNA3.1 backbones were used. The following plasmids were generated: pGCS-3×Flag-ATP11B (Addgene, 225511), pcDNA-TMEM30A-V5 (Addgene, 225510), pGCS-3×HA-TMEM230 (Addgene, 225512), pGCS-3×HA-TMEM230(Y29C/R78L/X121W) (Addgene 225513), pLenti-UBC-HA-TMEM230 (Addgene, 225516), pLenti-UBC-HA-TMEM230(R78L) (Addgene, 225517), pLenti-UBC-HA-TMEM230(X121W) (Addgene, 225519), pLenti-UBC-HA-TMEM230(Y29C) (Addgene, 225520), pLenti-UBC-HA-TMEM230(X121PG) (Addgene, 225518), pLenti-UBC-HA-TMEM230(Y29C/R78L/X121W) (Addgene 225521), pcDNA-CLCN3-3×Flag (Addgene, 225506), pcDNA-CLCN5-3×Flag (Addgene, 225507), pcDNA-TMEM9B-3×HA (Addgene, 225509), pcDNA-TMEM9-3×HA (Addgene, 225508), pHAGE-mCh-CLCN3 (Addgene, 225514), pHAGE-TMEM9-EGFP (Addgene, 225515). The following plasmids were used for lentiviral packaging: pPAX2 (Addgene, 12259), pMD2 (Addgene, 12260).

### Cell culture, neuronal differentiation and lentiviral transduction

HEK293 cells (ATCC; RRID:CVCL_0045) were cultured in 10-cm dishes with high-glucose and pyruvate DMEM supplemented with 10% fetal bovine serum. For co-IP experiments, cells were transfected at 60% confluency with 6 μg of plasmids in a 2:1 ratio using polyethylenimine (25 kDa) and incubated for 48 h at 37 °C and 5% CO_2_. SUM159PT cells (a gift from T. Walter, Memorial Sloan Kettering; RRID:CVCL_5423) were cultured in 6-well culture dishes (300,000 cells per well) in DMEM with F-12 supplemented with GlutaMAX, 5% fetal bovine serum, 1 μg ml^−1^ hydrocortisone and 5 μg ml^−1^ insulin. Cells were transfected 1 day later with 500 ng of plasmids using FuGENE and Optimem transfection reagent and incubated at 37 °C and 5% CO_2_. One day after transfection, cells were selected with puromycin and plated into 24-well glass-bottom culture dishes (50,000–100,000 cells per well).

Gene-edited human embryonic stem (ES) cells (H9, WiCell Institute) were cultured as described previously^62^,^63^. Cells were maintained with E8 medium on plates coated with Matrigel and split with 0.5 mM EDTA in DPBS. ATCC performs quality testing to ensure authentication of the HEK293T cell line using short tandem repeat analysis. H9 ES cells (from WiCell) are authenticated by WiCell using G-band karyotyping and short tandem repeat analysis. Genetically edited H9 human ES cells were confirmed by karyotyping. HEK293, SUM159T and H9 cell lines were tested for mycoplasma on a monthly basis using Mycoplasma Plus PCR assay kit (Agilent 302107). Use of H9 cells for this study was approved by the Embryonic Stem Cell Research Oversight Committee (approval number 00051).

Human ES cells with the AAVS1-TRE3G-NGN2 driver^64^ were differentiated into iNeurons as described previously^65^. Briefly, stem cells were plated at 2 × 10^5^ cells ml^−1^ (differentiation day 0) in ND1 medium (DMEM with F-12, N-2, human 10 ng ml^−1^ BDNF, 10 ng ml^−1^ human NT3, non-essential amino acids, 0.2 μg ml^−1^ human laminin) supplemented with 2 mg ml^−1^ doxycycline and 10 μM Y27632 (ROCK inhibitor). The next day, the medium was exchanged with ND1 without Y27632. The following day, the medium was replaced with ND2 (neurobasal medium, B27, GlutaMAX, 10 ng ml^−1^ BDNF, 10 ng ml^−1^ NT3) supplemented with 2 μg ml^−1^ doxycycline. Until the experimental day (day 19–21), 50% of the medium was replaced with fresh ND2 every other day. Cells were replated at 4 × 10^5^ cells per well on day 4–6. From day 10, doxycycline was removed from the ND2.

Lentiviral vectors were packed in HEK293T cells (ATCC number CRL-3216; RRID:CVCL_0045) as described previously^62,66,67^. Cells were co-transfected at 60% confluency with pPAX2, pMD2 and the target vector in a 4:2:1 ratio using polyethylenimine. The medium was changed to ND2 the next day and collected 2 days after transfection. ND2 medium containing lentivirus was filtered (0.22 μm) and used for transduction of iNeurons at differentiation day 11–12.

### CRISPR–Cas9 gene editing

Human ES cells (H9 AAVS1-TRE3G-NGN2 3×Flag–EEA1; RRID:CVCL_D1KV) were gene-edited using CRISPR–Cas9 (ref. ^68^). Cells were electroporated with a mixture of 0.6 μg guide RNA and 3 μg Cas9-NLS (QB3 MacroLab, University of California, Berkeley) using a Neon transfection system as previously described^69^ according to the specific protocol at ref. ^70^. To generate human ES cells homozygous for the *TMEM230*^X121W^ variant, a single-stranded DNA oligonucleotide was included in the electroporation (5′-CTACCGTGGTTACTCCTATGATGACATTCCAGACTTTGATGACTGGCACCCACCCCATAGCTGAGGAGGAGTCACAGTGGAACTGTCCCAGCTTTAAGATATCTAGCAGAAACTATAGCTG-3′). The cells were recovered for 24–48 h in a low-O_2_ incubator and sorted into single cells with a Sony Biotechnology (SH800S) cell sorter (RRID:SCR_018066). Gene editing of individual clones was verified by sequencing with the Illumina MiSeq system (RRID:SCR_016379) and validated by immunoblotting and/or MS. Guide RNAs were generated using the GeneArt Precision gRNA synthesis kit (Thermo Fisher Scientific) for the sequences: *TMEM230*^*−/−*^ 5′-CCTGAAGGTCAATGTAGCCATCGT-3′, *TMEM230*^X121W^ 5′-CTCCTCCTCAGCTATGGGGT-3′, *TMEM9*^*−/−*^ 5′-TATCTTTGGTGGCTGTGGTC-3′, *TMEM9B*^*−/−*^ 5′-TCTACATCAGGCCCCCGCAC-3′. ES cells reported here will be made available upon request, but require a Material Transfer Agreement from WiCell.

### Spinning-disc confocal microscopy

For immunofluorescence staining, SUM159PT cells were fixed with 4% paraformaldehyde in PBS for 15 min and permeabilized with 0.5% Triton X-100 in PBS for 10 min at room temperature. Cells were blocked with 3% BSA in PBS with 0.1% Triton X-100 for 1 h at room temperature. Cells were incubated with primary antibodies (1:200 dilution) in 3% BSA in PBS with 0.1% Triton X-100 for 3 h at 4 °C. After washes, cells were incubated with Alexa Fluor secondary antibodies (1:400) for 1 h at 4 °C, and nuclei were stained with Hoechst 33342 (1:10,000) for 5 min. Cells were washed and maintained in PBS until microscopy analysis. Immunostaining of iNeurons was performed according to the protocol at ref. ^71^.

Cells were imaged using a Yokogawa CSU-X1 spinning-disc confocal on a Nikon Eclipse Ti-E motorized microscope and a Plan Apochromat 100× 1.45 N.A oil-objective lens. Live-cell imaging was performed with a Tokai Hit stage top incubator at 37 °C, 5% CO_2_ and 95% humidity. Images were acquired with a Hamamatsu ORCA-Fusion BT CMOS camera (6.5 μm^2^ photodiode, 16-bit) and NIS-Elements image acquisition software (RRID:SCR_002776). All samples were measured under the same exposure time and laser power. Co-localization analysis was performed with the JACoP plugin (RRID:SCR_025164) for ImageJ/FiJi (RRID:SCR_002285)^72^ using maximum-intensity projection images and maximum entropy threshold. Linear mixed-effect model statistics were applied as implemented in the lme4 R package with a nested design to account for images acquired from the same culture well and same biological replicate. The number of fields of view for each of the three independent biological replicates is indicated in the figures (Fig. 4e,f).

### Endosomal scoring method

The scoring method was performed to define the endosomal proteome and assign an unbiased score to each protein reflecting the probability of being located in endosomes based on experimental data. The literature was surveyed for studies capturing the endosomal proteome in mammalian organisms, which resulted in 16 datasets^18,19,73–82^ (Supplementary Table 1). Incomplete datasets or with ambiguous organelle purifications (for example, ‘vesicles’ or mixed organelles) were excluded. Outdated Uniprot IDs and obsolete gene names were updated (Uniprot 2022-02). Ensembl and the BiomaRt R package (RRID:SCR_019214) were used to retrieve and match rodent genes to their human orthologues, including all human genes when multiple genes matched. Subsequent analyses were based on the protein identification across datasets as a metric for the scoring method (Supplementary Table 1). To evaluate the performance of scoring metrics and datasets, a reference list of 292 well-known endosomal proteins was manually curated from published literature^1,3,22,56,83–116^ (Extended Data Fig. 1a). Dataset overview was visualized by multiple correspondence analysis using the FactoMineR R package (RRID:SCR_014602; Extended Data Fig. 1b). Protein annotation to various organellar locations was based on a previous study^62^ (Extended Data Fig. 1c). Another metric of the endosomal scoring was the protein abundance in Endo-IP obtained from the label-free proteomic analysis of endosomal pellets as described below (Extended Data Fig. 1d and Supplementary Table 1). The number of interactions with endosomal proteins was obtained from BioPlex 3.0 (RRID:SCR_016144), STRINGDB and CORUM (28.11.2022 Corum 4.1 release)^28,117,118^ for the reference list of well-known endosomal proteins described above. For STRINGDB, only physical interactions with experimental evidence or databases with high score (combined score >0.7) were included.

The performance of each metric to classify endosomal proteins (from the reference list described above) was evaluated by receiver operating characteristic curves using the pROC R package with a binomial logistic regression as the predictor (Fig. 1b). The combined endosomal score was obtained by summation of the three individual metrics. Partial area under the curve and the threshold to consider a protein as endosomal was 10% false positives based on the reference list. The scoring method resulted in 407 predicted endosomal proteins (14 proteins present in MitoCarta3.0 (RRID:SCR_018165) were excluded) that were combined with the reference list of well-known endosomal protein for a total of 522 proteins (Supplementary Table 1). These proteins were characterized using BioPlex 3.0 (ref. ^28^), OpenCell (RRID:SCR_021870)^119^, and publications as retrieved from Uniprot (Extended Data Fig. 1e–g). Endosomal annotation for all subsequent analyses was based on this list.

### EEA1^+^ endosome purification through Endo-IP affinity capture

Endo-IPs with HEK293^EL^ cells were performed as described previously^120^. HEK293^EL^ cells expressing Flag–EEA1 (ref. ^19^) were collected from five 24.5-cm square culture dishes per replicate for co-fractionation experiments (*n* = 3) and 60 square plates per replicate (divided into two batches) for crosslinking experiments (*n* = 2). Endo-IPs in iNeurons were performed as described previously^44,121^. Three 15-cm culture dishes per replicate were used for experiments in iNeurons (*n* = 3). Cells were pelleted at 1,000*g* for 2 min at 4 °C and washed once with KPBS buffer (100 mM potassium phosphate, 25 mM KCl and protease inhibitor cocktail, pH 7.2). Cell pellets were resuspended in KPBS and lysed in a Dounce homogenizer with 25 strokes. Samples were centrifuged twice at 1,000*g* for 5 min at 4 °C, and PNS protein concentration was quantified and normalized by Bradford assay. Samples were incubated for 50 min at 4 °C with 70 μl anti-Flag Sigma magnetic beads for iNeurons experiments, 1.6 ml Sigma anti-Flag Sigma magnetic beads for co-fractionation experiments and 20 ml of anti-Flag Pierce magnetic beads per batch for crosslinking experiments. The beads were washed four times using a magnetic stand with KPBS. For quantitative proteomics, endosomes were eluted with 120 µl 0.5% NP40 (IGEPAL) in KBPS for 30 min at 4 °C and stored at −80 °C until MS sample preparation. For co-fractionation and crosslinking experiments, endosomes were eluted twice with 0.8 mM 3×Flag peptide in KPBS for 45 min at 4 °C (Extended Data Fig. 1h). Peptide-eluted samples were centrifuged for 20 min at 10,000*g* in Posi-Click tubes (Denville, c2170). Endosomal pellets were washed twice with KPBS to remove excess 3×Flag peptide and immediately processed. An additional wash was performed for the second replicate of the crosslinking experiment, which helped increase the coverage in the MS analysis.

### Protein co-IP

A protocol for this analysis is available at ref. ^122^. Proteins from a 10-cm culture dish of HEK293 cells or a 15-cm dish of iNeurons per replicate (*n* = 2 or 4) were extracted for 1 h at 4 °C with 0.5% DDM in 25 mM HEPES pH 7.4, 150 mM NaCl and protease inhibitor cocktail^123^. Samples were centrifuged twice at 20,000*g* for 20 min, and the supernatant was incubated with 15 μl anti-HA magnetic beads (Pierce) or 25 µl anti-Flag magnetic beads (Sigma) depending on the protein tag for 2 h at 4 °C. For IP using endogenous antibodies, the supernatant was incubated overnight with 5 μg of antibody before the incubation with 15 μl magnetic A/G beads. The beads were separated with a magnetic stand and washed four times with washing buffer (0.1% DDM, 25 mM HEPES, 150 mM NaCl, pH 7.4). Proteins bound to the beads were eluted with 30 μl 1.5× Laemmli buffer for immunoblotting or 30 μl 1.5× S-Trap lysis buffer (7.5% SDS, 150 mM TEAB pH 8.5) for MS analysis and heated at 80 °C for 5 min.

### SDS–PAGE immunoblotting

Samples mixed with Laemmli buffer were incubated at 80 °C for 5 min and loaded in a Criterion TGX stain-free precast gel for subsequent immunoblotting. After electrophoresis, gels were scanned using a Bio-Rad ChemiDoc imager (Bio-Rad) and electro-transferred onto a PVDF membrane overnight at 10 V. Membranes were blocked with 5% non-fat milk, and incubated with primary antibody for 2 h at 4 °C and subsequently with HRP-conjugated secondary antibodies for 1 h at 4 °C. After washing, blot images were acquired in a Bio-Rad ChemiDoc imager using SuperSignal West Pico PLUS Chemiluminescence substrate (Thermo Fisher, catalogue number 34580). Images were processed with Bio-Rad Image Lab software (version 6.1.0; RRID:SCR_014210). Differences in loading were normalized using the stain-free quantification of total protein amount. Protocols for this procedure are available at ref. ^124^. Full versions of all gels and blots are available in Supplementary Fig. 1.

### BN electrophoresis co-fractionation and in-gel digestion

A detailed protocol for this procedure is available at ref. ^125^. Protein complexes from three independent biological Endo-IP replicates were fractionated and processed as previously described^126^. Freshly prepared purified endosomal pellets were resuspended in 40 μl KPBS with 0.5% DDM, and proteins were extracted for 45 min at 4 °C in rotation. Protein extracts were clarified by centrifugation at 20,000*g* and mixed with 10 μl BN loading buffer, 1 μl Coomassie G-250 mix and 0.5 μl native molecular weight marker. Samples were run on a 4–16% NativePAGE gel at 150 V for 1.5 h and at 250 V for 20 min at 4 °C. Gels were fixed in 50% ethanol and 3% phosphoric acid, followed by staining with Coomassie. Each sample was cut into 48 1-mm slices and transferred to a 96-well filter plate for in-gel digestion^123^. Briefly, proteins were reduced with 100 µl 5 mM TCEP in 50 mM ammonium bicarbonate for 30 min at 37 °C. Proteins were alkylated with 20 mM chloroacetamide in 50 mM ammonium bicarbonate for 15 min at room temperature. Fractions were destained, dried and digested with 0.2 μg Lys-C for 4 h at 37 °C followed by overnight incubation with 0.2 μg of trypsin. Peptides were extracted, dried in a SpeedVac and reconstituted in 5% acetronitrile (ACN), 5% formic acid for data-independent acquisition (DIA) liquid chromatography (LC)–MS/MS analysis.

### Crosslinking and strong cation exchange fractionation

A detailed protocol for both crosslinking procedures is available at ref. ^127^. Freshly prepared purified endosomal pellets from two independent biological replicates were resuspended in 300 μl KPBS and immediately crosslinked by incubating with 1 mM DSSO (disuccinimidyl sulfoxide, with the full chemical name bis(2,5-dioxopyrrolidin-1-yl) 3,3′-sulfinyldipropionate, bis-(propionic acid NHS ester)-sulfoxide, Thermo Fisher Scientific) at room temperature for 40 min (ref. ^6^). The reaction was quenched with 50 mM Tris buffer pH 7.5 at room temperature for 30 min. Crosslinked samples were denatured in 8 M urea, reduced with 5 mM dithiothreitol for 30 min at 37 °C, and alkylated with 40 mM chloroacetamide for 30 min at room temperature. Crosslinked proteins were digested with Lys-C (1:75) at 37 °C overnight. Sample urea concentration was diluted to 2 M with 50 mM 3-[4-(2-hydroxyethyl)-1-piperazine]propanesulfonic acid and incubated at 37 °C with trypsin (1:100) for 6 h. Peptides were desalted with a 50 mg C8 Sep-Pak solid-phase extraction column, dried and fractionated by strong cation exchange chromatography. A 70-min linear gradient of mobile phase (0.5 M NaCl in 20% ACN, 0.05% formic acid) was used from 0 to 8% in 14 min, to 20% at 28 min, to 40% at 48 min and to 90% at 68 min at a column flow rate of 0.18 ml min^−1^ in a PolyLC PolySulfoethyl A column (3 μm particle size, 2.1 mm inner diameter and 100 mm length). Fractions were collected every 30 s starting at 35 min for 10 min, and then every minute. Fractions were dried in a SpeedVac and desalted using a C8 StageTip. Around 30 fractions for each sample were reconstituted in 5% ACN, 5% formic acid and analysed by LC–MS/MS.

An additional independent biological replicate of freshly prepared purified endosomal pellet was resuspended in 300 μl KPBS and immediately crosslinked by incubating with a combination of 8 mM DHSO and 16 mM DMTMM at 37 °C for 90 min (ref. ^32^). Crosslinked samples were denatured in 5% SDS and briefly sonicated, reduced with 5 mM dithiothreitol for 5 min at 55 °C, and alkylated with 20 mM MMTS. Crosslinked proteins were precipitated and subjected to the S-Trap mini-spin column digestion protocol as provided by the manufacturer (see below). Peptides were desalted and fractionated by strong cation exchange chromatography as described above. A total of 30 fractions were analysed by LC–MS/MS.

### S-Trap sample preparation

Three independent replicates of PNS samples (10 μg or 50 μg of protein depending on the experiment) and Endo-IP samples were mixed with equal volume of water and subjected to sample preparation. The S-Trap micro-spin column digestion protocol (version 4.7) was followed as provided by the manufacturer (Protifi, C02-micro-80)^128–130^. Briefly, each sample was mixed with equal volumes of 2× lysis buffer (10% SDS, 100 mM TEAB buffer pH 8.5). Protein IP samples from iNeurons (*n* = 2 or 4) were directly collected in 1.5× lysis buffer. Proteins were reduced by incubating at 55 °C for 30 min with 5 mM TCEP and alkylated for 30 min at room temperature with 40 mM chloroacetamide. Samples were acidified with phosphoric acid and mixed with washing buffer (90% methanol, 100 mM TEAB buffer pH 7.55). Samples were transferred to micro-spin columns and washed 4 times with 150 µl washing buffer by centrifugation. Proteins were digested with 0.5 µg Lys-C at 37 °C overnight in a humid chamber, followed by 6 h incubation with 0.5 μg of trypsin. Peptides were collected from the column by three subsequent centrifugation steps (with 50 mM TEAB buffer, 0.2% formic acid and 50% ACN, respectively) and dried in a SpeedVac.

### TMT labelling and peptide fractionation

Protocols for labelling of peptides are available at ref. ^131^. Peptides were resuspended in 50 μl (PNS samples) or 35 μl (Endo-IP samples) 100 mM TEAB buffer pH 8.5. PNS and Endo-IP peptides were labelled by adding 11 μl or 7 μl ACN, and incubating for 1 h at room temperature with 10 μl or 8 μl of TMTpro reagent (20 mg ml^−1^ stock in ACN), respectively. The reaction was quenched by adding 10 μl 5% hydroxylamine for 15 min.

For PNS samples, equal peptide amounts for each sample were combined, desalted with a 100 mg C18 Sep-Pak solid-phase extraction column and fractionated by basic pH reversed-phase high-performance LC. Chromatography was performed with a 50-min linear gradient from 5% to 35% ACN in 10 mM ammonium bicarbonate pH 8 at a column flow rate of 0.25 ml min^−1^ using an Agilent 300 Extend C18 column (3.5 μm particle size, 2.1 mm inner diameter and 250 mm length). The initial 96 fractions collected were combined into 24 fractions, as described previously^132^. One set of 12 non-adjacent fractions were dried in a SpeedVac and desalted using C18 StageTip. Dried peptides were reconstituted in 5% ACN, 5% formic acid and subjected to LC–MS/MS analysis.

For Endo-IP samples, equal peptide amounts for each sample were combined and fractionated using a high-pH reversed-phase peptide fractionation kit (Pierce) following the manufacturer’s protocol. Eluates were combined into six fractions, dried and desalted using C18 StageTip. Dried peptides were reconstituted in 5% ACN, 5% formic acid and subjected to LC–MS/MS analysis.

For protein IP samples, equal peptide amounts for each sample were combined, dried and desalted using C18 StageTip without further fractionation. Dried peptides were reconstituted in 5% ACN, 5% formic acid and subjected to LC–MS/MS analysis.

### LC–MS data acquisition

TMT-labelled samples were analysed using a Vanquish Neo UHPLC system coupled to an Orbitrap Eclipse Tribid mass spectrometer (RRID:SCR_020559) with FAIMS Pro (ref. ^131^). Peptides were separated on a 100-μm microcapillary column packed with 20 cm of Accucore C18 resin (2.6 μm, 150 Å). A 90-min linear gradient from 5% to 20% ACN in 80 min, to 36% at 83 min, and to 98% at 85 min in 0.125% formic acid was used at 0.3 µl min^−1^. MS^1^ spectra were acquired on the Orbitrap (resolution 60,000, scan range 350–1,350 *m*/*z*, standard automatic gain control (AGC) target, auto maximum injection time). Peptide fragmentation was achieved by high-energy collisional dissociation (HCD) at 36% normalized collision energy. MS^2^ spectra were acquired on the Orbitrap (resolution 30,000, isolation window 0.6 *m*/*z*, TurboTMT set to All TMT Reagents, first mass 120 *m*/*z*, 200% normalized AGC, 120 ms maximum injection time). FAIMS Pro was set to −30, −50 and −70 compensation voltage (CV). Unfractionated samples (protein IPs) were injected twice with FAIMS set to −40, −60 and −80 CV for the second run.

BN-PAGE co-fractionation samples were analysed using an EASY-nLC 1200 system coupled to an Orbitrap Exploris 480 mass spectrometer (RRID:SCR_022215). A 15-cm 100-μm capillary column was packed in-house with Accucore 150 C18 resin (2.6 μm, 150 Å). A 90-min linear gradient from 5% to 20% ACN in 80 min, to 25% at 83 min, and to 98% at 85 min in 0.125% formic acid was used at 0.3 µl min^−1^. The DIA method consisted of MS^2^ analysis of overlapping isolation windows of 24 *m*/*z* stepped through 390–1,014 *m*/*z* mass range for the first cycle and 402–1,026 *m*/*z* for the second cycle^133^. DIA scans were performed with 28% normalized HCD collision energy, 30,000 resolution, 145–1,450 *m*/*z* scan range, 1,000% normalized AGC and 54 ms maximum injection time. This was followed by a parent MS^1^ ion scan (resolution 60,000, scan range 350–1,050 *m*/*z*, 100% normalized AGC target, auto maximum injection time).

DSSO-crosslinking samples were analysed using an EASY-nLC 1200 system coupled to an Orbitrap Fusion Lumos mass spectrometer with FAIMS Pro (RRID:SCR_020562). A 90-min linear gradient from 5% to 20% ACN in 80 min, to 25% at 83 min, to 40% at 85 min, and to 98% for 2 min in 0.125% formic acid was used at 0.5 µl min^−1^. An HCD-MS2 strategy was used^4^, in which the MS^1^ spectrum was acquired on the Orbitrap (resolution 120,000, scan range 400–1,600 *m*/*z*, standard AGC target, auto maximum injection time). Peptides with charge states 4–8 were fragmented by HCD at 21, 27 and 33% normalized collision energy. MS^2^ was acquired on the Orbitrap (resolution 60,000, isolation window 1.6 *m*/*z*, auto scan range, 200% normalized AGC, 120 ms maximum injection time). FAIMS Pro was set to −50, −60 and −75 CV (ref. ^134^).

DHSO- and DMTMM-crosslinking samples were analysed using a Vanquish Neo UHPLC system coupled to an Orbitrap Ascend MultiOmics Tribid mass spectrometer with FAIMS Pro. A 90-min linear gradient from 5% to 20% ACN in 80 min, to 25% at 83 min, to 40% at 85 min, and to 98% for 2 min in 0.125% formic acid was used at 0.3 µl min^−1^. MS^1^ spectrum was acquired on the Orbitrap (resolution 120,000, scan range 350–1,600 *m*/*z*, standard AGC target, auto maximum injection time). Peptides with charge states 4–8 were fragmented by HCD at 21, 27 and 33% normalized collision energy. MS^2^ was acquired on the Orbitrap (resolution 60,000, isolation window 1.4 *m*/*z*, auto scan range, 200% normalized AGC, 120 ms maximum injection time). FAIMS Pro was set to −50, −60 and −75 CV.

### Proteomics data analysis

TMT-MS data were processed with MSconverter^135^ and searched using Comet^136^ against the human canonical proteome (UniProt Swiss-Prot 2021-11), including reverse sequences and common contaminants. Experiments containing variants of TMEM230 were searched against the human canonical proteome (UniProt Swiss-Prot 2024-01) including an additional sequence of TMEM230 with such variants. Peptide mass tolerance was set to 50 ppm and fragment ion tolerance to 0.02 Da. These wide mass tolerance windows were chosen to maximize sensitivity in conjunction with Comet searches and linear discriminant analysis^137^. TMTpro labels were set as fixed modification on lysines and peptide N terminus (+304.207 Da), carboxyamidomethylation on cysteines (+57.021 Da) as a fixed modification, and oxidation on methionine residues as a variable modification. Linear discriminant analysis was performed^138^ and peptide-spectrum matches (PSMs) were filtered to 2% FDR^139^. TMT-reporter ions were quantified by picking the most intense peaks within 0.003 Da around the theoretical *m*/*z*, and corrected for isotopic impurity. Only PSMs with at least 200 total signal-to-noise ratio across all TMT channels and 50% precursor isolation purity were used^140^. Data summarization, normalization and statistics were performed using MSstats^141,142^. Peptide-level normalization and imputation were enabled, and the protein summarization method was set to ‘LogSum’ for Endo-IP experiments from iNeurons and to ‘msstats’ for all other experiments. The threshold used to consider significantly regulated proteins was 0.05 *q*-value and 1.5-fold change. For PNS and Endo-IP experiments with iNeurons, three biological replicates per condition were analysed (Supplementary Tables 4 and 5). For protein IP experiments in iNeurons, four biological replicates were analysed per group (Supplementary Table 5), except for one dataset with some groups containing two replicates given the limitation of the maximum number of TMT channels (Supplementary Table 4). Synaptic Gene Ontology enrichment analysis was performed using SynGO^143^ (https://www.syngoportal.org/#) using all proteins identified in each experiment as background.

DIA-MS data were analysed using DIA-NN (version 1.8) as previously described^144,145^. Data were converted to mzML using MSconvert^135^ with the Demultiplex filter set to Overlap Only (10-ppm mass error). A spectral library was generated from the complete human proteome (UniProt 2022-05) with a precursor *m*/*z* range of 350–1,050, precursor charge 2–5 and fragment ion *m*/*z* range 145–1,450. Carbamidomethylation, oxidation and N-terminal excision were included as modifications. Search was performed with 10-ppm mass accuracy, match-between-runs enabled and robust LC (high precision) quantification strategy. For Endo-IP protocol optimization samples (Extended Data Fig. 1i–l and Supplementary Table 1), downstream analysis was performed using MS-DAP^146^. Only peptides quantified in all three replicates per condition (*n* = 3) were included. Data were normalized with variance stabilization normalization and mode-between protein methods. The DEqMS algorithm was selected for statistical analysis, using a significance threshold of 0.01 FDR-adjusted *P*-value threshold and log_2_[fold change] of 3 (Supplementary Table 1). For BN co-fractionation experiments, protein complex analysis was performed with PCprophet^25^. Three biological replicates were analysed with default parameters, the provided core complexes were used as database and the BN markers were used for collapsing hypothesis to common complexes. As previously described^7^, co-elution scores (from rf output table) were assigned to each protein pair of the complex and used for downstream analysis. Only complexes with a minimum peak elution at 67-kDa and a maximum of 25 proteins per complex were considered. In addition, we considered only interactions with a score of at least 0.7 in two replicates to recover only high-confidence candidate interactions (Supplementary Table 2). These parameters were selected on the basis of the optimal recovery of protein interactions reported in BioPlex^7^ (Extended Data Fig. 2b,c). Elution profiles and Pearson’s correlation heat map of selected protein complexes based on CORUM^118^ were generated using the mean normalized elution profile across replicates (excluding outliers as the most dissimilar fraction to the median).

DSSO crosslinking MS data were analysed using Thermo Proteome Discoverer (version 2.5.0.400; RRID:SCR_014477) with the XlinkX module^147,148^. Data were searched against the human canonical proteome (Uniprot Swiss-Prot 2022-05). MS^2^ acquisition strategy was selected with 10-ppm precursor mass tolerance, 20 ppm FTMS fragment mass tolerance and 0.6 Da ITMS fragment mass tolerance. Carbamidomethylation was included as a fixed modification; oxidation and N-terminal acetylation were included as variable modifications. A maximum of three trypsin miscleavages was allowed, and the minimum peptide length was set to 5. FDR threshold was set to 5% and only crosslinks with XlinkX score >40 were considered for downstream analysis (Supplementary Table 2). Protein domain information of all crosslinked positions was retrieved from UniProt (Fig. 1f) and copy numbers were obtained from ref. ^18^ (Extended Data Fig. 2f). Yeast two-hybrid data were retrieved from ref. ^29^ and IP data from BioPlex 3.0 (ref. ^28^; Extended Data Fig. 2j). The co-fractionation of crosslinked protein pairs in the BN dataset was evaluated using SECAT^149^. Positive and negative interaction networks from CORUM were used as provided. The target network was generated from all of the crosslinking interactions for proteins identified in both crosslink and BN. The following parameters were used to ensure the generation of scores for all target protein pairs: peak picking was set to none, monomer threshold factor to 1, minimum abundance ratio to 0, maximum shift to 48 and maximum *q*-value of 1. SECAT *P* values were used for comparison with crosslink data and previously reported interactions from STRINGDB, CORUM and BioPlex 3.0 as described above (Extended Data Fig. 2l–n).

DHSO and DSSO crosslinking MS data were analysed using Scout (version 1.6.2)^31^. Data were searched against the human canonical proteome (Uniprot Swiss-Prot 2022-05) with default parameters, including 10-ppm error on the MS^1^ level and 20-ppm error on the MS^2^ level. Carbamidomethyl (mass 57.02146) and MMTS (mass 45.987721) were included as fixed modifications for DSSO- and DHSO-crosslinked samples, respectively; oxidation and N-terminal acetylation were included as variable modifications. A maximum of three trypsin miscleavages and two variable modifications was allowed and the minimum peptide length was set to 6. The FDR threshold was set to 1% at all levels without separation of crosslink types. The ‘residue pairs’ table was used for downstream analysis (Supplementary Table 2).

DMTMM crosslinking MS data were analysed using pLink2 (version 2.3.11, RRID:SCR_000084)^150^. Data were searched against the human canonical proteome (Uniprot Swiss-Prot 2022-05) with 15-ppm precursor mass tolerance and 20-ppm fragment mass tolerance. Methylthio(C) was included as a fixed modification; oxidation and N-terminal acetylation were included as variable modifications. A maximum of three trypsin miscleavages was allowed and the minimum peptide length was set to 6. Filter tolerance was set to 10 ppm and separated FDR threshold to 1% at the PSM level. Filtered crosslinked sites were used for downstream analysis (Supplementary Table 2). DMTMM and DHSO crosslinks were mapped to all possible protein interactions defined by DSSO crosslinks considering that each DMTMM or DHSO crosslink could match multiple interactions owing to shared peptide sequences.

### EndoMAP.v1 network analysis

A PPI network was generated from all protein pairs identified by crosslink and BN. The network was initially filtered to remove proteins present in the native molecular weight markers (spiked-in proteins used as reference in BN experiments), EEA1 (overexpressed and used as a handle for the endosome affinity purification), UBC (in most cases corresponds to a protein modification rather than a member of a protein complex) and keratins (common contaminant). Network characterization and analysis was performed using the igraph R package (RRID:SCR_021238; Extended Data Fig. 3a–c). Proteins were assigned to subcellular location according to the following annotations: endosomal proteins from our scoring method described above (Supplementary Table 1), Golgi proteins (as curated in ref. ^140^), lysosomal proteins (bona fide proteins in Table S3 from ref. ^151^; bona fide and experimentally determined proteins in Table S2 and Table S12 from ref. ^152^), mitochondrial proteins (from MitoCarta3.0 (ref. ^153^)) and nuclear proteins (based on Uniprot, proteins exclusively designated with nuclear-related terms such as ‘Nucleus’ and ‘Chromosome’). These annotations and the circlize R package (RRID:SCR_002141) were used to generate the network chord diagram (Extended Data Fig. 3d).

The network centred around endosomal proteins (or EndoMAP.v1) was generated by filtering dubious interactions (that is, nuclear proteins) and including only endosomal proteins (as defined by our scoring method) and their direct interactors. Up to 8.5% of the endosomal interactions involved nuclear proteins (Extended Data Fig. 3d), which may be considered questionable (therefore, were filtered out) and may indicate false connectivity at the PPI level introduced by sample preparation. Second-order interactors of endosomal proteins were included only when connected to at least one direct interactor by crosslink and/or two direct interactors by BN (Extended Data Fig. 3e). The core component of the network (that is, biggest module) was visualized using Cytoscape v3.10.1 (RRID:SCR_003032), and protein communities were detected by unsupervised edge-betweenness analysis (Fig. 2a). Gene Ontology (GO) enrichment analysis was performed for each community using g:Profiler (RRID:SCR_022865) with the whole proteome as background (Supplementary Table 2, including only significant GO Cellular Component, GO Biological Process and CORUM terms with at least two proteins). Path distance analysis between proteins assigned to complexes was based on CORUM and GO:CC (only terms related to protein complexes; Fig. 3b and Extended Data Fig. 3f). Graph rewiring with the same degree distribution (100 permutations) was used as a randomized control (Fig. 3c). Disease over-representation analysis of the endosomal proteome was performed on endosomal proteins as defined by our scoring method and as annotated in GO (GO:0005768, date December 2024). Enrichment analysis for the gene network (DisGeNET)^154^ was performed as implemented in the DOSE R package. Enrichment analysis for neurodegenerative disorders included autism spectrum disorders, epilepsy and severe neurodevelopmental disorder, and schizophrenia was based on ref. ^155^, and was performed using the clusterProfiler R package (RRID:SCR_016884) with brain-expressed genes as background. Path distance analysis between proteins linked to neurodegenerative disorders was based on Diseases 2.0 (ref. ^156^; 2024-02 update; RRID:SCR_015664), Parkinson’s disease reviewed genes^16^ and Parkinson’s disease genome-wide association studies^157,158^ (Extended Data Fig. 3j,k).

### AF-M, AlphaLink2 and structural modelling

AF-M was run with ColabFold v1.5.2 (ref. ^8^; RRID:SCR_025453) on 40-GB A100 NVIDIA GPUs for all protein pairs identified by XL–MS and three-clique combinations within EndoMAP.v1 (with a maximum of 3,600 amino acids in total). AF-M version 3 was used with weights models 1, 2 and 4 with three recycles, templates enabled, one ensemble, no dropout, and no AMBER relaxation. The multiple sequence alignments supplied to AF-M were generated by the MMSeq server (RRID:SCR_022962) with default settings (paired + unpaired sequences). SPOC and contact sites were calculated as described previously^30,159^. The quality of the predictions was considered acceptable with a SPOC > 0.33 for pairwise predictions and at least two interfaces with interface average models >0.5 for timer predictions. AlphaLink2 (https://github.com/lhatsk/AlphaLink) was performed as described previously^21^ using intraprotein and interprotein DSSO crosslinks. Three predictions for each protein pair were generated with AlphaLink2 by using different seeds.

All PDB structures containing protein pairs identified by XL–MS were retrieved by querying the PDB API for X-ray and cryogenic electron microscopy structures with overall resolutions <3.5 Å. PDB chains were mapped to their corresponding UniProt identifiers with PDB SIFTS API. Crosslinks were mapped onto the AF-M and PDB structures, and crosslinked residues with a maximum Cα–Cα distance of 35 Å were considered to match the crosslinker constraints. For AlphaLink2, the maximum Cα–Cα distance considered was 30 Å for all crosslinkers, a more stringent threshold as DSSO crosslinks were already used to assist the prediction generation. For AF-M and AlphaLink2 predictions, only crosslinked residues with both pLDDTs >70 were considered for distance analysis. Crosslinks involving HSP90AA1 and HSP90AB1, which present a large number of crosslinks, were excluded from the distance distribution plots in AlphaLink2 predictions (Extended Data Fig. 4m–o) to make the analysis more representative of the entire dataset.

The association of mTORC1–ragulator complex with V-ATPase was modelled using HADDOCK2.4 web server^160^ (RRID:SCR_019091). The crosslinks identified between ATP6V1C1–LAMPTOR2 and ATP6V1C1–LAMPTOR4 were used as unambiguous restraints with an upper distance limit of 23 Å and centre-of-mass restraints enabled. The complete mTORC1–ragulator complex structure (PDB 7UXH)^59^ was included with selected subunits of V-ATPase owing to the limitation in the maximum number of atoms (PDB 6WM2 chains I and J from ATP6V1E1, chains L and M from ATP6V1G1, chain O from ATP6V1C1, chains 8 and 9 from ATP6V0C)^58^. The hypothetical model with the best score compatible with the expected membrane topology was selected (cluster 5; Extended Data Fig. 11b). Structure images were generated with PyMOL 2.6.0 (RRID:SCR_000305). All input, parameter and output files are available via Zenodo at 10.5281/zenodo.14679635.

### Software and resources

The following software, packages and resources were additionally used for analysis and visualization: Rstudio (2023.06.0 Build 421 with R 4.2.1, RRID:SCR_001905); R package ggplot2 (3.5.1, RRID:SCR_014601); R package RColorBrewer (1.1.3, SCR_016697); R package ggrepel (0.9.5, RRID:SCR_016223); R package dplyr (1.1.4); R package FactoMineR (2.11, RRID:SCR_014602); R package pheatmap (1.0.12, RRID:SCR_016418); R package factoextra (1.0.7, RRID:SCR_016692); R package pROC (1.18.5); R package reshape2 (1.4.4); R package igraph (2.1.2); R package tidyr (1.3.1, RRID:SCR_017102); R package lme4 (1.1.13.5, RRID:SCR_015654); R package ggsignif (0.6.4, RRID:SCR_023047); R package viridis (0.6.5) (RRID:SCR_016696); Adobe Illustrator (26.5); NIAID NIH BioArt Source.

### Statistics and reproducibility

Sample size, number of replicates and statistical tests are indicated in the figure legends and corresponding sections of the Methods. Validation and representative experiments in Fig. 3d,h and Extended Data Figs. 5c and 7g were performed once, those in Extended Data Figs. 5e,h,k,m and 8c were performed twice, and those in Fig. 4g and Extended Data Fig. 7f were performed three times, with similar results in independent experiments.

### Reporting summary

Further information on research design is available in the Nature Portfolio Reporting Summary linked to this article.

## Online content

Any methods, additional references, Nature Portfolio reporting summaries, source data, extended data, supplementary information, acknowledgements, peer review information; details of author contributions and competing interests; and statements of data and code availability are available at 10.1038/s41586-025-09059-y.

## Supplementary information

Supplementary Fig. 1 Uncropped images for immunoblots and gels associated with all figures.

Reporting Summary

Supplementary Text Extension of our presentation of multiple facets of this paper.

Supplementary Tables Supplementary Tables 1–6.

Supplementary Video 1 Live-cell imaging showing subcellular localization of mCh–CLCN3 and TMEM9–GFP in SUM159 cells (*t* = 2 min).

## Source data

Source Data Fig. 1

Source Data Fig. 2

Source Data Fig. 3

Source Data Fig. 4

Source Data Extended Data Fig. 1

Source Data Extended Data Fig. 2

Source Data Extended Data Fig. 3

Source Data Extended Data Fig. 4

Source Data Extended Data Fig. 6

Source Data Extended Data Fig. 7

## Data Availability

All the MS proteomics data (289 .RAW files) have been deposited to the ProteomeXchange Consortium via the PRIDE repository (http://www.proteomexchange.org/; project accessions PXD054684, PXD054728, PXD059547 and PXD054765). The data, code, protocols and key laboratory materials used and generated in this study are listed in a Key Resource Table alongside their persistent identifiers at Zenodo (10.5281/zenodo.14180546 (ref. ^161^) and 10.5281/zenodo.14180545 (ref. ^162^)). All AF-M and AlphaLink2 predictions can be downloaded from https://endomap.hms.harvard.edu (RRID:SCR_026690) and have also been deposited at Zenodo (10.5281/zenodo.14447604 (ref. ^163^) and 10.5281/zenodo.14632928 (ref. ^164^)). Input and output files used for modelling the mTORC1–ragulator–V-ATPase complex using HADDOCK2.4 have been deposited at Zenodo (10.5281/zenodo.14679635 (ref. ^165^)). Raw imaging data have been deposited at Zenodo (10.5281/zenodo.14826176 (ref. ^166^) and 10.5281/zenodo.14828025 (ref. ^167^)). We used canonical protein entries from the human reference proteome database in our study (UniProt Swiss-Prot release 2021-11, 2022-05 and 2024-01; https://ftp.uniprot.org/pub/databases/uniprot/previous_major_releases/). Full versions of all gels and blots are available in Supplementary Fig. 1. Source data are provided with this paper.

## Code availability

Scripts for endosomal proteome scoring, co-fractionation, protein network and co-localization analysis have been deposited at GitHub (https://github.com/harperlaboratory/EndoMAP) and annotated at Zenodo (10.5281/zenodo.15109844 (ref. ^168^)).
