# Supplementary material for: EndoMAP.v1 charts the structural landscape of human early endosome complexes
Source: Nature. 2025 May 28;643(8070):252–61. doi: 10.1038/s41586-025-09059-y (PMC12222028; doi:10.1038/s41586-025-09059-y)

nature portfolio

https://doi.org/10.1038/s41586-025-09059-y

Supplementary information

EndoMAP.v1 charts the structural landscape of human early endosome complexes

In the format provided by the authors and unedited

Figure 3d

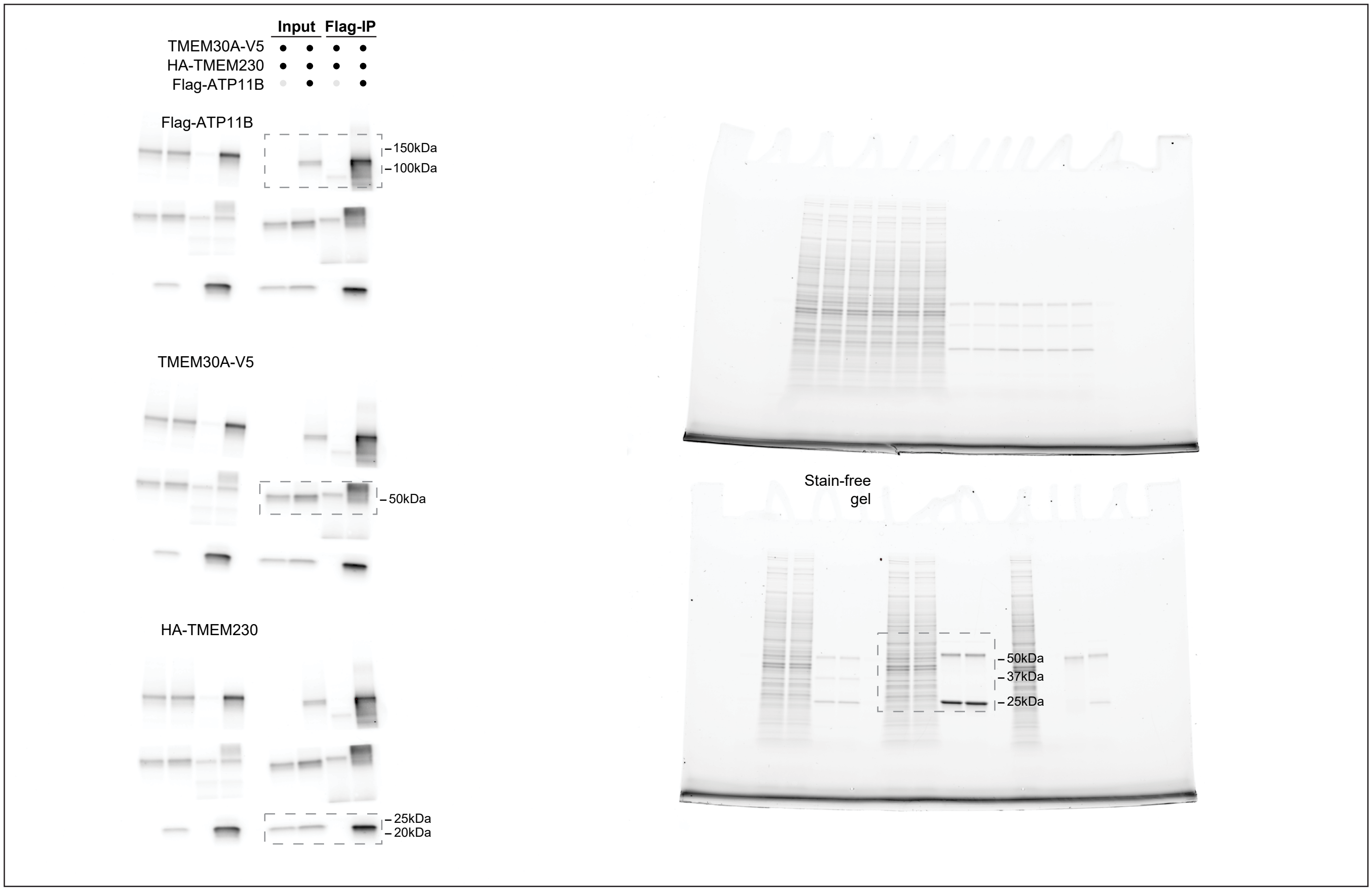

Figure 3h

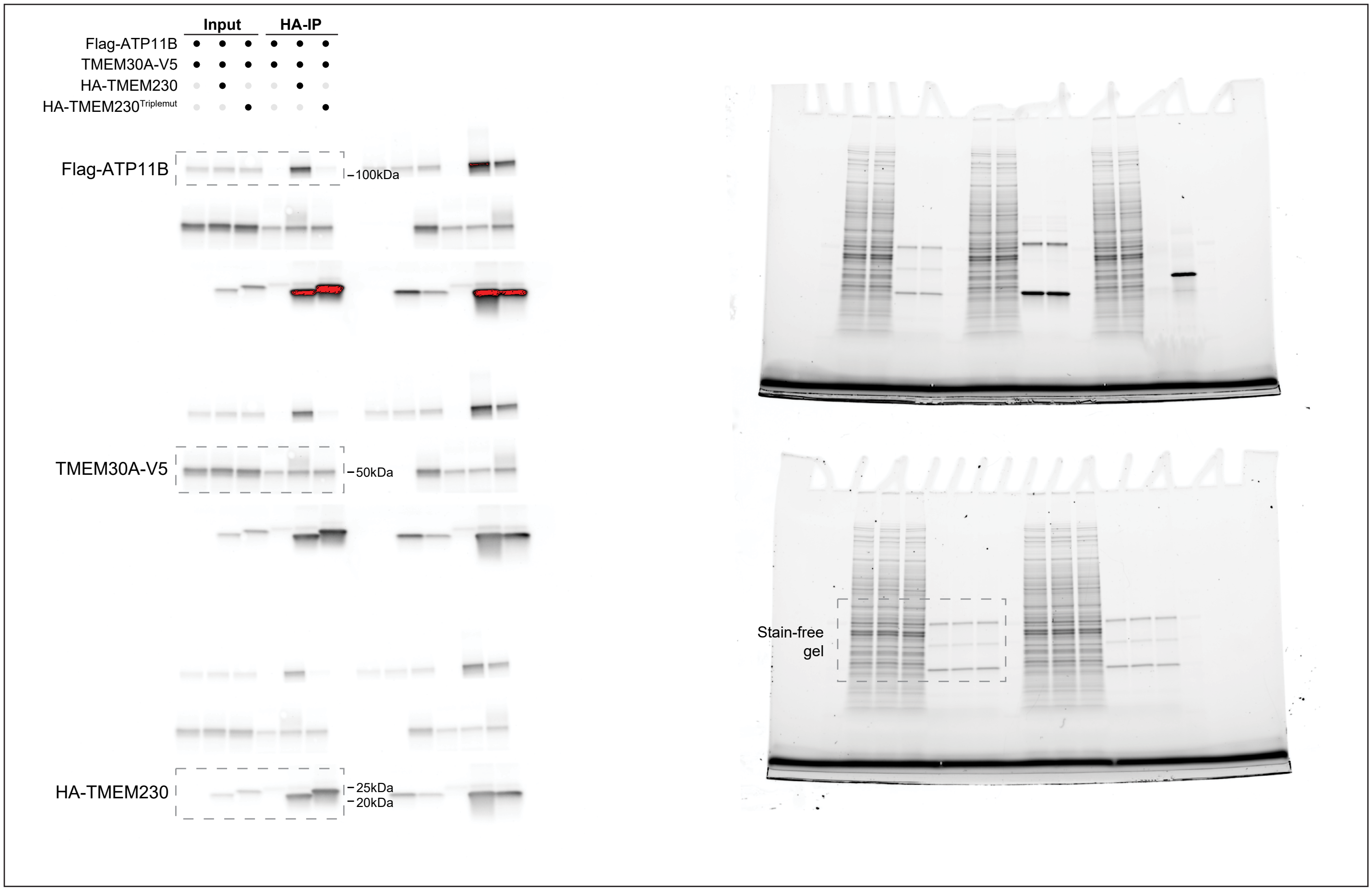

Extended Data Figure 5c

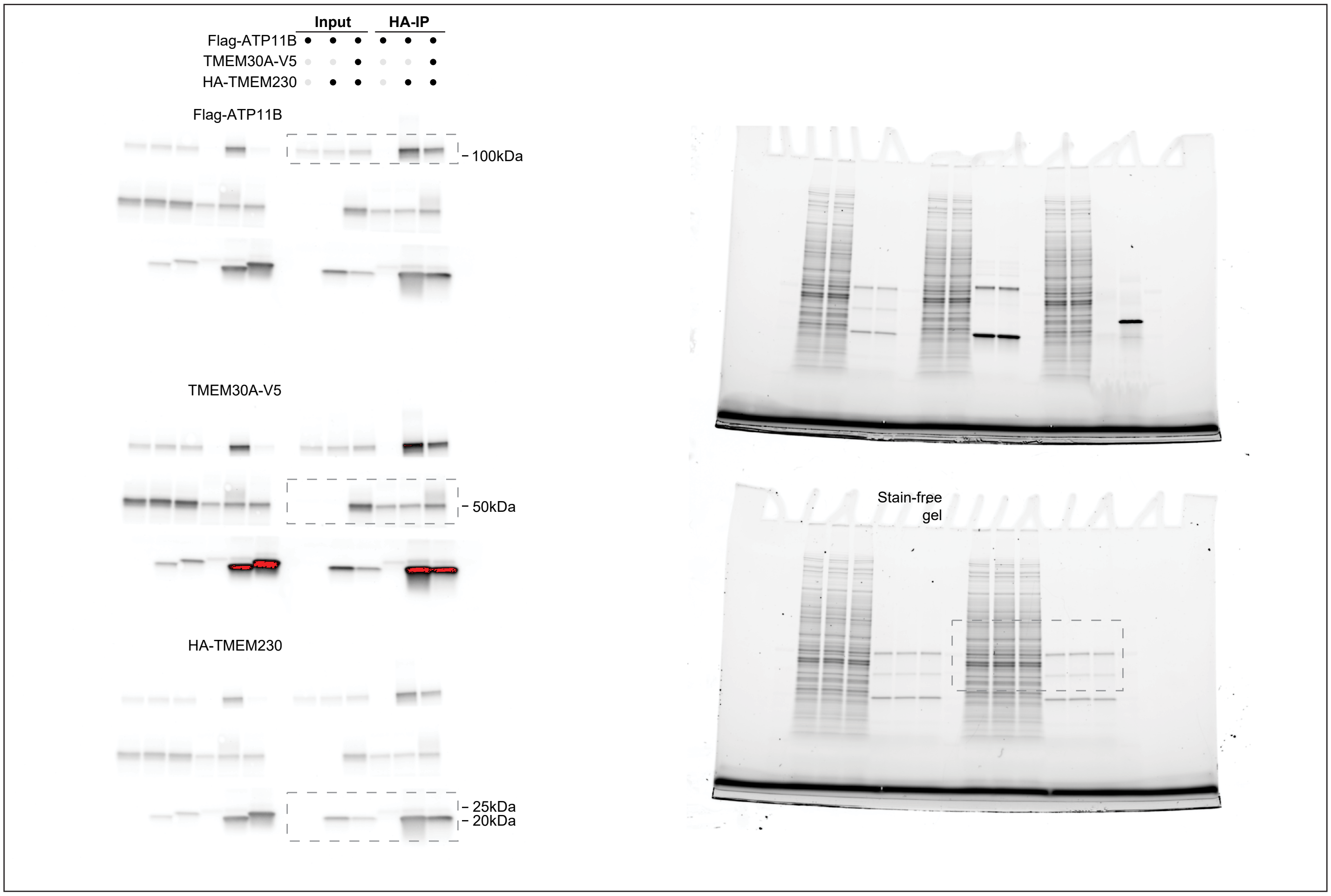

Extended Data Figure 5e

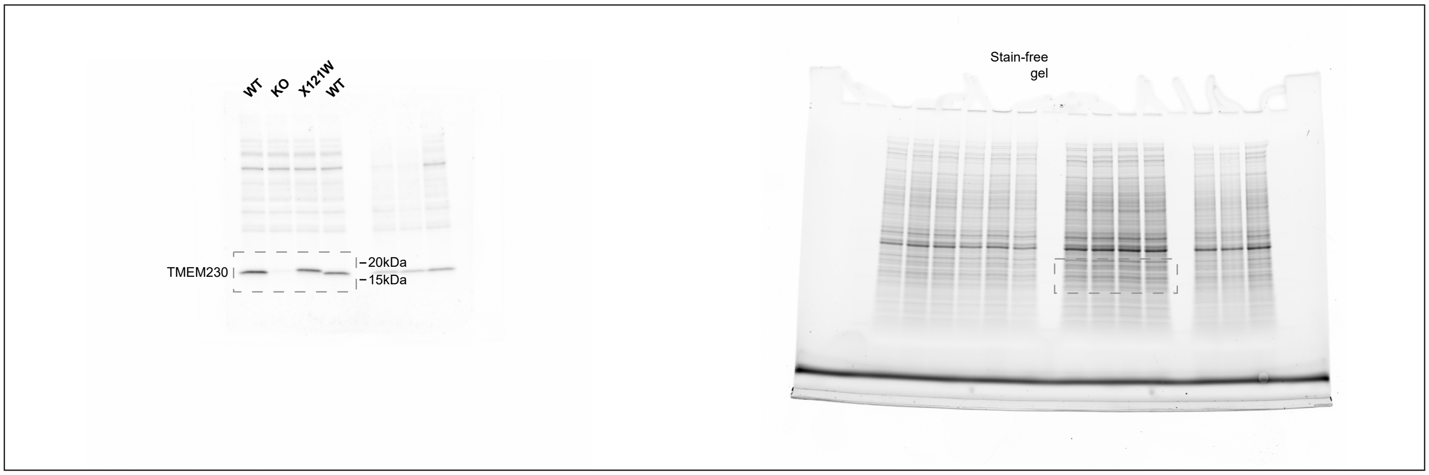

Extended Data Figure 5h

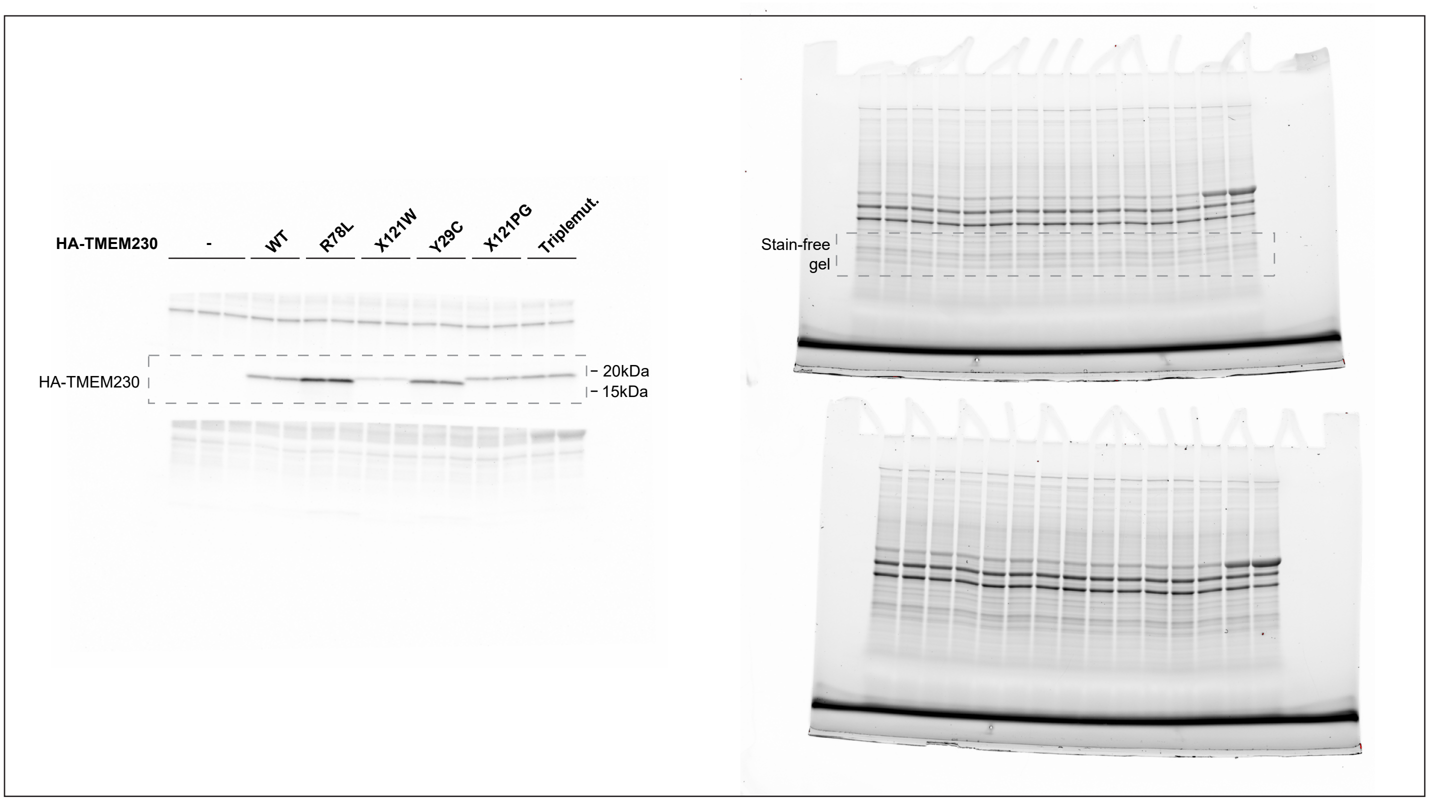

Extended Data Figure 5m

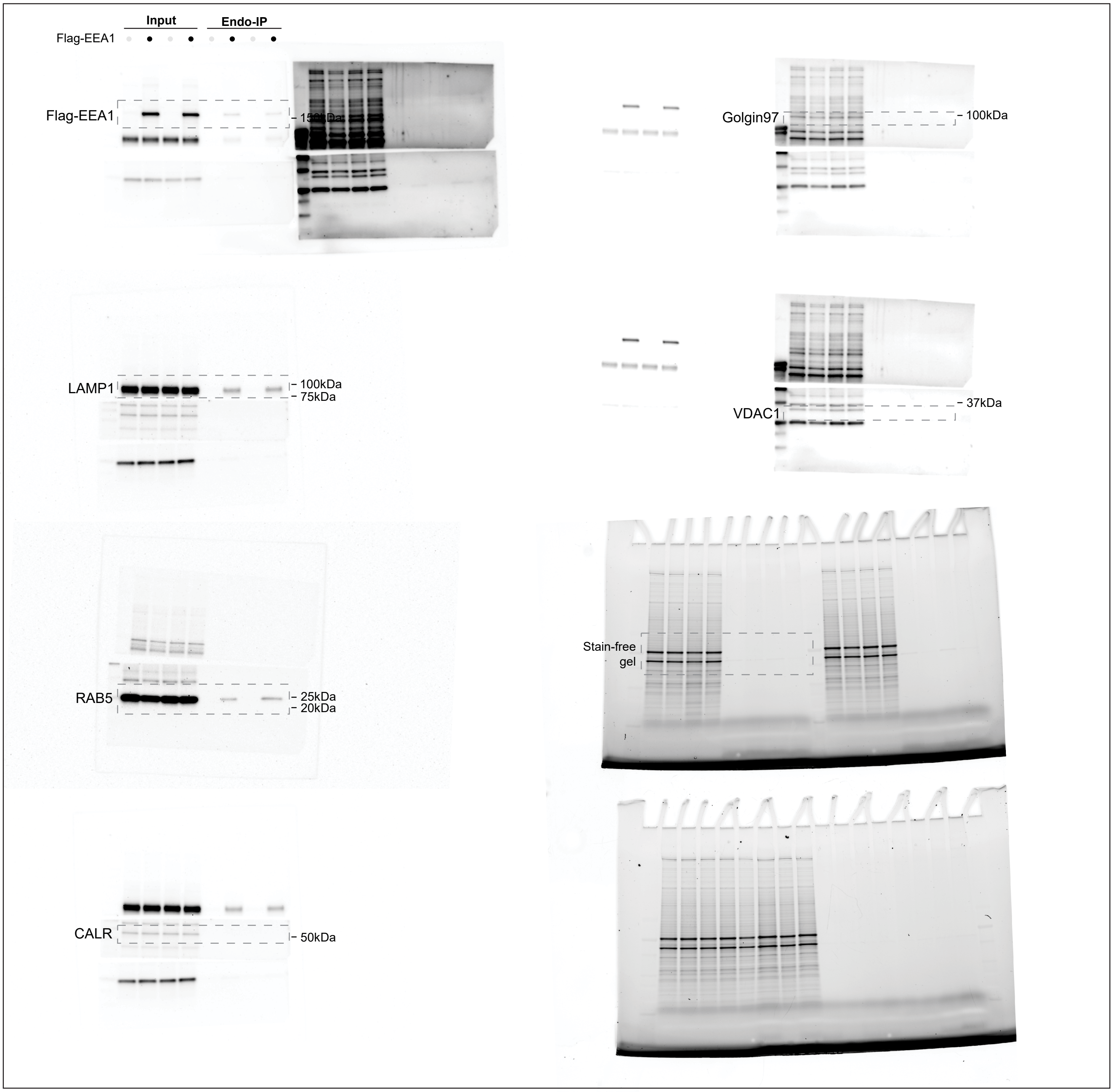

Extended Data Figure 7g

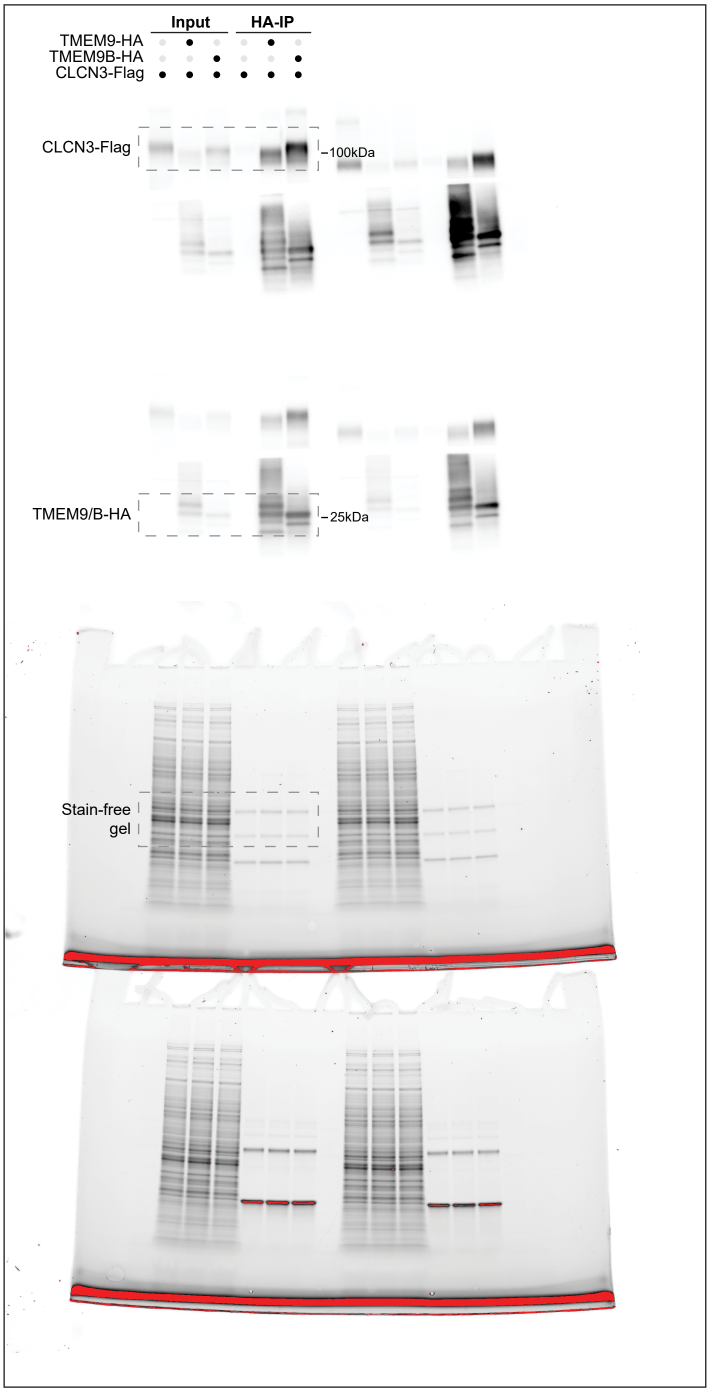

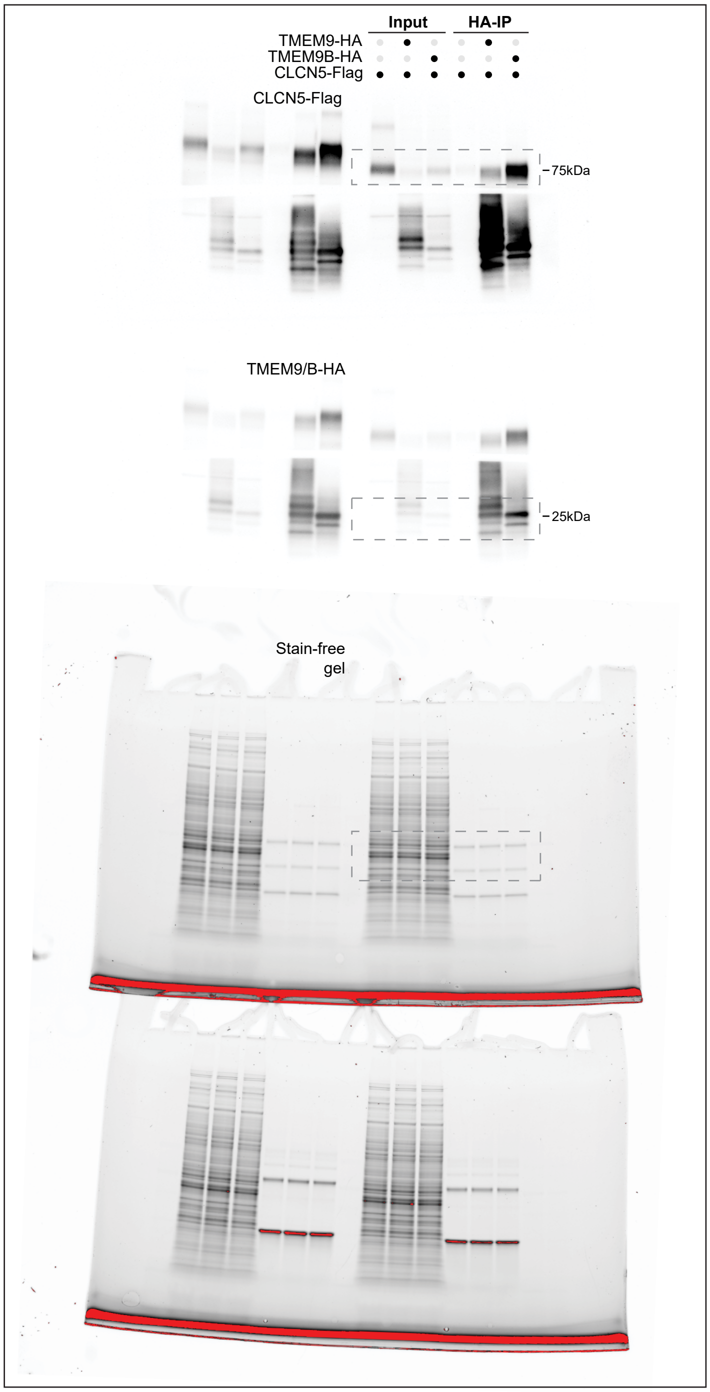

Extended Data Figure 7g (cont.)

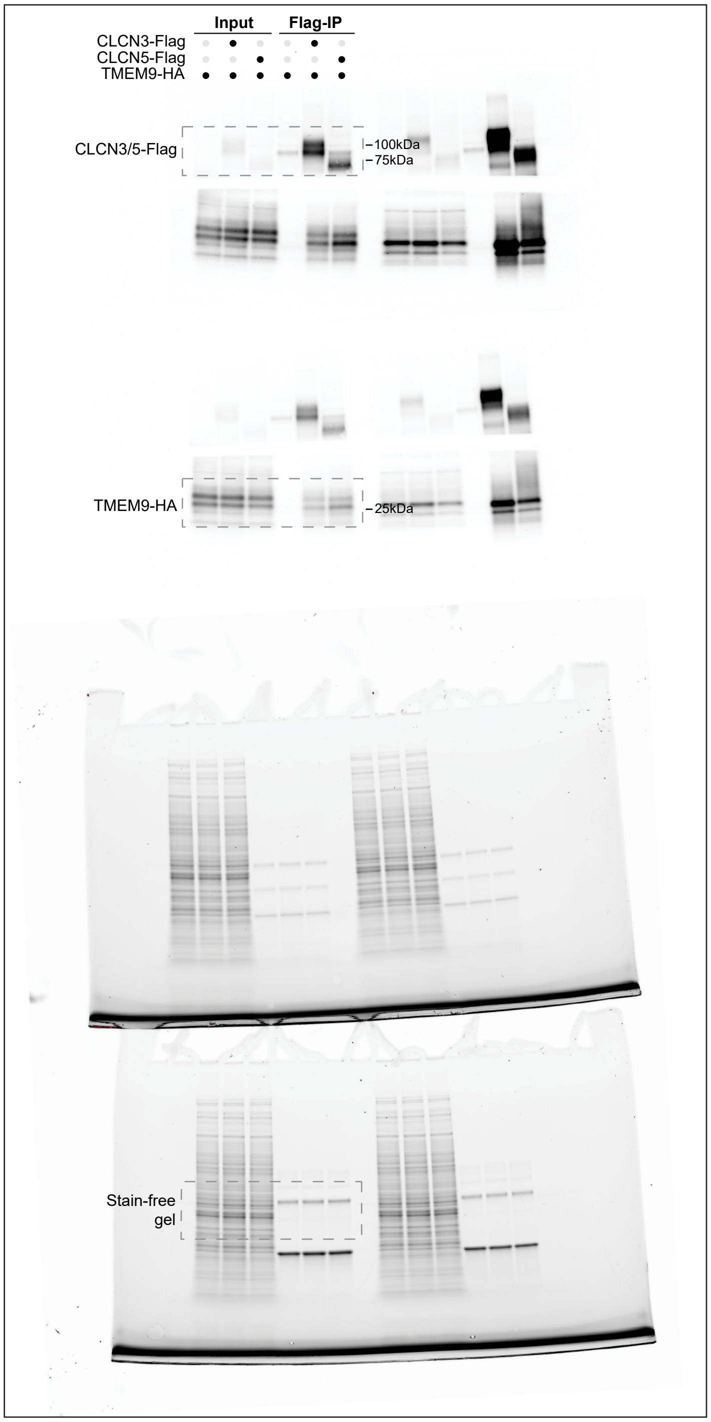

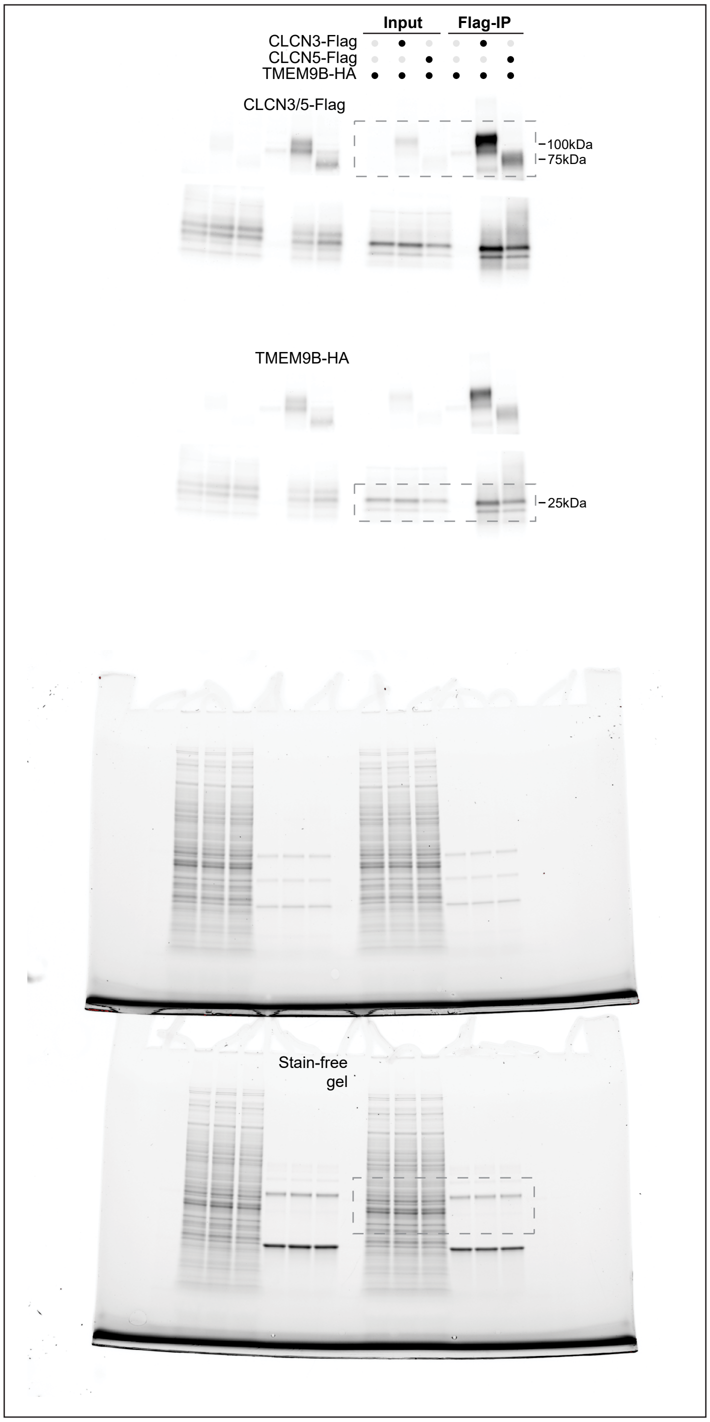

Extended Data Figure 8c

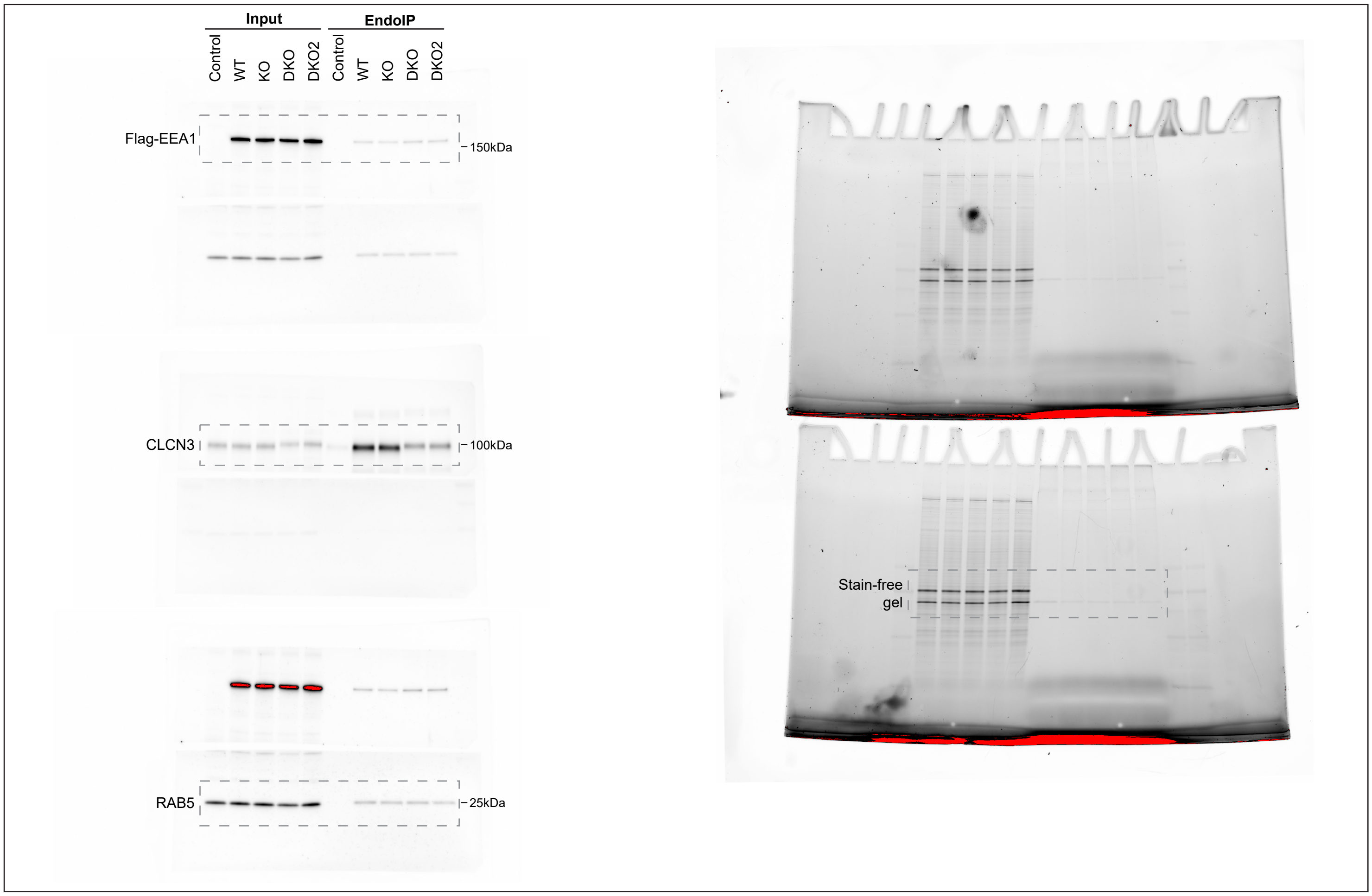

Supplement: Supplementary file 1 — Uncropped images for immunoblots and gels associated with all figures. [file 41586_2025_9059_MOESM1_ESM.pdf]
