# Supplementary material for: EndoMAP.v1 charts the structural landscape of human early endosome complexes
Source: Nature. 2025 May 28;643(8070):252–61. doi: 10.1038/s41586-025-09059-y (PMC12222028; doi:10.1038/s41586-025-09059-y)
Supplement: Supplementary file 2 — Reporting Summary [file 41586_2025_9059_MOESM2_ESM.pdf]

nature portfolio

Corresponding author(s): J. Wade Harper

Last updated by author(s): Mar 30, 2025

Reporting Summary

Nature Portfolio wishes to improve the reproducibility of the work that we publish. This form provides structure for consistency and transparency in reporting. For further information on Nature Portfolio policies, see our Editorial Policies and the Editorial Policy Checklist.

Statistics

For all statistical analyses, confirm that the following items are present in the figure legend, table legend, main text, or Methods section.

n/a	Confirmed	
☐	☒	The exact sample size (*n*) for each experimental group/condition, given as a discrete number and unit of measurement
☐	☒	A statement on whether measurements were taken from distinct samples or whether the same sample was measured repeatedly
☐	☒	The statistical test(s) used AND whether they are one- or two-sided<br>*Only common tests should be described solely by name; describe more complex techniques in the Methods section.*
☒	☐	A description of all covariates tested
☐	☒	A description of any assumptions or corrections, such as tests of normality and adjustment for multiple comparisons
☐	☒	A full description of the statistical parameters including central tendency (e.g. means) or other basic estimates (e.g. regression coefficient) AND variation (e.g. standard deviation) or associated estimates of uncertainty (e.g. confidence intervals)
☐	☒	For null hypothesis testing, the test statistic (e.g. *F*, *t*, *r*) with confidence intervals, effect sizes, degrees of freedom and *P* value noted<br>*Give P values as exact values whenever suitable.*
☒	☐	For Bayesian analysis, information on the choice of priors and Markov chain Monte Carlo settings
☒	☐	For hierarchical and complex designs, identification of the appropriate level for tests and full reporting of outcomes
☒	☐	Estimates of effect sizes (e.g. Cohen's *d*, Pearson's *r*), indicating how they were calculated

Our web collection on statistics for biologists contains articles on many of the points above.

Software and code

Policy information about availability of computer code

Data collection	Orbitrap Eclipse Tribrid Mass Spectrometer (RRID:SCR_020559) - Thermo Fisher Scientific<br>Orbitrap Fusion Lumos Tribrid Mass Spectrometer (RRID:SCR_020562) - Thermo Fisher Scientific<br>Orbitrap Exploris 480 Mass Spectrometer (RRID:SCR_022215) - Thermo Fisher Scientific<br>BioRad ChemiDoc Imaging System<br>Yokogawa CSU-X1 spinning disk confocal on a Nikon Eclipse Ti-E motorized microscope and a Plan Apochromat 100×/1.45 N.A oil-objective lens<br>Hamamatsu ORCA-Fusion BT CMOS camera (6.5 µm2 photodiode, 16-bit)<br>Illumina MiSeq (RRID:SCR_016379)<br>Sony Biotechnology SH800S Cell Sorter (RRID:SCR_018066)
Data analysis	XlinkX module implemented in Proteome Discoverer (version 2.5.0.400)<br>Comet (2019.01) (PMID: 23148064)<br>NIS-Elements image acquisition software (5.21.03 Build 1489)<br>Scout v.1.6.2; https://github.com/diogobor/Scout/releases/tag/1.6.2<br>AlphaLink: Integrating crosslinking MS data into OpenFold (V1.0); https://github.com/lhatsk/AlphaLink<br>JACoP plugin for ImageJ/FiJi (https://imagej.net/Fiji)<br>BioRad Image Lab software (version 6.1.0)<br>MSConvert (3.0.22317) (PMID: 23051804)<br>MSstats (PMID: 36622173)<br>DIA-NN (version 1.8) (PMID: 31768060)<br>MS-DAP (PMID: 36541440)

PCprophet (PMID: 33859439)
SECAT (PMID: 37182203)
g:Profiler (PMID: 27098042)
Cytoscape v3.10.1
ColabFold v 1.5.2 (PMID: 35637307)
HADDOCK2.4 web server (PMID: 38886530)
PyMOL 2.6.0 (https://www.pymol.org/)
Rstudio (2023.06.0 Build 421) + R(4.2.1)
R package: ggplot2 (3.5.1)
R package: RColorBrewer (1.1.3)
R package: ggrepel (0.9.5)
R package: dplyr (1.1.4)
R package: FactoMineR (2.11)
R package: pheatmap (1.0.12)
R package: factoextra (1.0.7)
R package: pROC (1.18.5)
R package: reshape2 (1.4.4)
R package: igraph (1.3.5)
R package: tidyr (1.3.1)
R package: lme4 (1.1.13.5)
R package: ggsignif (0.6.4)
R package: viridis (0.6.5)
R package: BiomaRt (2.54.1)
R package: circlize (0.4.16)
R package: DOSE (3.24.2)
R package: clusterProfiler (4.6.2)
Adobe Illustrator (26.5)
Code generated here: (https://github.com/harperlaboratory/EndoMAP)

For manuscripts utilizing custom algorithms or software that are central to the research but not yet described in published literature, software must be made available to editors and reviewers. We strongly encourage code deposition in a community repository (e.g. GitHub). See the Nature Portfolio guidelines for submitting code & software for further information.

Data

Policy information about availability of data

All manuscripts must include a data availability statement. This statement should provide the following information, where applicable:

- Accession codes, unique identifiers, or web links for publicly available datasets
- A description of any restrictions on data availability
- For clinical datasets or third party data, please ensure that the statement adheres to our policy

All the mass spectrometry proteomics data (289 .RAW files) have been deposited to the ProteomeXchange Consortium via the PRIDE repository (http://www.proteomexchange.org/; project accession: PXD054684, PXD054728, PXD059547 and PXD054765). The data, code, protocols, and key lab materials used and generated in this study are listed in a Key Resource Table alongside their persistent identifiers at Zenodo (10.5281/zenodo.14180546).
All AF-M and AlphaLink2 predictions can be downloaded from https://endomap.hms.harvard.edu (RRID:SCR_026690) and have also been deposited at Zenodo (10.5281/zenodo.14447604 and 10.5281/zenodo.14632928). Input/output files used for modeling mTORC1-Ragulator-VATPase complex using HADDOCK2.4 have been deposited in Zenodo (10.5281/zenodo.14679635). Raw imaging data has been deposited in Zenodo (10.5281/zenodo.14826176 and 10.5281/zenodo.14828025).
We used canonical protein entries from the Human reference proteome database in our study (UniProt Swiss-Prot release 2021-11, 2022-05 and 2024-01; https://ftp.uniprot.org/pub/databases/uniprot/previous_major_releases/).
Full version of all gels and blots are provided in SI Fig. 1. Source data is provided in SI when not already available in Supplementary Tables.

Research involving human participants, their data, or biological material

Policy information about studies with human participants or human data. See also policy information about sex, gender (identity/presentation), and sexual orientation and race, ethnicity and racism.

Reporting on sex and gender	*Use the terms sex (biological attribute) and gender (shaped by social and cultural circumstances) carefully in order to avoid confusing both terms. Indicate if findings apply to only one sex or gender; describe whether sex and gender were considered in study design; whether sex and/or gender was determined based on self-reporting or assigned and methods used. Provide in the source data disaggregated sex and gender data, where this information has been collected, and if consent has been obtained for sharing of individual-level data; provide overall numbers in this Reporting Summary. Please state if this information has not been collected. Report sex- and gender-based analyses where performed, justify reasons for lack of sex- and gender-based analysis.*
Reporting on race, ethnicity, or other socially relevant groupings	*Please specify the socially constructed or socially relevant categorization variable(s) used in your manuscript and explain why they were used. Please note that such variables should not be used as proxies for other socially constructed/relevant variables (for example, race or ethnicity should not be used as a proxy for socioeconomic status). Provide clear definitions of the relevant terms used, how they were provided (by the participants/respondents, the researchers, or third parties), and the method(s) used to classify people into the different categories (e.g. self-report, census or administrative data, social media data, etc.)*

Please provide details about how you controlled for confounding variables in your analyses.

Population characteristics	*Describe the covariate-relevant population characteristics of the human research participants (e.g. age, genotypic information, past and current diagnosis and treatment categories). If you filled out the behavioural & social sciences study design questions and have nothing to add here, write "See above."*
Recruitment	*Describe how participants were recruited. Outline any potential self-selection bias or other biases that may be present and how these are likely to impact results.*
Ethics oversight	*Identify the organization(s) that approved the study protocol.*

Note that full information on the approval of the study protocol must also be provided in the manuscript.

Field-specific reporting

Please select the one below that is the best fit for your research. If you are not sure, read the appropriate sections before making your selection.

☒ Life sciences ☐ Behavioural & social sciences ☐ Ecological, evolutionary & environmental sciences

For a reference copy of the document with all sections, see nature.com/documents/nr-reporting-summary-flat.pdf

Life sciences study design

All studies must disclose on these points even when the disclosure is negative.

Sample size	No sample-size calculation was performed. The sample size for each experiment is indicated in figures, legends and methods section. Number of biological replicates for TMT-based proteomic experiments was determined given the limitation of the available TMT channels. Extensive work in the field has shown that this approach provides the necessary statistical significance. E.g. An et al Systematic quantitative analysis of ribosome inventory during nutrient stress. Ordureau A, Kraus F, Zhang J, An H, Park S, Ahfeldt T, Paulo JA, Harper JW. Temporal proteomics during neurogenesis reveals large-scale proteome and organelle remodeling via selective autophagy. Mol Cell. 2021 Dec 16;81(24):5082-5098.e11. doi: 10.1016/j.molcel.2021.10.001.
Data exclusions	No data were excluded from the analyses.
Replication	All attempts of replication were successful. Most experiments were replicated three times and the number of biological replicates is provided for each experiment in the figure legend and Statistics and Reproducibility methods section.
Randomization	Most experiments do not require randomization since either no group comparison was performed or samples were analyzed simultaneously (TMT-based proteomics). For DIA-based proteomics, the experiments were performed in randomized blocks.
Blinding	Blinding was not relevant in this study since either no group comparison was performed or samples were analyzed simultaneously (TMT-based proteomics).

Reporting for specific materials, systems and methods

We require information from authors about some types of materials, experimental systems and methods used in many studies. Here, indicate whether each material, system or method listed is relevant to your study. If you are not sure if a list item applies to your research, read the appropriate section before selecting a response.

Materials & experimental systems

n/a	Involved in the study	
☐	☒	Antibodies
☐	☒	Eukaryotic cell lines
☒	☐	Palaeontology and archaeology
☒	☐	Animals and other organisms
☒	☐	Clinical data
☒	☐	Dual use research of concern
☒	☐	Plants

Methods

n/a	Involved in the study	
☒	☐	ChIP-seq
☒	☐	Flow cytometry
☒	☐	MRI-based neuroimaging

Antibodies

Antibodies used	FLAG (Sigma-Aldrich, F1804; RRID:AB_262044)Dilution: 1:1,000 for immunoblotting, 1:400 for immunofluorescence<br>HA (Cell Signaling Technology, 3724; RRID:AB_353989)Dilution: 1:1,000 for immunoblotting, 1:400 for immunofluorescence<br>V5 (Invitrogen, 14-6796-82; RRID:AB_10718239)Dilution: 1:1,000 for immunoblotting, 1:400 for immunofluorescence<br>TMEM230 (Origene, TA504888; RRID:AB_2622676)Dilution: 1:1,000 for immunoblotting, 1:400 for immunofluorescence<br>LAMP1 (Cell Signaling Technology, D2D11; RRID:AB_2927691)Dilution: 1:1,000 for immunoblotting, 1:400 for immunofluorescence<br>RAB5 (Cell Signaling Technology, C8B1; RRID:AB_2300649)Dilution: 1:1,000 for immunoblotting, 1:400 for immunofluorescence

CLR (ProteinTech, 10292-1-AP; RRID:AB_2314206)Dilution: 1:1,000 for immunoblotting, 1:400 for immunofluorescence
Golgin 97 (ProteinTech, 12640-1-AP; RRID:AB_2115315)Dilution: 1:1,000 for immunoblotting, 1:400 for immunofluorescence
VDAC1 (ProteinTech, 55259-1-AP; RRID:AB_10837225)Dilution: 1:1,000 for immunoblotting, 1:400 for immunofluorescence
CLCN3 (Cell Signaling Technology, 13359S; RRID:AB_2486248)Dilution: 1:1,000 for immunoblotting, 1:400 for immunofluorescence
GFP (Thermo Scientific, a10262; RRID:AB_770014)Dilution: 1:1,000 for immunoblotting, 1:400 for immunofluorescence
mCherry (Thermo Scientific, M11217; RRID:AB_2536611)Dilution: 1:1,000 for immunoblotting, 1:400 for immunofluorescence
EEA1 (Cell Signaling Technology, C45B10; RRID:AB_2221630)Dilution: 1:1,000 for immunoblotting, 1:400 for immunofluorescence
Anti-rabbit immunoglobulin-G (IgG) horse radish peroxidase (HRP) conjugate (BioRad, 1706515; RRID:AB_11125142)Dilution: 1:1,000 for immunoblotting, 1:400 for immunofluorescence
Anti-mouse IgG HRP conjugate (BioRad, 1706516; RRID:AB_11125547)Dilution: 1:1,000 for immunoblotting, 1:400 for immunofluorescence
Goat anti-Chicken IgY (H+L), Alexa Fluor 488 (Thermo Scientific, A-11039; RRID:AB_2534096)Dilution: 1:1,000 for immunoblotting, 1:400 for immunofluorescence
Goat anti-Rat IgG (H+L) Cross-Adsorbed, Alexa Fluor 555 (Thermo Scientific, A-21434; RRID:AB_2535855)Dilution: 1:1,000 for immunoblotting, 1:400 for immunofluorescence
Goat anti-Rabbit IgG (H+L) Cross-Adsorbed, Alexa Fluor 647 (Thermo Scientific, A-21244; RRID:AB_2535812)Dilution: 1:1,000 for immunoblotting, 1:400 for immunofluorescence

Validation

FLAG, HA, V5 and TMEM230 were validated by immunoblotting using cells with the target gene tagged or deleted by CRISPR (see Figure 3d, 3h, Extended Data Figure 5c, 5e, 5h, 5m, 7g, 8c). Validation and publications for other antibodies were provided by the suppliers.
FLAG antibody was validated for highly sensitive and specific detection of FLAG fusion proteins by immunoblotting (WB), immunoprecipitation (IP), immunohistochemisty (IHC), immunofluorescence (IF) and immunocyotchemistry (ICC) (https://www.sigmaaldrich.com/US/en/product/sigma/f1804).
HA antibody was validated for WB, IHC, IP, IF, Flow cytometry (FC) and ChIP (https://www.cellsignal.com/products/primary-antibodies/ha-tag-c29f4-rabbit-mab/3724).
V5 antibody application was validated for recombinant proteins containing a V5 epitope tag in WB and ICC/IF (https://www.thermofisher.com/antibody/product/V5-Tag-Antibody-clone-TCM5-Monoclonal/14-6796-82).
TMEM230 was additionally tested by the vendor in WB, IF and FC (Origene, TA504888).
LAMP1 antibody was tested by the vendor and a large body of literature for WB, IP, IHC, IF and FC (https://www.cellsignal.com/products/primary-antibodies/lamp1-d2d11-xp-rabbit-mab/9091).
RAB5 antibody detects endogenous levels of total Rab5A, Rab5B and Rab5C protein in WB and ICC (https://www.cellsignal.com/products/primary-antibodies/rab5-c8b1-rabbit-mab/3547).
CLR antibody was tested for application in WB, ICC and FC and validated in several publication with KD/KO controls (https://www.ptglab.com/products/CALR-Antibody-10292-1-AP.htm).
Golgin 97 antibody was tested by vendor for application in WB, ICC, IF and FC (https://www.ptglab.com/products/GOLGA1-Antibody-12640-1-AP.htm).
VDAC1 antibody was tested for application in WB, IHC, IF and FC and validated in several publication with KD/KO controls (https://www.ptglab.com/products/VDAC1-Antibody-55259-1-AP.htm).
CLCN3 antibody recognizes endogenous levels of total CLCN3 protein in WB, IP and IF (https://www.cellsignal.com/products/primary-antibodies/clcn3-d8y5q-rabbit-mab/13359).
GFP antibody specificity was demonstrated by detection of different targets fused to GFP tag in transiently transfected lysates, and tested for application in WB, ICC and IF (https://www.thermofisher.com/antibody/product/GFP-Antibody-Polyclonal/A10262).
mCherry antibody was extensively validated by the vendor and publications for WB, IHC, IF, FC and IP (https://www.thermofisher.com/antibody/product/mCherry-Antibody-clone-16D7-Monoclonal/M11217).
EEA1 antibody detects endogenous levels of total EEA1 protein and is validated for WB, ICC, IF and IP (https://www.cellsignal.com/products/primary-antibodies/eea1-c45b10-rabbit-mab/3288).

Eukaryotic cell lines

Policy information about cell lines and Sex and Gender in Research

Cell line source(s)

HEK293 and HEK293T cells (RRID:CVCL_0045). From ATCC (#CRL-3216 and #CRL-1573).
HEK293EL (RRID:CVCL_C0I7). Generated by us in a previous study (PMID: 36245040).
SUM159PT (RRID:CVCL_5423). Gift from Tobias Walther (Memorial Sloan Kettering). Bioivt HUMANSUM-0003006
H9 hESC Wicell WA9 (RRID:CVCL_9773).
H9 AAVS1-TRE3G-NGN2 3xFLAG-EEA1 (RRID:CVCL_D1KV). Generated by us in a previous study (PMID: 39636867).

Authentication

ATCC preforms quality testing to ensure authentication of the HEK293T cell line using Short Tandem Repeat Analysis (STR).
H9 ES cells (from WiCell) are authenticated by WiCell using G-band karyotyping and Short Tandem Repeat Analysis (STR).
Genetically edited H9 hESCs were confirmed by karyotyping. Successful conversion of stem cells to NGN2 induced neurons was confirmed by mass spectrometry and microscopy analysis.

Mycoplasma contamination

All cell lines tested free of mycoplasma using Mycoplasma Plus PCR assay kit (Agilent).

Commonly misidentified lines
(See ICLAC register)

none

Plants

Seed stocks	*Report on the source of all seed stocks or other plant material used. If applicable, state the seed stock centre and catalogue number. If plant specimens were collected from the field, describe the collection location, date and sampling procedures.*
Novel plant genotypes	*Describe the methods by which all novel plant genotypes were produced. This includes those generated by transgenic approaches, gene editing, chemical/radiation-based mutagenesis and hybridization. For transgenic lines, describe the transformation method, the number of independent lines analyzed and the generation upon which experiments were performed. For gene-edited lines, describe the editor used, the endogenous sequence targeted for editing, the targeting guide RNA sequence (if applicable) and how the editor was applied.*
Authentication	*Describe any authentication procedures for each seed stock used or novel genotype generated. Describe any experiments used to assess the effect of a mutation and, where applicable, how potential secondary effects (e.g. second site T-DNA insertions, mosiacism, off-target gene editing) were examined.*
