# Supplementary material for: EndoMAP.v1 charts the structural landscape of human early endosome complexes
Source: Nature. 2025 May 28;643(8070):252–61. doi: 10.1038/s41586-025-09059-y (PMC12222028; doi:10.1038/s41586-025-09059-y)
Supplement: Supplementary file 3 — Extension of our presentation of multiple facets of this paper. [file 41586_2025_9059_MOESM3_ESM.docx]

**SUPPLEMENTARY TEXT**

**EndoMAP.v1 charts the structural landscape of human early endosome complexes**

Miguel A. Gonzalez-Lozano^1,2^, Ernst W. Schmid^3^, Enya Miguel Whelan^1,2^, Yizhi Jiang^1,2,4^, Joao A. Paulo^1^, Johannes C. Walter^3,5^, and J. Wade Harper^1,2^

^1^Department of Cell Biology, Harvard Medical School, Boston MA, USA

^2^Aligning Science Across Parkinson’s (ASAP) Collaborative Research Network, Chevy Chase, MD 20815, USA

^3^Department of Biological Chemistry and Molecular Pharmacology, Harvard Medical School, Boston MA, USA

^4^Initiative for Genome Editing and Neurodegeneration, Department of Cell Biology, Harvard Medical School, Boston MA, USA

^5^Howard Hughes Medical Institute, Boston, MA, USA

Corresponding author: wade_harper@hms.harvard.edu

**SUPPLEMENTARY TEXT**

In this **Supplementary Text** document, we extend our presentation of multiple facets of this paper.

**Endosomal Proteome scoring method**

Given the dynamic nature of the endosomal system, we first wanted to define and characterize the endosomal proteome, including resident and transient endosomal proteins such as cargo, based on published experimental data. We analyzed the proteins identified in 16 studies that involved a variety of purification approaches and cell types (**Extended Data Fig. 1a, Supplementary Table 1**). Multiple Correspondence Analysis (MCA) revealed extensive diversity across the datasets that segregated, in part, based on purification method (**Extended Data Fig. 1b**). Endo-IP^19^ and correlation-based gradient fractionation^24^ approaches recovered the largest number of well-known endosomal proteins (111 and 107, respectively; **Extended Data Fig. 1a,** see **METHODS**). Nevertheless, the presence of individual proteins in multiple datasets was predictive of endosomal localization, including proteins transiently localized to endosomes throughout the dynamics of endosomal function and maturation to lysosomes (**Extended Data Fig. 1c**). In addition, the presence of specific endosomal proteins in multiple datasets correlated with higher protein abundance in samples purified by Endo-IP^19^ (**Extended Data Fig. 1d**). These metrics were compared as predictors of endosomal localization, including: **1)** the number of independent datasets in which each protein was identified, **2)** the protein abundance in Endo-IP, and **3)** the number of interactions with well-known endosomal proteins (**Fig. 1b**). Predictors were evaluated using a manually curated set of well-known endosomal proteins as reference (**Supplementary Table 1,** see **METHODS**). The combination of all three predictors best captured many well-characterized endosomal proteins with high confidence (**Fig. 1b**). Moreover, this combined score identified numerous predicted endosomal proteins that appear to be understudied based on large-scale protein interaction studies (BioPlex and OpenCell)^28,112^ and published literature (**Extended Data Fig. 1e-g**). In total, this analysis identified 522 endosomal proteins (**Supplementary Table 1**), including known and predicted endosomal proteins that were the target for further characterization with our complexomics pipeline as “reference” endosomal proteome (**Fig. 1a**).

**Systematic Analysis of EndoMAP.v1 using AlphaLink2**

AF-M provides a means by which to predict protein structures as well as interfaces between protein pairs or higher order complexes. Previous studies have sought to integrate cross-linking distance constraints into AF-M predictions using AlphaLink2^21^. However, the extent to which AlphaLink2 could be broadly used to evaluate protein interactions in high-throughput studies is currently unclear.

Given the scale of our data, we sought to compare AlphaLink2, directed by intra and inter-protein DSSO cross-links, with AF-M predictions. At a global level, pairwise predictions with strong AF-M ipTM scores were typically largely unchanged (**Fig. 2h,** **Extended Data Fig. 4c**). For instance, ipTM scores for AF-M and AlphaLink2 were comparable for the interaction of ARL8B with the N-terminal RUN domain of RUFY2 (0.86 and 0.81, respectively) (**Extended Data Fig. 4e**). The cross-link remained exceeding the acceptable distance, with 43Å and 49.5Å for AF-M and AlphaLink2, respectively, likely due to the flexible linker between the RUN domain and coiled-coil region of RUFY2. Other examples include both TMEM230-ATP11B and TMEM9-CLCN3, as described in the main text. In both cases, the ipTM scores were comparable and the position of the cross-links similar, although not identical, with AlphaLink2 resulting in longer cross-link distances for both TMEM230-ATP11B and TMEM9-CLCN3 [10.6Å to 17.6Å and 33.0Å to 39.0Å, respectively] (**Extended Data Fig. 5a and 7b**). However, there are numerous cases where scores differed between AF-M and AlphaLink2 (**Extended Data Fig. 4f-i**). One example of a prediction whose score and structural prediction appears to be negatively affected by inclusion of cross-linking data is RAB7A-VPS35 (**Extended Data Fig. 4f**). AF-M places RAB7 on the concave surface of the VPS35 solenoid (ipTM = 0.83), with a DSSO cross-link between VPS35 and the unstructured region of RAB7A’s C-terminus. Additionally, there is a DSSO cross-link between RAB7A and VPS29, as well as a DMTMM cross-link between RAB7A and VPS35 that conform to distance constraints in the context of the RAB7A-VPS35-VPS29 trimer (**Fig. 5b**). In contrast, AlphaLink2 places RAB7A on the convex surface of the VPS35 solenoid, with a shorter DSSO cross-link distance (35.9Å versus 23.2Å), a reduced ipTM (0.83 to 0.64), and a much longer DMTMM cross-link distance (11.1Å versus 56.6Å) (**Extended Data Fig. 4f**). The position of RAB7A in the AlphaLink2 prediction would also have an unacceptably long cross-link to VPS29. Thus, VPS35-RAB7A is an example of interaction with strong ipTM score for AF-M prediction that appears to be negatively impacted by integrating cross-linking constraints in AlphaLink2.

We also observed instances where ipTM scores decreased without altering cross-link distances. A case in point is the BLOC1S1-BLOC1S2 pair. The ipTM scores decreased from 0.49 with AF-M to 0.23 with Alphalink2, but the cross-link distance was very similar (20.4Å versus 21.5Å) (**Extended Data Fig. 4g**). The difference in ipTM likely reflects the fact that the helical domain of BLOC1S1 folds back on itself with AF-M but remains extended with AlphaLink2. The register of the helical packing between BLOC1S1 and BLOC1S2 is also distinct for the two predictions, as indicated in the structural overlay (**Extended Data Fig. 4g**).

In contrast, pairwise predictions with low AF-M scores (ipTM<0.3) frequently showed higher scores with AlphaLink2 (**Fig. 2h**). A case in point is the prediction of VAMP3 with either SCAMP1 or SCAMP3 (**Extended Data Fig. 4h,i**). AF-M produces a prediction for VAMP3-SCAMP1 (ipTM = 0.22) where the cytosolic helical domain of SCAMP1 is cross-linked with the cytosolic region of VAMP3 in a position that is compatible with SCAMP1’s multi-pass TM region being in the membrane (**Extended Data Fig. 4h**). AlphaLink2 produces a higher score (ipTM = 0.4) with the cross-link at a similar distance (15.8Å versus 14.9Å), but with the helical interactions in a distinct orientation relative to the AF-M prediction (**Extended Data Fig. 4h**). The effect of including cross-links is much more pronounced in the context of SCAMP3, where AF-M inverts the orientation of SCAMP3’s cytosolic helical region relative to the membrane spanning segments, resulting in cross-link distances of >100Å (ipTM = 0.22) (**Extended Data Fig. 4i**). In contrast, AlphaLink2 places the cytosolic helical domain of SCAMP3 in an orientation compatible with the membrane topology, the cross-link within the distance constraint (12.0Å), and a higher ipTM score (0.38) (**Extended Data Fig. 4i**).

Overall, our results indicate that AF-M predictions with strong ipTM scores are less impacted by inclusion of cross-link constraints into the predictions, while protein pairs with low scores typically showed larger improvement with AlphaLink2. Nevertheless, direct experiments will be required to understand the extent to which improved scores more closely approximate actual structures. All AF-M and AlphaLink2 predictions, with cross-link distances, are available at <https://endomap.hms.harvard.edu> and Zenodo (10.5281/zenodo.14447604 and 10.5281/zenodo.14632928).

**Candidate Disease Variants Near Protein Interaction Interfaces**

Disease variants in proteins often involve either residues critical for folding, the disruption of which can promote misfolding and reduced stability, or residues that participate in interactions with other proteins. The latter class includes residues that directly contact an interacting protein, or whose mutation alters the conformation of the interaction surface in a way that reduces binding affinity of a partner protein. The large-scale prediction of interaction interfaces provided an opportunity to systematically investigate the extent to which candidate disease variants may be located at or nearby subunit interfaces, potentially disrupting proper complex formation. We searched for protein coding candidate disease variants derived from Uniprot ((release 2021-11; 2024-01; RRID:SCR_002380) that were near predicted interfaces (within 2 amino acids from an interacting residue) (see **METHODS**). We identified 34 such cases involving 53 variants in endosome-related proteins (**Extended Data Fig. 6i,j, Supplementary Table 3**). Examples of variants within interfaces with pairwise ipTM scores greater than 0.5 are shown in **Extended Data Fig. 6j**. As we demonstrated for TMEM230-ATP11B, interface variants can disrupt physical interactions, suggesting that this approach may be useful for identifying interacting partners of proteins whose interactions are disrupted by specific variants.

**Building SNARE Networks**

SNARE proteins facilitate vesicle fusion and endosomal maturation. We identified cross-links defining dozens of pairwise combinations of R-SNARE, Q-SNARE, regulatory and RAB proteins (**Extended Data Fig. 9c**), allowing generation of numerous predictions following the stoichiometry of three Q-SNAREs and one R-SNARE in the core complex with supporting DSSO and DHSO/DMTMM cross-links (**Extended Data Fig. 9d-h**). Pairwise, 3-way, and tetrameric AF-M predictions of core SNARE complexes formed post-vesicle fusion structures with numerous cross-links found: **1)** between N-terminal Helical a, b, c (Habc) domains of Q-SNAREs (**Extended Data Fig. 9d-f**), **2)** soluble fusion factors, including NAPA (N-ethylmaleimide-sensitive factor attachment protein alpha) and NAPG, which have been reported to co-associate^28,112^, and **3)** HOPS tethering complex subunits including VPS16 (**Extended Data Fig. 9g,h**). Finally, we identified cross-links between SNARE components and two distinct classes of membrane-embedded proteins with extensive data supporting biochemical interactions: multi-TM SCAMP proteins and the single TM protein PTTG1IP (**Extended Data Fig. 9i-n**). SCAMP proteins are known to be involved in vesicle secretion but to our knowledge have not been reported to directly interact with SNAREs. Although pairwise AF-M scores are weak for VAMP2/3-SCAMP1/3 predictions (0.01<SPOC <0.36), there are numerous interactions seen by co-IP^112^ and cross-links in largely unstructured cytosolic regions of SCAMPs consistent with interactions on the cytosolic regions of both R- and Q-SNAREs (**Extended Data Fig. 9i,j**). Similarly, PTTG1IP, which is predicted in Uniprot to have a TM (residues 97-117), was cross-linked to both Q and R-SNAREs, and displayed multiple interactions with SNARE components at endogenous protein levels as annotated in Open Cell^112^ (**Extended Data Fig. 9k**). Pairwise AF-M predictions between PTTG1IP and VAMP3/VAMP8/VTI1B resulted in SPOC scores >0.47, with cross-links within the distance range for the pentameric complex prediction for the PTTG1IP-VAMP8 DSSO cross-link (**Extended Data Fig. 9l-n**). To our knowledge, PTTG1IP has not been previously linked with SNARE functions, although we note that it is enriched in lysosomes based on previous correlation profiling data^18^. Additionally, PTTG1IP abundance in HeLa cells is ~6% of VAMP3 (see ^18^), suggesting specialized functions.

Together, these data suggest a combinatorial interplay between Q/R-SNARE, RABs, soluble fusion/tethering factors, and new candidate TM containing proteins as possible regulators which likely coordinate endosomal maturation events.

**V-ATPase as an Interaction Hub**

Multiple cross-link supported interactions between V-ATPase and Ragulator were described in the main text, which allows the generation of a hypothetical model of a V-ATPase-Ragulator-MTORC1 “super assembly”. Additionally, several cross-links were identified between components of the BORC complex and both the V-ATPase and LAMTOR proteins, (**Extended Data** **Fig. 11a,b**), although the AF-M predictions had low SPOC score (**Supplementary Table 3**). Additionally, LAMTOR3 was found to cross-link with ARL8B, which is known to contribute to BORCs ability to position endolysosomes within cells^22^ (**Extended Data Fig. 11a**). We speculate that V-ATPase, LAMTOR, and ARL8 may form a nexus that can associate with BORC to facilitate endolysosomal trafficking via kinesin motor complexes. Interestingly, our data further validate the previously reported structure of V-ATPase in association with MEAK7^148^, a TBC and LysM Domain containing (TLDc)-domain protein reported as a modest activator of V-ATPase activity in vitro^149^, including cross-links with V1 subunits ATP6V1D and ATP6V1B2 (**Extended Data Fig. 11c-e**). We also identified cross-links between residues on the cytoplasmic domain of the V0 subunit ATP6V0A1 and multiple endolysososomal RABs (RAB33B, RAB14, RAB4A, RAB5C) (**Extended Data Fig. 11f**), all surpassing the 1% FDR threshold as determined by Scout^31^ (**Supplementary Table 2**), although with low AF-M pairwise SPOC scores. Such an interaction would likely be specific for the V0 complex, as the ATP6V1H subunit would be expected to sterically block RAB association in a fully assembled V-ATPase complex. It is conceivable that these RABs, which are typically present at very high copy number in cells (≥10^5-6^ copies per cell)^18^ and associated with the endosomal membrane through their lipidated C-termini, could interact with ATP6V0A1 in a manner that is membrane facilitated.

**Limitations of This Study**

Our approach does not address all aspects of the endosomal system interactome. Endosomes are highly dynamics structures that undergo a continuous process of maturation. While the purification method of choice recovered the largest number of well-known endosomal proteins (**Extended Data Fig. 1a**), our approach will miss endolysosomal proteins that are not associated with EEA1-positive endosomes in steady-state. Likewise, proteins that transiently associate with endosomes or peripheral proteins may be lost during the purification process. EndoMAP.v1 only represents a population-average of early endosomes that contain sufficient EEA1 to allow enrichment, and the identification of specific affinity handles in the future will be needed to cover earlier or later stages of endosomal maturation. Regarding our proteomic approaches, BN-MS is biased towards stable protein complexes and XL-MS is partly biased towards more abundant proteins. In addition, the formation of cross-links depends on the availability of accessible residues (Lysines in case of DSSO) in close proximity to the interaction interface. While cross-linkers with additional reactivity can help increasing the coverage (such as DHSO/DMTMM for acidic residues), portions of proteins lacking reactive amino acids will be missed, such as membrane-embedded regions. Regarding structural modeling, AlphaFold has limitations in the prediction of multiple conformations (open, closed, tethered), which may cause inappropriate cross-link distance constraints when the cross-links detected do not correspond to the conformation predicted. Moreover, the structural modeling of high-order complexes requires knowledge of subunit stoichiometry within target complexes and could benefit from the inclusion of all subunits during the AF prediction, which would require computational power (or longer processing time) for the larger complexes. Finally, a major challenge for data of this scale will be experimental validation of predicted structures, which will require focused structural analysis of individual complexes and assemblies. Together, these examples illustrate a direction of additional efforts for the comprehensive characterization of the endosomal architecture.

**REFERENCES**

18. Itzhak, D. N., Tyanova, S., Cox, J. & Borner, G. H. Global, quantitative and dynamic mapping of protein subcellular localization. *Elife* **5** (2016). <https://doi.org:10.7554/eLife.16950>

19. Park, H. *et al.* Spatial snapshots of amyloid precursor protein intramembrane processing via early endosome proteomics. *Nat Commun* **13**, 6112 (2022). <https://doi.org:10.1038/s41467-022-33881-x>

21. Stahl, K. *et al.* Modelling protein complexes with crosslinking mass spectrometry and deep learning. *Nat Commun* **15**, 7866 (2024). <https://doi.org:10.1038/s41467-024-51771-2>

22. Pu, J. *et al.* BORC, a multisubunit complex that regulates lysosome positioning. *Dev Cell* **33**, 176-188 (2015). <https://doi.org:10.1016/j.devcel.2015.02.011>

24. Paczkowski, J. E., Richardson, B. C. & Fromme, J. C. Cargo adaptors: structures illuminate mechanisms regulating vesicle biogenesis. *Trends Cell Biol* **25**, 408-416 (2015). <https://doi.org:10.1016/j.tcb.2015.02.005>

28. Huttlin, E. L. *et al.* Dual proteome-scale networks reveal cell-specific remodeling of the human interactome. *Cell* **184**, 3022-3040 e3028 (2021). <https://doi.org:10.1016/j.cell.2021.04.011>

31. Clasen, M. A. *et al.* Proteome-scale recombinant standards and a robust high-speed search engine to advance cross-linking MS-based interactomics. *Nat Methods* **21**, 2327-2335 (2024). <https://doi.org:10.1038/s41592-024-02478-1>

112. Cho, N. H. *et al.* OpenCell: Endogenous tagging for the cartography of human cellular organization. *Science* **375**, eabi6983 (2022). <https://doi.org:10.1126/science.abi6983>

148. Wang, L., Wu, D., Robinson, C. V. & Fu, T. M. Identification of mEAK-7 as a human V-ATPase regulator via cryo-EM data mining. *Proc Natl Acad Sci U S A* **119**, e2203742119 (2022). <https://doi.org:10.1073/pnas.2203742119>

149. Oot, R. A. & Wilkens, S. Human V-ATPase function is positively and negatively regulated by TLDc proteins. *Structure* (2024). <https://doi.org:10.1016/j.str.2024.03.009>
