# Supplementary material for: EndoMAP.v1 charts the structural landscape of human early endosome complexes
Source: Nature. 2025 May 28;643(8070):252–61. doi: 10.1038/s41586-025-09059-y (PMC12222028; doi:10.1038/s41586-025-09059-y)
Supplement: Supplementary file 4 — Supplementary Tables 1–6. [file 41586_2025_9059_MOESM4_ESM.zip › 2024-08-16909D-s4/SupplementaryTableLegends.docx]

**Supplementary Table 1.** Endosomal proteome scoring, label-free Endo-IP proteomics and disease over-representation analysis of the endosomal proteome.

**Sup. Table 1a.** Summary information of proteomics datasets included in the endosomal scoring method.

**Sup. Table 1b.** Proteins identified in each dataset and overlap with manual endosomal annotation. Presence (1) or absence (0) in each dataset is indicated.

**Sup. Table 1c.** Combined endosomal score from the number of datasets, Endo-IP abundance and number of endosomal protein-protein interactions (PPIs).

**Sup. Table 1d.** Known and predicted endosomal proteins from the scoring system and manually annotated endosomal proteins.

**Sup. Table 1e.** Label-free proteomic analysis using MS-DAP for evaluation of Endo-IP protocol. Biological triplicates of each sample were analyzed.

**Sup. Table 1f.** Enrichment analysis of gene-disease associations from disease gene network (DisGeNET) for endosomal proteins defined by our scoring method.

**Sup. Table 1g.** Enrichment analysis of gene-disease associations from disease gene network (DisGeNET) for endosomal proteins as annotated in Gene Ontology (GO:0005768).

**Sup. Table 1h.** Enrichment analysis within various classes of neurodegenerative disease-related proteins (LSD, Lysosomal Storage Disorders; ALS, Amyotrophic Lateral Sclerosis; ASD, Autism Spectrum Disorder; DD/ID, epilepsy and severe neurodevelopmental disorder) for endosomal proteins defined by scoring method and Gene Ontology (GO:0005768). Brain expressed genes were used as background.

**Supplementary Table 2.** Master dataset for construction of EndoMAP.v1 including proteomic data for individual BN-MS analyses, XL-MS analyses and AF analyses.

**Sup. Table 2a.** Blue-Native cofractionation mass spectrometry (BN-MS) analysis of purified endosomes using Endo-IP. Protein quantification of each fraction and replicate is indicated, as well as median-combined elution profiles.

**Sup. Table 2b.** PCprophet co-elution analysis of BN-MS data. Interaction scores are indicated for each replicate. Only protein interactions with >0.7 score in at least two replicates were considered. Estimated molecular weight based on gel migration is indicated. Interactions with available structures in PDB are indicated (1 for interactions present in PDB and 0 for absent).

**Sup. Table 2c.** Cross-linking mass spectrometry (XL-MS) analysis of purified endosomes using Endo-IP. Two independent replicates cross-linked with DSSO were included. Analysis performed with XlinkX and only cross-links with XlinkX score >40 were considered.

**Sup. Table 2d.** Re-analysis of XL-MS data from purified endosomes. Two independent replicates cross-linked with DSSO were included. Analysis performed with Scout using a 1% FDR cutoff on Residue Pair level.

**Sup. Table 2e.** SECAT co-elution analysis of BN-MS data targeted to the protein pairs identified by XL-MS.

**Sup. Table 2f.** Master table containing all results from endosomal scoring, BN-MS, XL-MS and AlphaFold (AF) analyses used to construct EndoMAP.v1. Previously reported protein-protein interactions in BioPlex, CORUM and STRINGDB are indicated. Protein complex and disease annotations used for network analysis are included.

**Sup. Table 2g.** Complete Gene Ontology enrichment analysis for each protein community identified in the main component of the EndoMAPV1 network. Only CORUM, GO:BP and GO:CC were included.

**Sup. Table 2h.** Additional DHSO cross-linking mass spectrometry (XL-MS) analysis of purified endosomes. XL-MS analysis of DHSO cross-linked peptides using Scout with a 1% FDR cutoff on Residue Pair level.

**Sup. Table 2i.** Additional DMTMM cross-linking mass spectrometry (XL-MS) analysis of purified endosomes. XL-MS analysis of DMTMM cross-linked peptides using pLink2 with a 1% FDR cutoff at PSM level.

**Supplementary Table 3.** Large-scale AlphaFold Multimer predictions of cross-linked proteins.

**Sup. Table 3a.** AlphaFold Multimer (AF-M) predictions of 4165 pairwise interactions identified by XL-MS with confidence measurements and SPOC score.

**Sup. Table 3b.** Mapping XL-MS data into AlphaFold Multimer predictions of pairwise interactions. Only predictions of interactions with no structural data (absent in PDB) involving endosomal proteins and SPOC score>0.33 are included. Only DSSO cross-links are included

**Sup. Table 3c.** AlphaLink2 predictions of 3886 pairwise interactions identified by XL-MS with confidence measurements and SPOC score.

**Sup. Table 3d.** Mapping XL-MS data into AlphaLink2 predictions of pairwise interactions. Only predictions of interactions with no structural data (absent in PDB) involving endosomal proteins and SPOC score>0.33 are included. DSSO, DHSO and DMTMM cross-links were included

**Sup. Table3e.** Protein-coding candidate disease variants located near interaction interfaces in AF-M predictions.

**Supplementary Table 4.** Proteomic analysis of TMEM230 interacting proteins and proteomic profiling WT, TMEM230^-/-^, and TMEM230^X121W^ iNeurons.

**Sup. Table 4a.** Proteomic analysis of TMEM230 immunoprecipitation in WT compared to TMEM230-/- iNeurons.

**Sup. Table 4b.** Proteomic analysis of HA-TMEM230 immunoprecipitation in TMEM230-/- iNeurons expressing HA-TMEM230 carrying disease mutations.

**Sup. Table 4c.** Proteomic analysis of postnuclear supernatant (PNS) from WT, TMEM230-/- and TMEM230-X121W iNeurons in biological triplicates.

**Sup. Table 4d.** Proteomic analysis of Endo-IP samples from WT, TMEM230-/- and TMEM230-X121W iNeurons in biological triplicates.

**Sup. Table 4e.** SynGO enrichment analysis of proteins significantly regulated in postnuclear supernatant (PNS) from TMEM230-X121W iNeurons.

**Sup. Table 4f.** SynGO enrichment analysis of proteins significantly regulated in early endosomes (Endo-IP) from TMEM230-X121W iNeurons.

**Supplementary Table 5.** Proteomic analysis of TMEM9 interacting proteins and proteomic profiling WT, TMEM9^-/-^, and two different clones of TMEM9/9B^DKO^ iNeurons.

**Sup. Table 5a.** Proteomic analysis of TMEM9-HA immunoprecipitation in TMEM9-/- iNeurons with or without expression of TMEM9-HA.

**Sup. Table 5b.** Proteomic analysis of postnuclear supernatant (PNS) from WT, TMEM9-/-, and two different clones of TMEM9/9B DKO iNeurons in biological triplicates.

**Sup. Table 5c.** Proteomic analysis of Endo-IP samples from WT, TMEM9-/-, and two different clones of TMEM9/9B DKO iNeurons in biological triplicates.

**Supplementary Table 6.** AlphaFold Multimer predictions of 3-way clique within EndoMAP.v1 and confidence measurements.

**Sup. Table 6a.** AlphaFold Multimer predictions of all 3-way cliques within EndoMAP.v1 with confidence measurements for each interface.

**Sup. Table 6b.** Mapping DSSO XL-MS data into AlphaFold Multimer predictions of 3-way interactions. Only predictions of interactions with at least 2 interfaces with an average model > 0.5 are included.
